# Systematic review on barriers and enablers for access to diabetic retinopathy screening services in different income settings

**DOI:** 10.1371/journal.pone.0198979

**Published:** 2019-04-23

**Authors:** Mapa Mudiyanselage Prabhath Nishantha Piyasena, Gudlavalleti Venkata S. Murthy, Jennifer L. Y. Yip, Clare Gilbert, Maria Zuurmond, Tunde Peto, Iris Gordon, Suwin Hewage, Sureshkumar Kamalakannan

**Affiliations:** 1 Clinical Research Department, Department of Infectious and Tropical Diseases, International Centre for Eye Health, London School of Hygiene and Tropical Medicine, London, United Kingdom; 2 Clinical Research Department, Department of Infectious and Tropical Diseases, International Centre for Evidence in Disability, London School of Hygiene and Tropical Medicine, London, United Kingdom; 3 Centre for Public Health, Faculty of Medicine, Health and Life Sciences, School of Medicine, Dentistry and Biomedical Sciences, Queen’s University, Belfast, Northern Ireland; 4 Retina Unit, Department of Vitreo-retina, National Eye Hospital, Colombo, Sri Lanka; 5 Department of Eye Health and Disability, Indian Institute of Public Health, Public Health Foundation of India, Hyderabad, Telangana, India; University of Debrecen, Faculty of Medicine, HUNGARY

## Abstract

**Background:**

Diabetic retinopathy (DR) can lead to visual impairment and blindness if not detected and treated in time. Knowing the barriers/enablers in advance in contrasting different country income settings may accelerate development of a successful DR screening (DRS) program. This would be especially applicable in the low-income settings with the rising prevalence of DR.

**Objectives:**

The aim of this systematic review is to identify and contrast the barriers/enablers to DRS for different contexts using both consumers i.e., people with diabetes (PwDM) and provider perspectives and system level factors in different country income settings.

**Methods:**

We searched MEDLINE, Embase, CENTRAL in the Cochrane Library from the databases start date to December 2018. We included the studies reported on barriers and enablers to access DRS services based at health care facilities. We categorised and synthesized themes related to the consumers (individuals), providers and the health systems (environment) as main dimensions according to the constructs of social cognitive theory, supported by the quantitative measures i.e., odds ratios as reported by each of the study authors.

**Main results:**

We included 77 studies primarily describing the barriers and enablers. Most of the studies were from high income settings (72.7%, 56/77) and cross sectional in design (76.6%, 59/77). From the perspectives of consumers, lack of knowledge, attitude, awareness and motivation were identified as major barriers. The enablers were fear of blindness, proximity of screening facility, experiences of vision loss and being concerned of eye complications. In providers’ perspectives, lack of skilled human resources, training programs, infrastructure of retinal imaging and cost of services were the main barriers. Higher odds of uptake of DRS services was observed when PwDM were provided health education (odds ratio (OR) 4.3) and having knowledge on DR (OR range 1.3–19.7).

**Conclusion:**

Knowing the barriers to access DRS is a pre-requisite in development of a successful screening program. The awareness, knowledge and attitude of the consumers, availability of skilled human resources and infrastructure emerged as the major barriers to access to DRS in any income setting.

## Introduction

Diabetes mellitus (DM) is one of the most prevalent non-communicable diseases which imposes a significant impact on health systems. The International Diabetes Federation (IDF) estimated that there were 425 million people with diabetes (PwDM) in the world in year 2017 and this will increase to 629 million by 2045 [1]. It has been emphasised that efforts should be made to prevent the complications of DM as per the targets set in St Vincent declaration in 1989 [2]. It was targeted to reduce the blindness due to diabetic retinopathy (DR) by one third, by raising awareness among the PwDM and by improving the capacity to deliver services by the providers. DR is a common microvascular complication of the eyes caused by chronic hyperglycaemia. Blindness due to DR is common among the working age populations and it is becoming a global issue due to rising prevalence of DM [3]. Though proportion of blindness due to DR is low compared to other causes of blindness, expenditure related to DR is a burden to any health system [4].

Penchansky and Thomas described the concept of access as the *“degree of fit”* between clients and the health system [5]. Healthcare access for PwDM has an especially significant role in the prevention of sight loss due to DR. Access to health care remained a vague concept, until recently, impeding the work of health care policy makers. Optimal access to health care was defined by Rogers et al., (1999) as *“providing the right service at the right time in the right place”* [6]. In generic literature it is mentioned that access has multiple dimensions and it is not merely the entry in to the healthcare system [5]. Further it is an outcome of people’s potential to use health care and manifestations of patients actual use [7]. Donabedian has observed that the proof of access is use of service, not simply the presence of a facility [8]. Some authors argue that it depends on acceptability of the services as well [9,10]. It is mentioned that inequalities have been observed in detection and treatment of DR which require multi-sectoral engagement [11]. Further, universal coverage cannot be achieved without addressing the barriers [12]. One review mentioned that there are many reasons for underutilization of eyecare and that the risk of blindness varies with the context [13]. It has been shown that culturally competent care should be delivered in a diverse patient community overcoming the sociocultural barriers [14,15]. This is especially relevant with regard to healthcare delivery for DR.

Screening of DR can be done opportunistically or proactively. Current literature shows that proper disease control of DM, diabetic retinopathy screening (DRS) and early identification and treatment of pathologies will reduce progression of sight threatening DR (STDR) [16–20]. Awareness of the need for detecting DR at a symptomless stage is a key factor in uptake and regular follow up of DRS services [21]. There are many obstacles for implementation and maintaining satisfactory level of uptake in DRS at a program level. One important approach to address this issue is identification of barriers and enablers in the system in advance. The barriers to access DRS could vary according to the country income level and various system factors in each setting. Different economic and socio-cultural factors would affect the access. Knowing the impeders in each setting will enable successful implantation of DRS strategies. Especially this will enable to identify effective strategies for low and middle-income settings, as we can expect a rise in number with DR in future [1]. Defining a barrier will enable implementation of public health strategies to improve access [5]. The barriers such as lack of knowledge and awareness on DR by the consumers and lack of training, skills and screening equipment for the providers would impede the access to DRS. A barrier could lead to a different outcome for a certain community such as difficulties in mobility for people with disabilities [22]. In system assessments authors mentioned that economic and logistic reasons hinder the provision of screening services [23]. Yet, it is mentioned that effective strategies are frequently underutilised in developing countries to overcome such barriers [24]. Therefore, it is necessary to understand the potential barriers in accessing and challenges in provision of DRS services in any health system.

The successful uptake of DRS services depends on the personal factors related to the consumer as an agency [25]. These factors may be modifiable or not modifiable according to the environment. The required behavioural change techniques for a target population could be hypothesised using various behavioural models. The “*social cognitive theory*” explains how persons acquire and maintain specific behavioural patterns and it provides the basis of most intervention strategies to overcome a defined barrier [26]. A person’s behaviour influences and is influenced by personal factors and the social environment (‘Reciprocal determinism’) [27]. This will lead to self-efficacy of the person to achieve confidence for performing a particular behaviour. We hypothesised that identification of barriers at individual PwDM and provider / system level as the environment would enable to identify the impeders in advance. Therefore, assessment of behavioural patterns and perceived barriers in accessing DRS services may be useful in developing strategies for a successful DRS program in any context.

The current evidence provides information on barriers without considering the settings. However, barriers to access DRS are different in various country income levels and health systems. One review described interventions to promote DRS uptake [28]. In addition, there was another Cochrane review on quality improvement interventions to increase DRS attendance [29]. Another recently published review has also considered the barriers to access DRS without specifying the setting [30]. In addition, this review mostly focused on improving the attendance at existing services. In our review we explored the barriers in broader dimensions including planning and implementation especially in low income settings, without limiting to attendance. More over most of the available reviews described barriers based on modifiable themes or factors that would affect DRS uptake. However, we proposed to identify non-modifiable barriers as well, since this knowledge will be useful to identify the defaulters / those who are at risk of sight loss in advance. In addition, another review has included studies only after the year 2003 considering the effective implementation of programs following ‘St Vincent Declaration’ [31]. However this review was then limited to the studies only from high income countries (HIC) since most of the programmes were implemented in European countries [31].

Most of the available studies had provided the evidence of barriers to access DRS services according to the presumed typology of barriers. The processes related to DRS uptake can be considered at three levels i.e., consumer, service provider and eyecare system. Therefore, in this review we categorised the reported themes or variables under above categories. We specifically tried to assess the challenges faced by the providers at established healthcare facilities that have DRS services, in addition to studying barriers for consumers. In broad definitions, barriers to access to DRS are not only limited to the access issues at the point of delivery, but it also involves all the steps which take place starting from perceptions of a PwDM at one end to the whole eye care system at the other end which are inter-related and connected to each other.

### Objectives

The overall aim of the review was to explore barriers to access DRS in various country income settings. The review has the following specific objectives.

- To assess the barriers and enablers to uptake of DRS services by PwDM by country income category.
- To assess the challenges faced by the services providers in provision of DRS services and to identify the enablers for development of a DRS program in each setting.

The secondary objectives of this review were;

- To assess the socio-demographic and economic factors that could affect DRS uptake.
- To assess the barriers or enablers to develop a DRS program in a health care system.

## Methods

We included studies that focused on assessing barriers and enablers to access DRS. In addition, we found studies that described factors affecting the uptake of DRS services. Following criteria were used for assessment of eligibility of the studies. (There is no protocol registration for this review and Preferred Reporting Items for Systematic Reviews and Meta-Analyses (PRISMA) checklist was included as S1 Table).

Inclusion of studies -

- Consumers—The studies which have assessed the barriers at group or individual level of PwDM at or who had been referred to a permanent health care facility for DRS.
- Service providers—Studies in which participants were service providers who have direct contact with PwDM in a permanent health care institution and / or clinical decision makers / other stake holders involved in DRS service related decision making.

Exclusion of studies -

- Studies which have obtained the study sample from the general population without specifying the status of DM.
- Absence of standard diagnostic criteria for DM.
- Studies assessing barriers for eye care in general, without specifying DRS.
- Studies assessing barriers for screening DM complications in general, without specifying the barriers for DRS.

We did not restrict the studies for inclusion by study design. We included studies that used qualitative, quantitative and mixed methods.

### Type of participants

We included the studies that have covered PwDM who were attending an existing DRS program, diabetic medical care or an eye care facility.

### Type of interventions

We included studies that delivered or considered DRS primarily at an established health care facility. We defined the DRS as performance of dilated retinal screening using imaging (digital / colour films) or by direct / indirect ophthalmoscopy by a trained / skilled eye care professional (preferably an ophthalmologist / retinologist) to identify the signs of DR.

### Type of outcome measures

We defined access as all level of factors affecting the processes of DRS in a health care facility.

### Phenomena of interest

We included the studies which have assessed barriers or enablers to access DRS by PwDM and challenges or incentives faced by providers in provision of screening services in current screening programs or at opportunistic screening.

### Search method for the identification of studies

We searched Ovid MEDLINE, Embase and CENTRAL in the Cochrane Library from the databases inception up to 15^th^ December 2018. The search strategy was developed by an information specialist from Cochrane Eyes and Vision (IG) (search terms available in S2 Table). We did not use any filtering methods to limit the results by study design, year of publication or language. This yielded a comprehensive coverage of published articles. However due to resource restrains we were not able to translate any non-English reports.

## Data collection and analysis

Two reviewers (PN and SK) independently assessed the eligibility of inclusion by going through titles and abstracts of 16,388 articles after, importing them to an EndNote library. The potential articles (full papers as identified by either or both reviewers) were retrieved from publishers. These papers were then assessed independently by the two reviewers (PN and SK). Disagreements between the reviewers were resolved by a 3^rd^ arbitrary reviewer (GV). Reviewers assessed full papers independently to retrieve accurate data. We aimed to include all relevant studies from different income settings to avoid bias in selecting articles. Therefore, we were able to extract a range of barrier themes with a greater variation and a greater conceptual diversity.

### Data extraction and management

We developed an MS Office Excel data sheet to directly transfer extracted data from full articles. The topics to be extracted were developed according the “Strengthening the Reporting of Observational Studies in Epidemiology” (STROBE) statement and modelling has been done according to the review question [32]. The accuracy of extracted data was cross-checked by a third reviewer (SH).

We extracted information on first author’s name, year of publication, country of study (by income category), place of the study, sample size, gender distribution, mean age, method of diagnosing DM, level of DM and DR of the participants and method of DRS in the 1^st^ set of data. In the next step we collected information on type of study design, objective, study setting, data sources, sampling strategy and time period when study was conducted. The methodological quality assessment and applicability for review question were done separately as subsequently described. We extracted the results and main outcomes of each study according to the review question.

In the synthesis of evidence “informants” were authors of the individual studies rather than the participants. The authors’ interpretations were presented as narrative themes supported by numerical values of statistical significance levels wherever available.

While authors’ interpretations were primarily collected from the results section of each paper, sometimes interpretations were also found in the discussion section. These were also extracted when relevant and if adequately supported by data. Finally, we tabulated the results by level of income of the country according to the World Bank 2016 classification.

### Assessment of risk of bias in included articles

We carried out the risk of bias and quality assessment according to the guidelines of critical appraisal of skills program (CASP) tools for case-control, qualitative, cohort and randomised controlled study designs [33] and National Institute of Health, United States quality assessment tool (NIH-QAT) for observational cohort and cross sectional study designs [34]. Two reviewers (SH and PN) independently applied the set of quality criteria to each included study. We appraised how well the individual studies which contributed to narrative synthesis, were conducted using the above tools. Emphasis was given more to the applicability of the study according to the inclusion criteria. It has been noted that applicability to the review question was the main concern in the synthesis rather than the overall level of quality of a study (S3 Table).

### Assessment of methodological limitations

When several studies with varied methodological limitations contributed to a finding, we made an overall judgement about the distribution of strengths and weaknesses of the study rather than for individual components in the tools.

### Assessing coherence

We assessed the coherence of each review finding by looking at extent to which we could identify a clear pattern across the data contributed by each of the individual studies. This was supported by when clarity of the themes was consistent across different contexts and the variations were explained by the study authors according to the data collected, when supported by numerical data (odds ratios). This was further strengthened when findings were drawn from different settings.

### Data synthesis

Most of the eligible studies were observational and descriptive in nature hence narrative reporting approach was used to generate new insights. We analysed and synthesised the descriptive and qualitative data narratively supported by other associated variables with levels of statistical significance. We described the barriers and enablers according to the dimensions of the typology of barriers and this in turn was tied with processes involved in DRS. We followed a content analysis, by developing themes a priori and tabulation and frequency counting to identify the major themes. We considered consumer, provider and system factors as the major constructs according to the social cognitive theory. Themes were presented graphically using harvest plots. When describing the themes, we did not re-phrase the original findings or conclusions mentioned by authors. We used imputations up to a certain degree in describing enabler themes.

Considering the participants of the studies, the themes that emerged were divided in to three categories complying with the objectives of the systematic reviews. These categories were consumer perspectives, provider perspectives and system factors. We assumed that this type of decomposition will be helpful to commission to inform strategies for development of a successful program and enhance the policy relevance and applications.

## Results

### Results of the search

**Fig 1:**
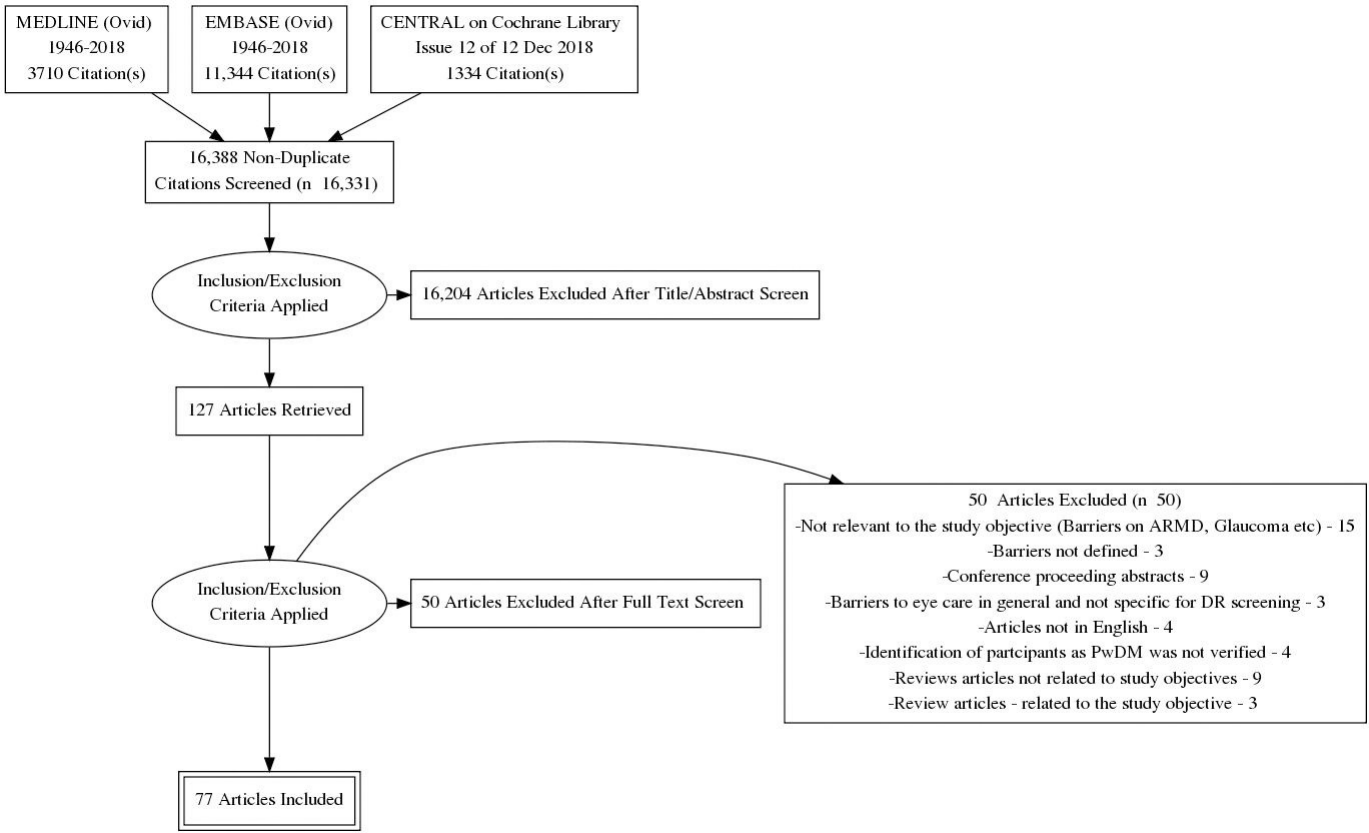
PRISMA flow chart.

Search and study selection procedures are summarized in the PRISMA flow diagram (Fig 1). The database search identified a total of 16,388 records. Duplicate records were removed, and we assessed 16,331 titles and abstracts for potential inclusion in the review. We excluded 16,204 records based on the information given in the title and abstract. After assessing the full-text of 127 reports of studies, we excluded 50 studies which did not meet the inclusion criteria and included a total of 77 studies in the review.

### Overview of the included studies

We identified a total of 16,331 titles and abstracts and considered 127 full text papers for inclusion in this review and data were extracted from 127 full reports. Seventy-seven (77/127, 60.6%) studies were eligible for inclusion in the narrative review according to the objectives. The S6 Table file contains the details of participants and settings.

### Included studies

This analysis mainly comprised of cross-sectional observational studies. In the included 77 studies, there were 59 (59/77, 76.6%) cross sectional observational studies (observational 33, retrospective studies 8, postal surveys 1, telephone interview 2, 1 mixed method audit and 14 population-based studies (14/77, 18.1%)). Other study designs were 3 controlled trials (3/77, 3.9%),1 case control study (1/77, 1.3%), 4 cohort studies (4/77, 5.2%) and 8 qualitative studies (8/77, 10.4%). There were also 2 reviews (2/77, 2.6%) in the included studies.

### Methodological quality of the studies

The methodological quality assessments of included studies are presented in S3 Table according to the study design. In the included cross-sectional studies, 96% (57/59) of the studies clearly stated study objective matching the review question. Sample size justification was not available in 51% (30/59) of the studies. Participation of eligible persons i.e., facility based diagnosed PwDM, at least 50% was not seen in seven (7/59, 12%) studies and eight studies (8/59, 13%) did not report on this aspect. Four of the studies (4/59, 7%) had not recruited the participants from a similar population. The outcome measures were not clearly defined in 14 studies (14/59, 24%) and confounders were not adjusted in eleven studies (11/59, 19%).

An acceptable method of recruitment of the cohort was not followed in all four of the included cohort studies. In included randomised controlled study designs, applicability of the results to the PwDM was not observed in two studies (2/3, 66%). In qualitative study designs, most of the quality assessment criteria were met except, relationship between researcher and the participants were not adequately considered in two studies (2/8, 25%) and in one study (1/8, 12.5%) recruitment strategy was inappropriate; i.e., those who had worse vision (no perception to light in any eye) had been excluded from the qualitative interviews. There was one case-control design with appropriate methodology.

### Study populations/groups

All the studies main group of respondents were PwDM. Some authors have sought barrier perspectives form providers as well. Fifty-four studies (70.1%, 54/77) described barriers related to consumers, providers and eye care system, 3 studies on consumers and system (3.9%, 3/77), 2 studies on provider and system (2.6%, 2/77) and 12 studies on consumer and provider (15.6%, 12/77). Only 5 studies (6.5%, 5/77) described barriers of consumers only and one study has focused only on providers (1.3%, 1/77). In these 77 studies, two studies reported the outcome as a review [35,36].

### Study Settings-by income

Only three (4.8%, 3/63) studies were from low income countries (LIC) (Sub-Saharan Africa (as a review), Tanzania and Nepal) [35,37,38]. Eleven were from lower middle income countries (LMIC) (14.2%, 11/77) (Indonesia, India, Yemen, Kenya, Myanmar, Nigeria and Bangladesh) [39–49], seven from upper middle income countries (UMIC) (9.1%, 7/77) (Turkey, Iran, Mediterranean countries and China) [50–56] and 56 from HICs (72.7%, 56/77); (17/77–22.1% from United Kingdom, 20/77–25.9% from United States, Other 40/77–51.9%—Germany, France, Ireland, Singapore, Canada, Oman, Hong Kong, South Korea, Australia, Taiwan, Italy and Netherland) [21,57–111].

### Setting-by type of institution

Most of the data collections were done under the primary level general practices, local clinics, rural outreach clinics and primary care clinics (20/77, 25.9%) [44,48,49,59,60,63,66,67,69,71,85, 89,92,93, 95,102,105,106,109,110]. There were 14 population-based studies (14/77, 18.1%) [45, 54, 55, 61,62, 75,76,79,81,88,90,91,98,100]. Eleven studies were conducted at tertiary level institutions (11/77, 14.3% - 8 eye clinics, 1 diabetic clinic, 1 general medical clinic and 1 endocrinology clinic) [37–39, 41,46,47,50,53,99,111,112]. Seven studies were conducted in existing DRS programs (7/77, 9.1%) [64,86,87,96,97,103,107]. Nine studies were conducted at secondary level medical and diabetes clinics (9/77, 12.7%) [43,51,68,70,74,84,104,108,113].

Five studies were conducted by analysing existing data bases (5/77, 6.5%) [72,77,78,80,83]. There was one study where authors did not mention about the setting, however we could assume it was at an ophthalmologist clinic through an insurance scheme [73] and two studies reported the barriers as a review [35,36]. Two studies collected the sample of PwDM at a screening camp and at an annual campaign.[40,101]. One study was conducted in a an ambulatory clinic based at a nursing home [57]. Three studies conducted at eye clinics (3/77, 3.9%; 2 at general eye clinic [42,94] and 1 optometry practice [82]. One study conducted using at a model of not for profit health model [65]. A study exclusively on providers’ perspectives recruited stakeholders at national level [56].

### Synthesis

Our main objective was to identify barriers or enablers to access DRS. Our findings are summarised in the S4 and S5 Tables files according to the country income.

## Narrative summary—Barriers

The following main themes were derived from descriptive and qualitative studies (S5 Table).

### Low income countries

The most prominent barriers to access DRS among the consumers in LIC were lack of knowledge on DM eye complications, lack of awareness about importance of eye examination and lack of knowledge about availability of eye clinics. Among providers, main challenges were lack of skilled human resources and lack of access to DR imaging and treatment infrastructure. Further, non-existence of a referral system and lack of multi-disciplinary care approach were barriers to provision of DRS services. In LIC, lack of a national policy and competing disease priority environments were the main obstacles in the system (S5 Table).

### Lower middle-income countries

Consumers’ barriers related to knowledge and awareness could be observed in the LMIC as well. This was associated with poor general education and low functional health literacy. Most of the studies found that health beliefs such as no need of screening at asymptomatic stage, misconceptions on DR and unawareness of the need for regular screening affected the attitude of uptake of services. In addition, studies with PwDM reported that lack of time and lack of family support hindered access. Additionally, financial barriers and disabilities emerged as themes of barriers.

In providers perspectives lack of DM health education and financial constraints were the main barriers. Lack of human resources, uneven distribution of skilled personnel, lack of availability of equipment i.e., imaging technology, DRS related consumables such as pupil dilating drops and treatment facilities were observed as main service provider barriers. Some studies reported that lack of knowledge and awareness on DR among the physicians, lack of skills in identifying DR as well. In addition, low referral rates and time constraints in busy eye clinics were the main challenges faced by providers in LMICs in provision of DRS services. In system analysis lack of training, lack of accessible eye centres, poor public transportation systems and lack of epidemiological studies were emerged as main barriers (S5 Table).

### Upper middle-income countries

The lack of awareness and knowledge on DR emerged as the main barrier among the PwDM in UMIC. This was associated with low literary and poor educational levels in UMIC as well. Poor physician-patient communication was also a barrier in these countries. In provider perspectives scarce human resources, lack of training, high number of PwDM were the main challenges faced. In addition, poor provider awareness on screening guidelines and lack of imaging technology hindered provision of services. In the system analysis limitations in prevention and health promotion, poor usage of prevalence data, lack of information systems, lack of auditing systems, civil unrest, disparity in urban and rural services, lack of transportation and problems in insurance schemes were the main barriers to accessing DRS services (S5 Table).

### High income countries

In HIC living alone, problems in mobility, multiple comorbidities, negative self-perceptions, problems in accessing general practitioner, effects of mydriasis prohibiting driving, reluctance to change behaviour, disliking the method of examination, change of residence, problems in securing appointments, being employed, extended vacations were observed as the main barriers among the consumers. In some HIC, lack of knowledge regarding eye examination, lack of knowledge on need of screening during asymptomatic stage, misconceptions, lack of awareness of eye care, lack of flexibility in adjusting attitude and behaviour and lack of understanding of rationale and importance of annual eye examination were observed as barriers to access DRS services. Even in the HIC socio-economic inequalities, poor communication skills, social deprivation and poorer literacy were barriers to access DRS services among some communities.

In the providers perspectives; level of experience of the screener, lack of attention by the general practitioners, non-adherence to guidelines, lack of information provided to patients, lack of physician recommendations, lack of coordination between general practitioners and screeners, limited knowledge on DR among the health professionals, long waiting time (large number of patients per doctor), failure to refer by general practitioner, perceptions of side effects of mydriasis, limited knowledge-attitude and practice of physicians, limited experience in using ophthalmoscope, long waiting time for treatment, lack of communication between screening services and practices were mentioned as barriers. Providers mentioned that problems associated with consumers such as confused and immobile patients, unawareness of importance of mydriasis, poor physician-patient communication, different perceptions in making appointments, after effects of mydriasis, fear of laser, and wrong assumption on patient’s level of knowledge could hinder to access DRS services.

In HIC system analysis lack of understanding among the specialities, frequent change of staff, lack of human resources, unavailability of medical records, lack of adequately trained optometrists, lack of proper referral and reminding system, lack of insurance coverage, financial barriers, unavailability of national programs, problems in transportation and lack of screening programs in remote areas were barriers to access or provision of DRS. The studies reported that among system factors, integration of screening to the general health systems, governance, quality and safety should be considered in conducting screening programs. (S5 Table).

### Overall barriers themes using harvest plots

**Fig 2:**
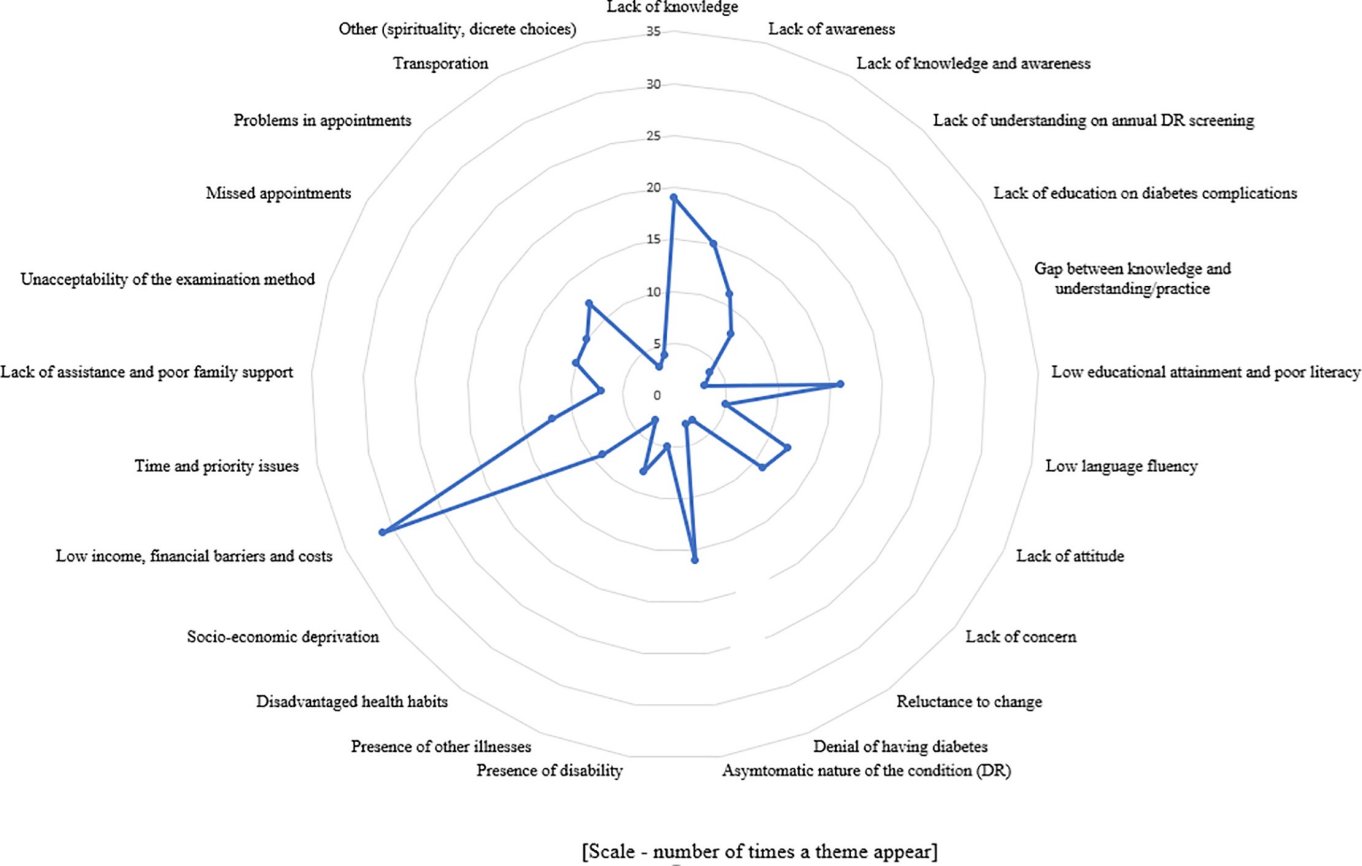
Harvest plot showing user barriers.

**Fig 3:**
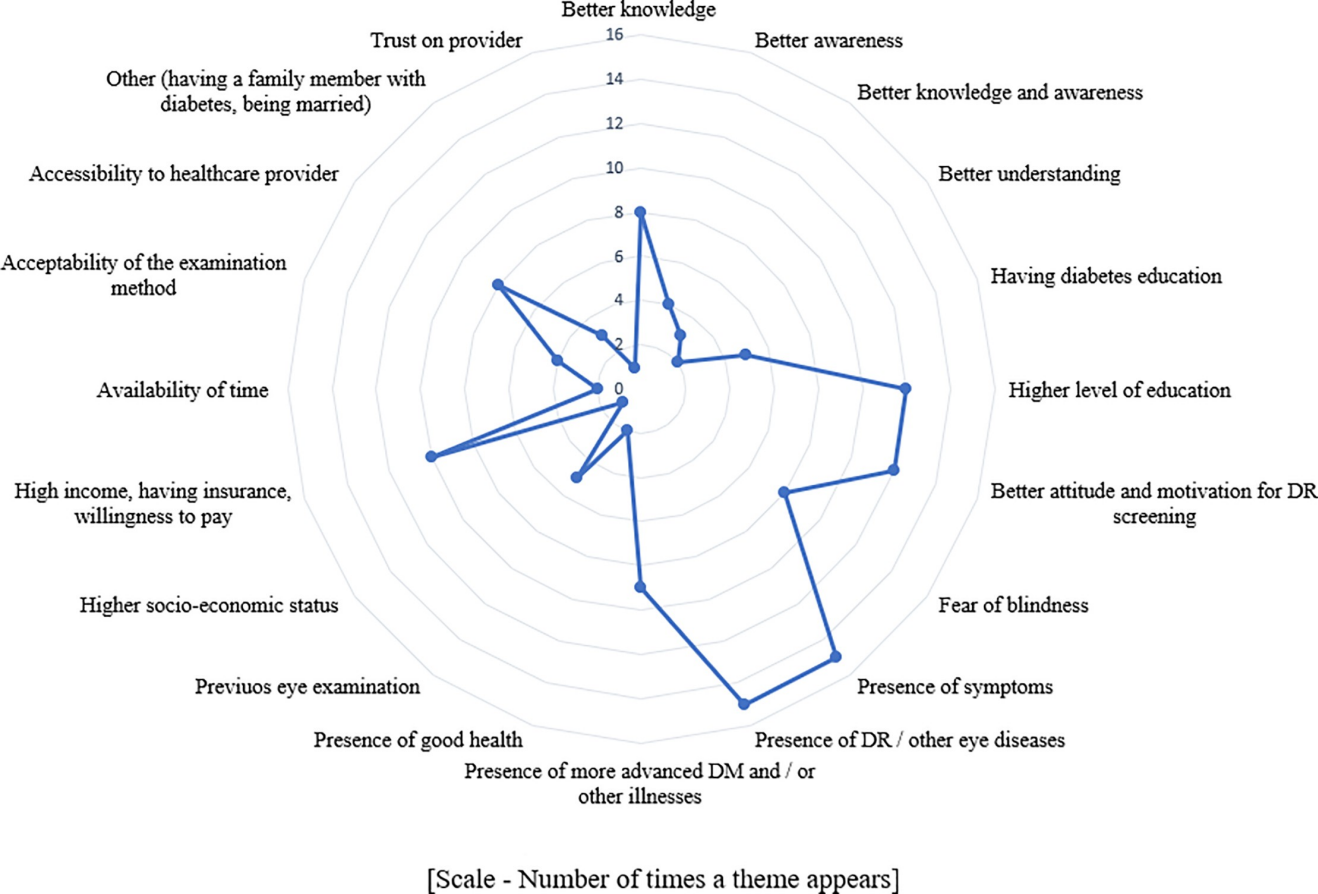
Harvest plot showing user incentives.

Considering the number of times a theme appeared irrespective of the country income level; lack of knowledge (19/77 studies, 25%), lack of awareness (15/77, 20%), low educational attainment and poor literacy (16/77, 21%), asymptomatic nature of DR (16/77, 21%), financial barriers (31/77, 40%) and time and priority issues (12/77, 16%) emerged as the major themes at consumer level. Similarly, at the provider level accessibility issues related with appointments (23/77, 30%), lack of human resources (10/77, 13%), lack of knowledge and awareness among the providers (11/77, 14%), lack of screening infrastructure (11/77, 14%), cost of services (11/77, 14%) and deficiencies in educating the users (21/77, 27%) reported as major barriers (Figs 2 and 3).

## Narrative summary—Enablers

Themes of enablers are summarised in the S5 Table files according to the country income category.

### Low income countries

In LIC settings consumers’ knowledge on DR, having a family member with DM and prior fundus examination were enablers to attend DRS. Provision of imaging and treatment infrastructure, increased human resources, provision of training on retinal care and prioritisation of development of subspecialties were mentioned as enablers for the providers (S5 Table).

### Lower middle-income countries

The enablers for uptake of services by the consumers were presence of symptoms, more severe DM and comorbidities, better understanding of risk factors and detrimental effects, patient satisfaction over the modality of screening and presence of visual impairment / blindness. Training of non-ophthalmologist physicians on DRS, availability of fundus camera, educational strategies aimed at both patients and physicians, reminders of the serious consequences of failure to undergo DRS, availability of written communication when referring for screening and public health education using media were emerged as enablers to improve uptake of DRS services in LMIC. (S5 Table).

### Upper middle-income countries

Higher literacy, person’s concern about the vision loss, severe DR stage and having knowledge on DR were the main enablers for users of DRS services uptake. In UMIC awareness among the physicians DM complications, availability of referral guidelines, availability of continuous medical education programs, training of human resources, involvement of community groups and community-based health education were enablers to improve DRS services by provider side (S5 Table).

### High income countries

The main enablers for screening uptake in HICs were awareness of eye care and possibility of treating DR, positive reinforcement through negative screening results, worrying about vision loss, attending DM education classes, discussion of DM complications with health care professionals, trust on provider, having health insurance with eye care services coverage, higher level of education and being obliged to attend for screening. Adherence to the best practice guidelines by the consumers and having eyes examined by primary care physicians were enablers.

In HIC availability of educational interventions, DM education programs, adherence to guidelines, targeted screening of high-risk groups, reinforcing the importance of eye examination by health care providers, constant screening location, personalised strategies on follow up (phone calls and door to door visits), on-line patient access booking system, recall system, showing fundus photograph and teaching patients, ability to change appointments were incentives for uptake of DRS services. The studies conducted in remote areas reported that mobile tele-screening models it-self was an enabler to improve access (S5 Table).

### Overall enabler themes using harvest plots

**Fig 4:**
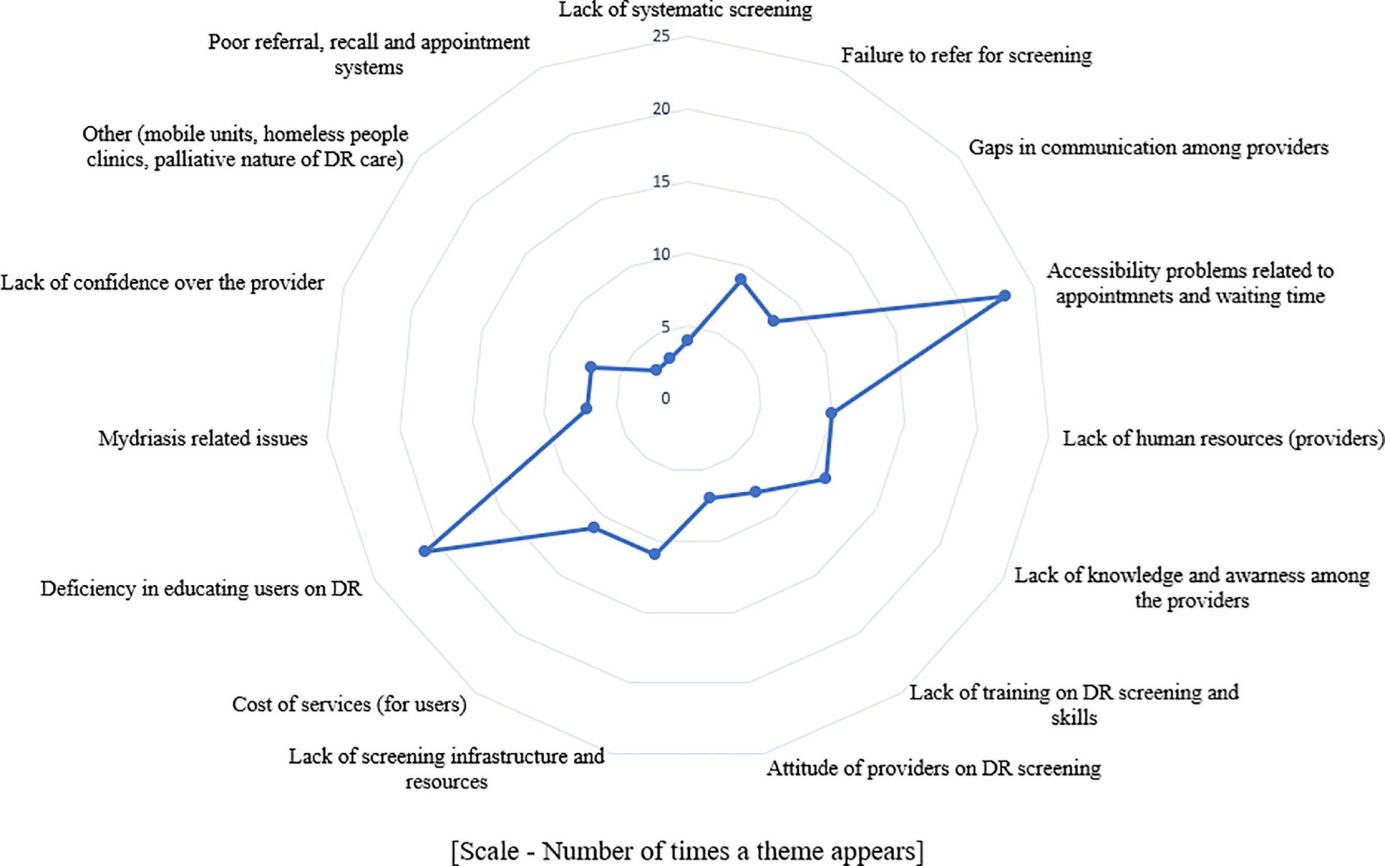
Harvest plot showing provider barriers.

**Fig 5:**
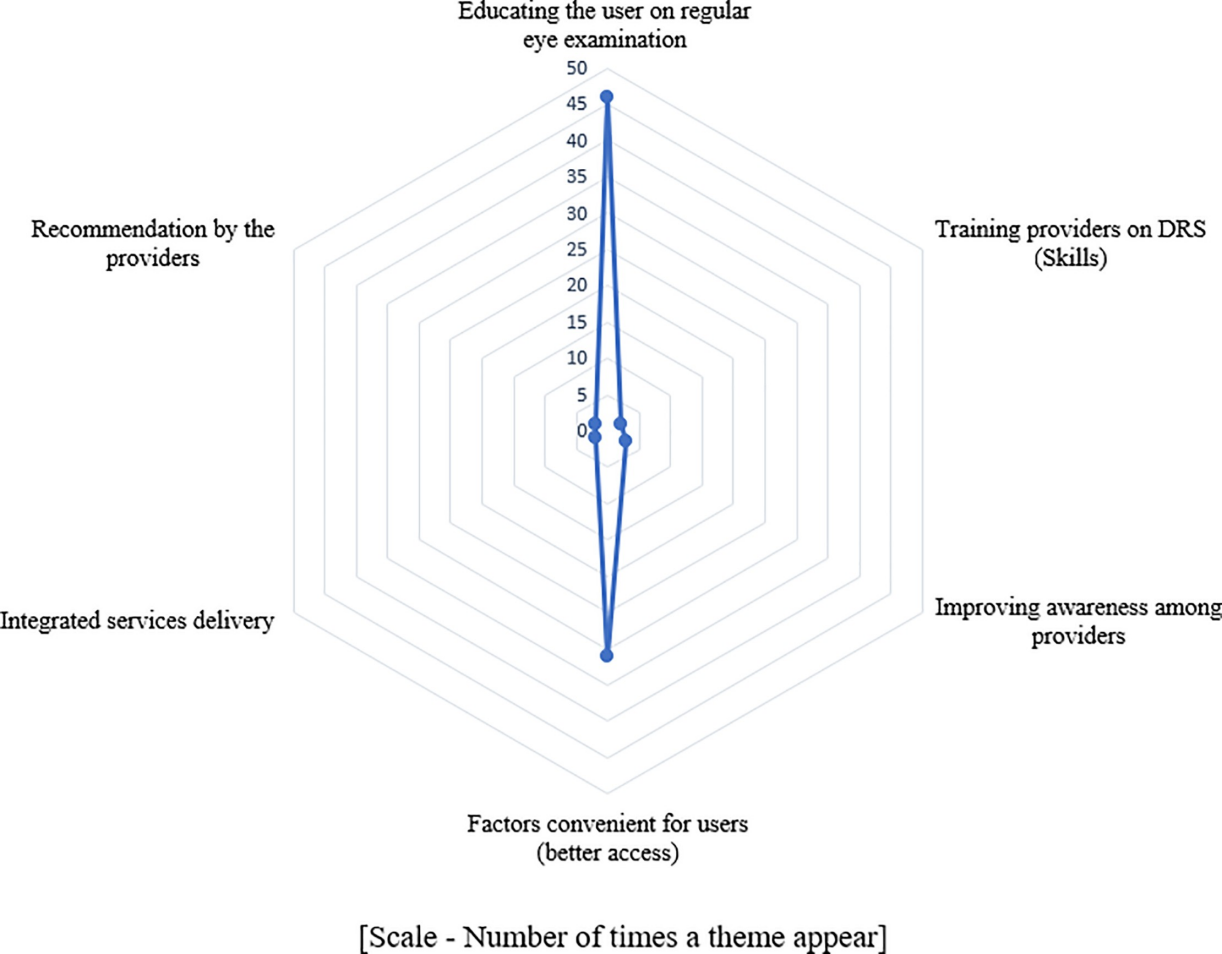
Harvest plot showing provider incentives.

Considering the number of times, a theme appeared irrespective of the country income level; presence of symptoms (15/77, 19%), presence of DR or other eye diseases (15/77, 19%), higher level of education (12/77, 15%), better attitude (12/77, 15%) and high income (10/77, 13%) were the main enablers for the PwDM. In providers perspective having health education on regular eye examination (46/77, 60%) and factors convenient for the users (31/77, 40%) were main enablers (Figs 4 and 5).

## Quantitative data synthesis

The data extracted for quantitative synthesis are available as supporting information S5 Table file.

### Knowledge and awareness

The main barriers identified in this systemic review were factors associated with consumers. The most consistent barrier across most of the studies was knowledge regarding DR. One study mentioned that knowledge (mean knowledge score 4.7 among those who had examination vs 3.6 without examination (p<0.001) and awareness about DR was associated with seeking screening services (odds ratio (OR) 1.52, 95%CI 1.1–2.1, p = 0.01) [39]. This concept was further emphasised in a randomised controlled trial conducted to evaluate the effectiveness of a health educational intervention, where the intervention arm participants had higher odds of eye examination status (OR 4.3, 95% CI 2.4–7.8) [68]. A study from China showed that having a higher DR knowledge score was a potential predictor for ever had an eye examination. (OR 1.31, 95% CI 1.1–1.5, P<0.001) [53]. Similarly, a study conducted in Bangladesh reported that awareness of DM (OR 8.47, 95% CI 3.95–18.18) and DR (OR 5.15, 95% CI 1.89–14.01) were associated with improved uptake of DRS services [48].

In a study conducted to enhance the compliance with DRS recommendations, it was seen that when PwDM were given educational material and a notification there was a significant difference in screening uptake (OR 1.4, McNemars X^2^ = 102.7; P < 0.0001) [114]. A study conducted in Tanzania showed that those who had knowledge on damages to eye due to DM had higher odds of undergoing dilated fundus examination in the past year (OR 19.7, 95% CI 7.0–55.2) [38].

Even the knowledge on DM alone was associated with uptake of DRS. A study mentioned that less practical knowledge about DM (OR 1.5, 95% CI 1.2–2.1) was a factor associated with non-adherence [88]. A similar finding was reported in the study conducted by Srinivas et al., (2017) in India, which shows good knowledge of DM was associated with good practice of DR (OR 3.95, 95% CI 1.97–7.94 p<0.01) [41]. On the other hand another study mentioned that knowledge on effects of DR on vision was an incentive for uptake of DRS (OR 3.3, 95% CI 2.0–5.5) [93]. It was seen that awareness on possibility of treating DR was an incentive for attending screening (OR 1.6, 95% CI 0.9–3.0) [93]. In contrast, a study conducted in Nigeria showed that associations between knowledge, attitude and practice and authors concluded that there was no significant correlation between knowledge and practice (correlation coefficient r = 0.086, p = 0.385) [47].

### Factors associated with awareness

Most of these studies had analysed the various patient characteristics and disease factors associated with awareness. Lack of awareness was associated with older age (OR 10.4, p = 0.03), poorly controlled HbA1c (OR 4.9, p<0.001) and male gender (OR 1.2, 95% CI 0.7–1.8, p = 0.47) [61]. Huang et al., (2013) showed that unawareness was associated with lower education (primary or less, adjusted OR 1.9, 95% CI 1.4–2.5, p<0.0001), lower income (Singapore $ <2000, adjusted OR 1.7, 95% CI 1.2–2.5, p = 0.003) and poorer literacy (unable to write—adjusted OR 1.4, 95% CI 1.0–2.0, p = 0.03) [115]. Katibeh et al., (2017) also showed that a good level of awareness on DR was associated with secondary or higher education (OR 1.88, 95% CI 1.23–2.88, p = 0.004) [55]. Thapa et al., (2012) mentioned that literate patients are more likely to have awareness on DR (OR 2.7, 95% CI 1.3–5.6, p = 0.006) [37]. One study showed that higher awareness on DR was seen among the more educated people (OR 1.8, 95% CI 0. 9–3.4, p = 0.0000) [54]. Those who have a history of prior fundus evaluation elsewhere (other than retinal clinics in this study) had higher odds of having awareness on DR (OR 11.9, 95% CI 5.7–25.2) p<0.001) [37].

### Attitude

It was also reported that PwDM themselves may not have the judgemental ability over seeking care and recommendation by the provider would improve access (OR 341, 95%CI 164–715, proportion of attendees 99.4% vs non-attendees 34.5%) [93]. In health seeking behaviour, those who thought eye examinations were needed every 6 months (OR 1.2, 95% CI 1.1–1.4) and those who worry much regarding their vision (following telephone call intervention, OR 3.47, 95% CI 1.8–6.8) showed higher odds of DRS uptake [108,116]. A study showed that perception of a PwDM should have eye examination every 12 months (OR 2.62, 95% CI 1.7–4.1, p<0.0001) was associated with previous dilated eye examination [71]. Another study showed that fear among patients on impaired vision was an incentive for DRS (OR 1.9, 95% CI 1.5–2.5) [93]. Lian et al., (2018) reported that those who worry more about vision loss were highly likely to attend screening (OR = 1.72, 95% CI 1.31–22.26, p<0.001) [105].

## Secondary outcomes of quantitative data—Factors associated with uptake of screening / adherence / regular follow up

### Service user costs

One major factor associated with undergoing DRS was having an insurance scheme. This was observed mainly in the paid systems, when services are delivered at a user fee and health services were not available free of charge. Having an insurance coverage either national or private, depending on the context, was associated with compliance for annual eye examination (National health insurance, OR 2.2, 95% CI 1.2–4.3 p = 0.02) [89], increased eye screening (private health insurance, OR 3.2, 95% CI 2.2–4.7, p = 0.00)[62] and higher chance of undergoing screening (health insurance—type not specified, adjusted OR 1.7, 95% CI 1.4–2.2)[78]. It is shown that those who have vision loss (blindness) are 100% willing to pay for the services (mean amount willing to pay-No DR—Taiwan dollars (NTD) 468.9 ± 327.7 vs Blindness NTD 822.2 ± 192.2, p = 0.0005) [91].

One randomised controlled trial showed that PwDM are less likely to undergo DRS when a co-payment is applied compared to the free services (OR 0.6, 95% CI 0.5–0.7) [67]. Those who had no health insurance (OR 2.5, 95% CI 1.7–3.7) were less likely to be compliant with screening [81]. Sheppler et al., (2014) mentioned that those who had an insurance coverage complied more with annual eye examination (OR 2.2, 95% CI 1.1–4.3, p = 0.02) [89]. Lian et al., (2013) showed that being in the pay groups was negatively associated with uptake of screening (OR 0.6, 95% CI 0.5–0.7) following random allocation of PwDM to screen for DR at a user fee (US $ 8) or for free [67]. The study done by Moss et al., (1995) showed that having a health insurance with eye examination covered (OR 3.3, 95% CI 2.2–5.1, p<0.0001) was associated with previous dilated eye examination [71].

### Family income

Two studies found that a higher family income was associated with having had a dilated eye examination (US $ >50,000 vs US $ <40,000, OR 1.9, 95% CI 1.3–2.9 [90] and US $ >35,000, OR 1.3, 95%CI 0.8–2.2) [98]. The study done by Paskin-Hall et al., (2013) showed that those who have a higher income ($35,000-$49,000 adjusted OR 1.3, 95% CI 1.1–1.5) had higher odds of undergoing DRS [78]. Another study done in South Korea by Rim et al., (2013) showed that those who were in the highest monthly income quintile (OR 1.4, 95% CI 1.1–1.8, p<0.01) had higher odds of undergoing screening [83].

### Gender

The odds of having had a dilated fundoscopy in the past year was high among women (OR 1.2, 95% CI 0.9–1.5) [90] and past eye care use decreased by being male (OR 0.5, 95% CI 0.3–0.8, p<0.01) [79]. However a study done in UK showed males had higher odds of attending screening following invitation (OR 1.4, 95% CI 1.1–1.7) [111]. Therefore, role of gender with regard to uptake of DRS could be context specific.

### Age

The PwDM > 70 years of age showed higher odds of having undergone dilated fundoscopy compared with those <40 years of age (OR 1.9, 95% CI 1.5–2.6) [90]. Most of the studies showed that older PwDM had higher odds of undergoing screening (age >65 years, OR 2.6, 95% CI 1.6–4.1)[98], (OR 1.02, p<0.001) [72].

It was observed that eye care services utilization with in a 12 month period, was lower in those who are younger (age 20–39 years, OR 0.1, 95% CI 0.01–0.70 p<0.05) [79]. Similarly, a study done in UK showed that younger age was associated with non-attendance (18–34 years, adjusted OR 1.4, 95% CI 1.1–1.7, 35–44 years, OR 1.4, 95% CI 1.2–1.7) [111].

### Level of education

Most of the studies mentioned the association between level of education and DRS uptake. For PwDM having more than high school education vs less than ninth grade education (OR 1.5, 95% CI 1.0–2.1) was associated with higher likelihood of having a dilated eye examination [90]. The reasons for non-adherence mentioned in another study was education less than high school (OR 1.5, 95% CI 1.1–2.1) [81]. It was observed that the odds of past eye care use dropped with the decrease in number of years of educational attainment (<10 years, OR 0.4, 95% CI 0.2–0.9, p<0.05) [79].

In addition, education up to high school or more was a predictor of knowledge that uncontrolled diabetes could cause eye disease (OR 2.4, 95%CI 1.5–4.0, P<0.05) [73]. Xiong et al., (2015) also showed that higher awareness of DR was seen among the more educated people (OR 1.8, 95% CI 0. 98–3.44, p = 0.0000) [54]. Islam et al., (2018) reported that having secondary or higher education was associated with improved DRS uptake (OR 11.8 (95% CI 4.02–34.7) [48].

### Disease factors associated with uptake of screening

Higher level of glycosylated haemoglobin was associated with non-compliance with screening (>9%, OR 1.7, 95% CI 1.1–2.6) [81].

### Diabetes / Eye care education

One study mentioned that those without DM education (OR 0.4, 95%CI 0.2–0.6) are less likely to undergo screening [50]. Hwang et al., (2015) in Canada showed that increased eye screening was associated with health professional discussing DM complications with PwDM (OR 2.0, 95% CI 1.3–3.2, p = 0.00) [62]. Persons having attended a DM education class (OR 1.5, 95% CI 1.2–1.9) had higher likelihood of having a dilated eye examination [90].

A study done in USA showed that eye care education (OR 1.6, 95% CI 1.2–2.1) was associated with receipt of dilated eye examination [98]. No formal DM education (OR 1.3, 95% CI 1.1–1.6) and less practical knowledge on DM (OR 1.6, 95% CI 1.2–2.1) were associated with non-adherence [88]. Those who had attended a DM education class had higher odds of having a dilated eye examination in the past year (OR 1.5, 95% CI 1.2–1.9) [90]. It is shown that when there has not been any education on DM, patients are less likely to visit an ophthalmologist on a regular basis (OR 0.39, 95% CI 0.24–0.65) [50].

### Personnel who conducted the last eye examination

The described reasons for non-adherence to DRS included, the type of personnel that conducted the last eye examination. It is shown that non-adherence was high when last examination had been conducted by non-ophthalmologist personnel (OR 4.3, 95% CI 2.3–6.2) [88].

### Duration of diabetes

The duration of DM was a predictor of having DR. Those who have had DM for a shorter duration (<5 years, OR 0.04, 95% CI 0.01–0.10) were less likely to be having DR [42]. One study mentioned that PwDM who were <5 years (OR 0.4, 95% CI 0.3–0.8) of duration after diagnosis are less likely to undergo screening [50].

Hwang et al., (2015) in Canada showed that duration of DM longer than 10 years (OR 1.5, 95% CI 1.04–2.25, p = 0.03)] was associated with increased eye screening [62]. Similarly Saadine et al., (2008) mentioned that when the DM duration was longer (>15 years, OR 1.9, 95%CI 1.4–2.6, p<0.0001) those PwDM were more likely to attend follow ups [84].

A factor positively correlating with eye care use was, time since diagnosis of DM (20 years, OR 2.7, 95% CI 1.2–5.9, p = 0.041) [79]. In contrast to other studies one research showed that when the duration of DM goes up, the likelihood of not attending screening also increases (5 to 9 years, OR 1.9, 95% CI 1.6–2.2), (>20 years, OR 3.4, 95% CI 2.7–4.2) [111].

### Type of diabetes treatment

Eye care use by PwDM was higher when the treatment is with oral antidiabetics and insulin (OR 2.8, 95% CI 1.1–7.4, p = 0.161) [79].

### Regularity of clinic visits

It was observed that the odds of using eye care in the past year decreased with those who are in the younger age categories (age 20–39 years, OR 0.09, 95% CI 0.01–0.70, p<0.05), and having lesser number of years in educational attainment (<10 years, OR 0.37, 95% CI 0.16–0.88, p<0.05) [79]. Further noncompliance was associated with those who had no routine physical examination > 1 year ago (OR 1.8, 95% CI 1.3–2.5) [81].

Mukamel et al., (2016) showed that patients who visit their primary care physicians more often (OR 1.3, 0.001<p<0.01) had higher probability of attending screening in the past 12 month period [72].

### Marital status

It was observed that past eye care use as being lower in those who were never married (OR 0.14, 95% CI 0.03–0.76, p<0.05) [79].

### Unemployment

Unemployment was inversely associated with eye care use (OR 0.5, 95% CI 0.2–1.1, p = 0.091) [79].

### Alcohol intake

Heavy alcohol consumption was inversely associated with eye care use (OR 0.3, 95% CI 0.1–0.7, p = 0.003) [79].

### Having other complications of diabetes

Factors inversely associated with eye care use was having diabetic foot disease (OR 0.4, 95% CI 0.2–0.9, p = 0.35) [79]. In contrast a study conducted by Bennet et al., (2018) found that having non-ocular complications of DM increased DRS attendance (OR 2.7, 95% CI 1.1 to 6.4) [99].

### Physician recommendation

Physician recommendation is a predictor of having regular eye examinations as mentioned in one study done in Ireland (OR 1.3, 95% CI 1.1–1.6) [108]. Van-EijK et al., (2011) showed that recommendation by the care provider was a strong incentive for undergoing DRS (OR 341, 95% CI 164–715) [93]. A similar association has been mentioned by the Wang et al., (2010) (OR 2.2, 95% CI 1.5–3.3, P<0.001) [53]. Being referred for eye examination was a strong predictor of high uptake in a study conducted in Kenya (OR 20.5, 95% CI 10.2–40.9, p < 0.001) [49].

### Having other eye diseases and visual impairment

Those who have other eye diseases (OR 1.2, 95% CI 1.1–1.6) and those who think that eye examinations are needed every 6 months (OR 1.2, 95% CI 1.1–1.4) showed higher odds of DRS uptake [108]. Study by Moss et al., (1995) showed that history of cataract (OR 2.9, 95%CI 1.9–4.4, p<0.0001) was associated with previous dilated eye examination [71]. Hwang et al., (2015) in Canada showed that increased eye screening was associated with having visual impairment (OR 2.6, 95% CI 1.7–3.9, p = 0.00) [62].

### Social deprivation

Even in HIC people living in deprived areas failed to attend DRS (OR 2.3, 95% CI 1.9–2.8) [66]. One study stated that people living in most deprived areas (OR 1.2, 95% CI 1.2–1.3) were more likely to not adhere with screening recommendations [77]. A study done in UK showed the factors associated with non-attendance following an invitation for screening in a sample of 31,484 diabetics in a DRS program. In this study social deprivation (adjusted OR 1.4, 95% CI 1.2–1.6, p<0.001) was associated with non-attendance [111].

Another study done in South Korea by Rim et al., (2013) showed that those who lived in urban areas (OR 1.5, 95% 1.2–1.8, p<0.01) and those in the highest monthly income quintile (OR 1.4, 95% CI 1.1–1.8, p<0.01) had higher odds of undergoing screening [83]. Scanlon et al., (2008) mentioned that with each increasing quintile of socioeconomic deprivation the probability of having been screened for DR decreased (OR 1.1, 95%CI 1.1–1.2, P<0.001) [85].

### Risk of development of DR among non-attendees

The relative risk of having DR was higher in non-attendees for screening, as shown in one study conducted in Yemen (Relative risk of having DR 1.5, 95% CI 1.2–2.2), (bilateral blindness 4.0, 95% CI 1.4–11.6) (low vision disability 2.4, 95% CI 1.8–3.5)[42].

Saadine et al., (2008) mentioned that those who have moderate or worse retinopathy (OR 2.2, 95%CI 1.6–2.9, p<0.0001) were more likely to attend follow ups [84].

## Discussion

We assessed the barriers and enablers to access and provision of DRS services in various country level income settings. This is the first systematic review to explore consumer, provider and health system barriers / enablers, and to understand these in the context of country income level. Knowing the barriers / enablers by country income setting is useful to identify and streamline interventions for the impeders in advance. Though the potential benefits to PwDM are widely known, attendance is at a sub-optimal level in DRS programs even in HIC settings [117]. DRS has been shown to be cost effective in terms of sight years preserved [118]. In most parts of the world DRS remains non-systematic. The findings from this narrative review will be useful to emphasise the barriers faced by consumers and providers in a DRS program. This will be helpful to explore the avenues for successful implementation of a DRS program in a country and how to conduct a program conveniently for both users and providers. We assumed that identification of secondary factors associated with uptake will be useful in efficiently continuing the programs and to identify the risk groups in advance.

We identified that knowledge and awareness among PwDM as the main barrier to access in all income settings. A recent review has also emphasised that lack of knowledge and awareness among PwDM as a major barrier to improve uptake [30]. Under the knowledge theme we identified many subthemes that would be useful for development of health educational interventions. Few such sub-themes are asymptomatic nature of DR and knowledge on frequency of DRS. This aspect has been described in a recent review as behavioural economics to improve access [119]. Therefore, we could assume that health educational interventions may improve uptake of services. However, the uptake of DRS services can be affected by various socio-economic factors as well, as observed in the review outcomes.

In our review, we identified that in the HIC most of the barriers to access were related to processes of DRS while in LIC and LMIC they were related to major system factors such as unavailability of services, lack of human resources and infrastructure. Most of the HIC settings provide population-based DRS using digital retinal imaging. Therefore, the barriers / enablers of HICs described in our review were attuned to the processes of DRS using imaging. However, most of the low-income settings still do not have systematic DRS and it is done as an opportunistic intervention only. Moreover, mode of DRS in low income settings were based on bio-microscopy / ophthalmoscopy. At the provider level in LIC and LMIC settings, lack of skilled human resources and lack of DRS infrastructure were the main barriers, while in UMIC and HIC it was lack of training and poor coordination between physicians / general practitioners and screeners. In addition, the LIC and LMIC ophthalmologists are overburdened with most prevalent blinding conditions such as cataract. This reflected in most of the studies as a barrier, stating the lack of and maldistribution of ophthalmologists. On the other hand, it has led to increased waiting time for PwDM, which hindered uptake of services.

Synthesis of existing evidence helped to narrow down barriers to identify modifiable themes. In general, knowledge appeared as the main modifiable barrier to access, from the user side. However, in a paid healthcare system, low income and financial constrains had been mentioned frequently. We identified financial barriers as a recurrent theme in the harvest plots. In addition, most frequently mentioned (i.e., frequency of studies with the theme) barrier by consumers was asymptomatic nature of DR as shown in harvest plot in Fig 2. Therefore, the need to undergo regular screening even without visual symptoms should be an aspect that should be emphasised. Complementary to these outcomes, the most common incentives mentioned in included studies were better knowledge on DR / DRS, higher level of education, presence of symptoms and higher level of income as shown in Fig 3. When considering the most frequently cited barriers by providers, deficiencies in educating users on DR / DRS, issues in accessibility when making appointments, and long waiting time at eye clinics emerged as the main barriers (Fig 4). The main enablers for providers were educating users on regular eye examination and providing better access for PwDM (Fig 5).

### Strengths and limitations

This review included 77 articles from diverse settings. We used a comprehensive approach to capture all possible articles on this review question. Inclusion of studies without restricting the study design allowed us to derive a wide range of themes. We used narrative synthesis of data due to high heterogeneity among the studies. Further we attempted to provide wide range of barrier and enabler themes by all income settings, incorporating both qualitative and descriptive quantitative studies, without restricting to any study design or income setting.

In order to maintain the homogeneity among the included studies, we divided the studies according to the income setting. Further we explored whether there were differences in barriers and enablers between different income settings.

A majority of the studies were focused on the perspectives of the users when describing the barriers. Almost none of the studies explored the perspectives of the policy makers or program planners. Therefore, this review lacks several aspects of stakeholder perspectives.

We did not look at the community level programs which may take place outside of a medical or eye care centre. We did not specifically assess the reach or availability of DRS programs, which could be an important component in access.

The included studies reflected the barriers in a cross section of time. All the studies used diagnosed PwDM at institutional level as their study samples. There were no studies that used long term sociological and ethnographic approaches to study barriers to access in their natural environment over time.

Many of the barriers or enablers identified in this review were peculiar to modality of screening in the local context. We used a reductionistic approach in this narrative synthesis without further synthesis of new themes. Another aspect is that the barriers or enablers were assessed in different health systems which may have different socio-cultural and economic back grounds. Therefore, we could not assess the interactions between each of the themes we derived. Though we simplified and de-contextualised the barriers themes, generalizability may depend on the context.

One of the limitations of this review is lack of eligible randomised controlled trials on the review question and primary outcomes were described as explained by the authors. Considering the paucity of systematic reviews under this topic, we found it is difficult to compare and comment in contrast on our findings.

### Implications and public health significance of the findings

The narrative synthesis by country income level supported by quantitative data would be helpful to identify potential strategies to overcome barriers in each setting. We observed that most important factor to define barriers is the setting. Therefore, we recommend carrying out an assessment of barriers and enablers in each context before making recommendations for a DRS program.

Diabetic retinopathy screening program implementation involves a high capital expenditure. There will be a high level of financial risk when implementing a program for the first time. By knowing the potential barriers, the risks can be minimised, and access can be improved by implementing interventions to overcome potential barriers.

The outcomes of the current review will be useful to identify the modifiable barriers which could be further explored in a local context before implementing costly DRS programs and interventions. Assessment of user and provider perspectives together enables the identification and subsequent catering to needs from the demand side as well as the supply side of DRS.

The results of this review show that there are modifiable barriers such as lack of knowledge on DRS among the PwDM which could be addressed in the development of health promotional strategies.

This review highlights the gaps in evidence on this topic in LIC and LMIC. Further there was limited evidence on system factors and perspectives of stakeholders.

## Conclusion

The evidence in this review clearly suggests that the barriers and enablers are different in each income setting. The most consistent barrier across different income settings was lack of knowledge and awareness on DR and DRS among the users. In providers point of view, lack of skilled human resources and screening infrastructure was the main barrier. Knowing the modifiable barriers in a specific context would be helpful to identify the risk groups early and to improve DRS uptake among institutional PwDM. A main recommendation of this review is to carry out an assessment of barriers and enablers in each context before implementing a DRS program. The consumer-based health educational interventions and provider-based skills and DRS infrastructure development would improve the access to DRS especially in low income settings.

## Supporting information

S1 Table PRISMA check list. (DOC)

S2 Table Search strategy of barriers to access systematic review. (DOCX)

S3 Table Methodological quality and applicability assessment of the included studies. (DOCX)

S4 Table Themes tables by country income setting. (DOCX)

S5 Table Quantitative Data Synthesis—Factors associated with DR screening uptake and regular follow up. (DOCX)

S6 Table Participants’ characteristics of included articles. (DOCX)
