# Supplementary material for: Systematic review on barriers and enablers for access to diabetic retinopathy screening services in different income settings
Source: PLoS One. 2019 Apr 23;14(4):e0198979. doi: 10.1371/journal.pone.0198979 (PMC6478270; doi:10.1371/journal.pone.0198979)
Supplement: S2 Table — (DOCX) [file pone.0198979.s002.docx]

**S2 Table****. Search strategy of barriers to access systematic review**

| **Medline / Ovid** | **EMBASE / Ovid** | **Cochrane** |
| --- | --- | --- |
| 1.exp Patient Acceptance of health care/  2.exp Attitude to health/  3.exp health behavior/  4.(Uptake or barrier$ or attend$ or accept$ or adhere$ or participate or facilitat$ or enable$).tw.  5.(motivat$ or staisf$ or takeup$ or consent$ or promot$).tw.  6.(complie$ or comply or compliance$ or noncompliance$ or non compliance$).tw.  7.(encourag$ discourage$ or reluctan$ or nonrespon$ or non respon$ or refuse$).tw.  8.(non-attend$ or non attend$ or dropout or drop out or apath$).tw.  9.Health Education/  10.exp Patient Education as Topic/  11.exp Health Promotion/  12.(educat$ adj2 (information or material or leaflet)).tw.  13.Socioeconomic Factors/  14.exp Poverty/  15.Social Class/  16.((school or education$) adj3 (status or level or attain$ or achieve$)).tw.  17.Uncompensated Care/  18.Reimbursement Mechanisms/  29.Reimbursement, Incentive/  20.(financial or pay or payment or copayment or paid or fee or fees or monetary or incentiv$).tw.  21.Healthcare Disparities/  22.Health Status Disparities/  23.exp Medically Underserved Area/  24.Rural Population/  25.Urban Population/  26.exp Ethnic Groups/  27.Minority Groups/  28.Vulnerable Populations/  29.((health$ or social$ or racial$ or ethnic$) adj5 (inequalit$ or inequit$ or disparit$ or equit$ or disadvantage$ or depriv$)).tw.  30.(disadvant$ or marginali$ or underserved or under served or impoverish$ or minorit$ or racial$ or ethnic$).tw.  31.exp culture/  32.sex factors/  33. ((gender or women$) adj4 (inequalit$ or inequit$ or disparit$ or equit$ or disadvantage$)).tw.  (34 –error)  35. 1 or 2 or 3 or 4 or 5 or 6 or 7 or 8 or 9 or 10 or 11 or 12 or 13 or 14 or 15 or 16 or 17 or 18 or 19 or 20 or 21 or 22 or 23 or 24 or 25 or 26 or 27 or 28 or 29 or 30 or 31 or 32 or 33  36 .exp Diabetic Retinopathy/  37.exp Diabetes Complications/  38.((diabet$ or proliferative non-proliferative) adj4 retinopath$).tw.  39 .(diabet$ adj4 (eye$ or vision or visual$)).tw.  40 .(retinopath$ adj3 (sight$ or vision or visual$)).tw.  41 .(DR adj3 (sight or vision or visual$)).tw.  42 or/36-41  43 exp mass screening/  44 .exp vision tests/  45 .exp telemedicine/  46 .exp Photography/  47 exp ophthalmoscopes/  48 .exp ophthalmoscopy/  49. (ophthalmoscop$ or fundoscop$ or funduscop$).ti.  50 .((photo$ or imag$) adj3 fundus).tw  52 .((mydiatric or digital or retina$ or funduc or stereoscopic) adj3 camera).tw.  53 .((mydiatric or digital or retina$ or fundus or stereoscopic) adj3 imag$).tw.  54 .Screen$.tw.  55 .((eye$ or retina$ or ophthalm$) adj4 exam$).tw.  56 .((eye$ or vision or ophthalmic) adj4 test$).tw.  57 .((eye$ or retina$ or ophthalm$) adj4 visit$).tw.  58 .Office visits/  59 .(telemedicine$ or telemonitor$ or telescreen$).tw.  60 43 or 44 or 45 or 46 or 47 or 48 or 49 or 50 or 51 or 52 or 53 or 54 or 55 or 56 or 57 or 58 or 59  61 35 and 42 and 60 | 1. exp randomized controlled trial/  2. exp randomization/  3. exp double blind procedure/  4. exp single blind procedure/  5. or/1-4  6. (animal or animal experiment).sh.  7. human.sh.  8. 6 and 7  9. 6 not 8  10. 5 not 9  11. exp clinical trial/  12. (clin$ adj3 trial$).tw.  13. random$.tw.  14. exp placebo/  15. placebo$.tw.  16. ((singl$ or doubl$ or trebl$ or tripl$) adj3 (blind$ or mask$)).tw.  17. exp experimental design/  18. exp crossover procedure/  19. exp control group/  20. exp latin square design/  21. or/11-20  22. 21 not 9  23. 22 not 10  24. exp comparative study/  25. exp evaluation/  26. exp prospective study/  27. (control$ or prospectiv$ or volunteer$).tw.  28. or/24-27  29. 28 not 9  30. 29 not (10 or 22)  31. 10 or 23 or 30  32. "randomized controlled trial (topic)"/  33. 31 or 32  34. exp diabetes mellitus/  35. exp diabetic retinopathy/  36. ((diabet$ or proliferative or non-proliferative) adj4 retinopath$).tw.  37. diabetic retinopathy.kw.  38. (diabet$ adj3 (eye$ or vision or visual$ or sight$)).tw.  39. (retinopath$ adj3 (eye$ or vision or visual$ or sight$)).tw.  40. (DR adj3 (eye$ or vision or visual$ or sight$)).tw.  41. or/34-40  42. exp Screening/  43. exp Vision Test/  44. Eye Examination/  45. Telemedicine/  46. Photography/  47. Eye Photography/  48. Ophthalmoscopy/  49. (ophthalmoscop$ or fundoscop$ or funduscop$).ti.  50. ((exam$ or photo$ or imag$) adj3 fundus).tw.  51. (photography or retinography).tw.  52. ((mydriatic or digital or retina$ or fundus or steroscopic) adj3 camera).tw.  53. ((mydriatic or digital or retina$ or fundus or steroscopic) adj3 imag$).tw.  54. screen$.tw.  55. ((eye$ or retina$ or ophthalm$) adj4 exam$).tw.  56. ((eye or vision or retinopathy or ophthalmic) adj4 test$).tw.  57. ((eye$ or retina$ or ophthalm$) adj4 visit$).tw.  58. (telemedicine$ or telemonitor$ or telescreen$ or telehealth or teleophthalmology).tw.  59. or/42-58  60. Health Care Quality/  61. Quality Improvement/  62. Health Care Delivery/  63. Integrated Health Care System/  64. service delivery.tw.  65. decision making.tw.  66. (consensus adj3 (process$ or discuss)).tw.  67. stakeholder$.tw.  68. Quality Control/  69. Total Quality Management/  70. quality assurance.tw.  71. (quality adj2 improv$).tw.  72. total quality.tw.  73. continuous quality.tw.  74. quality management.tw.  75. (organisation$ adj3 cultur$).tw.  76. disease management/  77. program evaluation/  78. ((provider$ or program$) adj3 (monitor$ or evaluate$ or modif$ or practice)).tw.  79. (implement$ adj3 (improve$ or change$ or effort$ or issue$ or impede$ or glossary or tool$ or innovation$ or outcome$ or driv$ or examin$ or reexamin$ or scale$ or strateg$ or advis$ or expert$)).tw.  80. (need$ adj3 assess$).tw.  81. ((education$ or learn$) adj5 (continu$ or material$ or meeting or collaborat$)).tw.  82. Medical audit/  83. (audit or feedback or compliance or adherence or training or innovation).ti.  84. (guideline$ adj3 (clinical or practice or implement$ or promot$)).tw.  85. (outreach adj2 (service$ or visit$)).tw.  86. (intervention$ adj3 (no or usual or routine or target$ or tailor$ or mediat$)).tw.  87. usual care.tw.  88. reminder system/  89. remind$.tw.  90. (improve$ adj3 (attend$ or visit$ or intervention$ or adhere$)).tw.  91. (increas$ adj3 (attend$ or visit$ or intervention$ or adhere$)).tw.  92. (appointment$ adj3 (miss$ or fail$ or remind$ or follow up)).tw.  93. telephone/  94. telephone.tw.  95. Mobile Phone/  96. Mobile Application/  97. Teleconsultation/  98. (m-health or e-health or g-health or u-health).tw.  99. (phone$ adj1 (smart or cell)).tw.  100. (smartphone$ or cellphone$).tw.  101. (hand adj1 held device$).tw.  102. (mobile adj2 (health or healthcare or phone$ or device$ or monitor$ or comput$ or app or apps or application)).tw.  103. Internet/  104. Social Network/  105. (email$ or text$ or message$).tw.  106. (letter or mail or mailed or print$ or brochure$ or newsletter$).tw.  107. Primary Health Care/  108. General Practitioner/  109. Primary Prevention/  110. Preventive Health Service/  111. Community Care/  112. Community Health Nursing/  113. exp Transcultural Care/  114. Rural Health Care/  115. Ophthalmologist/  116. (Ophthalmologist$ or Optometrist$ or Optician$ or Orthopist$ or Refractionists).tw.  117. ((Ophthalmic or eye) adj3 (surgeon$ or nurse$ or technician$ or officer$ or assistant$ or staff$)).tw.  118. Clinical Practice/  119. Professional Practice/  120. Continuing Education/  121. (professional adj3 (practice or develop$ or educat)).tw.  122. Nurse/  123. Nursing Discipline/  124. Nurse Attitude/  125. Nursing Education/  126. (nurse or nurses).tw.  127. pharmacist/  128. pharmacist$.tw.  129. ((role or roles) adj3 expan$).tw.  130. (task$ adj3 shift$).tw.  131. Electronic Medical Record/  132. Information System/  133. Data Base/  134. Computer System/  135. Hospital Information System/  136. ((health or healthcare) adj4 (record or management system$)).tw.  137. (decision adj5 support).ti.  138. cost benefit analysis/  139. cost effectiveness analysis/  140. cost of illness/  141. cost control/  142. economic aspect/  143. financial management/  144. health care cost/  145. health care financing/  146. health economics/  147. hospital cost/  148. (fiscal or financial or finance or funding).tw.  149. cost minimization analysis/  150. (cost adj estimate$).mp.  151. (cost adj variable$).mp.  152. (unit adj cost$).mp.  153. (economic$ or pharmacoeconomic$ or price$ or pricing).tw.  154. exp Reimbursement/  155. (financial or economic or pay or payment or copayment or paid or fee or fees or monetary or money or cash or incentiv$ or disincentiv$).tw.  156. (insurance adj3 (health$ or scheme$)).tw.  157. or/60-156  158. exp Patient Attitude/  159. exp Health Behaviour/  160. (barrier$ or obstacle$ or facilitat$ or enable$).tw.  161. (uptake or takeup or attend$ or accept$ or adhere$ or attitude$ or participat$ or facilitat$ or utilisat$ or utilizat$).tw.  162. (complie$ or comply or compliance$ or noncompliance$ or non compliance$).tw.  163. (encourag$ or discourage$ or reluctan$ or nonrespon$ or non respon$ or refuse$).tw.  164. (non-attend$ or non attend$ or dropout or drop out or apath$).tw.  165. Health Education/  166. exp Patient Education/  167. Diabetes Education/  168. Help Seeking Behavior/  169. Patient Participation/  170. Patient Decision Making/  171. exp Health Promotion/  172. (health adj2 (promotion$ or knowledge or belief$)).tw.  173. (educat$ adj2 (intervention$ or information or material or leaflet)).tw.  174. exp Socioeconomics/  175. Income/  176. Social Class/  177. Social Status/  178. Educational Status/  179. ((school or education$) adj3 (status or level$ or attain$ or achieve$)).tw.  180. Employment/  181. Health Care Disparity/  182. Health Disparity/  183. Rural Population/  184. Rural Area/  185. Urban Population/  186. Urban Area/  187. exp Ethnic Group/  188. Ethnicity/  189. Race Difference/  190. Minority Groups/  191. Vulnerable Populations/  192. ((health$ or social$ or racial$ or ethnic$) adj5 (inequalit$ or inequit$ or disparit$ or equit$ or disadvantage$ or depriv$)).tw.  193. (disadvant$ or marginali$ or underserved or under served or impoverish$ or minorit$ or racial$ or ethnic$).tw.  194. or/158-193  195. 157 or 194  196. 33 and 41 and 59 and 195  197. (ranibizumab or bevacizumab or avastin or aflibercept or photocoagulation or coronary or cardiovascular).ti.  198. (blood glucose or blood pressure).ti.  199. (macula$ adj2 (oedema or edema)).ti.  200. (cataract or intraocular or glaucoma).ti.  201. macula$ degeneration.ti.  202. nerve fiber layer.ti.  203. or/197-202  204. 196 not 203 | #1 MeSH descriptor: [Diabetes Mellitus] explode all trees  #2 MeSH descriptor: [Diabetes Complications] explode all trees  #3 MeSH descriptor: [Diabetic Retinopathy] explode all trees  #4 (diabet* or proliferative or non-proliferative) near/4 retinopath*  #5 diabet* near/3 (eye* or vision or visual* or sight*)  #6 retinopath* near/3 (eye* or vision or visual* or sight*)  #7 DR near/3 (eye* or vision or visual* or sight*)  #8 #1 or #2 or #3 or #4 or #5 or #6 or #7  #9 MeSH descriptor: [Mass Screening] explode all trees  #10 MeSH descriptor: [Vision Tests] explode all trees  #11 MeSH descriptor: [Telemedicine] explode all trees  #12 MeSH descriptor: [Photography] explode all trees  #13 MeSH descriptor: [Ophthalmoscopes] explode all trees  #14 MeSH descriptor: [Ophthalmoscopy] explode all trees  #15 ophthalmoscop* or fundoscop* or funduscop*:ti  #16 (exam* or photo* or imag*) near/3 fundus  #17 photography or retinography  #18 (mydriatic or digital or retina* or fundus or steroscopic) near/3 camera*  #19 (mydriatic or digital or retina* or fundus or steroscopic) near/3 imag*  #20 screen$.tw.  #21 (eye* or retina* or ophthalm*) near/4 exam*  #22 (eye* or vision or retinopathy or ophthalmic) near/4 test*  #23 (eye* or retina* or ophthalm*) near/4 visit*  #24 MeSH descriptor: [Office Visits] this term only  #25 (telemedicine* or telemonitor* or telescreen* or telehealth or teleophthalmology)  #26 #9 or #10 or #11 or #12 or #13 or #14 or #15 or #16 or #17 or #18 or #19 or #20 or #21 or #22 or #23 or #24 or #25  #27 MeSH descriptor: [Quality of Health Care] explode all trees  #28 MeSH descriptor: [Quality of Health Care] this term only  #29 MeSH descriptor: [Quality Improvement] this term only  #30 MeSH descriptor: [Delivery of Health Care] this term only  #31 MeSH descriptor: [Delivery of Health Care, Integrated] this term only  #32 service delivery  #33 decision making  #34 consensus near/3 (process* or discuss)  #35 stakeholder*  #36 MeSH descriptor: [Quality Control] this term only  #37 MeSH descriptor: [Total Quality Management] this term only  #38 MeSH descriptor: [Quality Indicators, Health Care] this term only  #39 MeSH descriptor: [Quality Assurance, Health Care] this term only  #40 quality assurance  #41 quality near/2 improv*  #42 total quality  #43 continuous quality  #44 quality management  #45 (organisation* near/3 cultur*)  #46 MeSH descriptor: [Disease Management] this term only  #47 MeSH descriptor: [Program Evaluation] this term only  #48 (provider* or program*) near/3 (monitor* or evaluate* or modif* or practice)  #49 implement* near/3 (improve* or change* or effort* or issue* or impede* or glossary or tool* or innovation* or outcome* or driv* or examin* or reexamin* or scale* or strateg* or advis* or expert*)  #50 needs near/3 assess*  #51 (education* or learn*) near/5 (continu* or material* or meeting or collaborat*)  #52 MeSH descriptor: [Medical Audit] explode all trees  #53 audit or feedback or compliance or adherence or training or innovation:ti  #54 guideline* near/3 (clinical or practice or implement* or promot*)  #55 MeSH descriptor: [Health Services Accessibility] explode all trees  #56 outreach near/2 (service$ or visit*)  #57 intervention* near/3 (no or usual or routine or target* or tailor* or mediat*)  #58 usual care  #59 #27 or #28 or #29 or #30 or #31 or #32 or #33 or #34 or #35 or #36 or #37 or #38 or #39 or #40 or #41 or #42 or #43 or #44 or #45 or #46 or #47 or #48 or #49 or #50 or #51 or #52 or #53 or #54 or #55 or #56 or #57 or #58  #60 MeSH descriptor: [Reminder Systems] explode all trees  #61 remind*  #62 improve* near/3 (attend* or visit* or intervention* or adhere*)  #63 increas* near/3 (attend* or visit* or intervention* or adhere*)  #64 appointment* near/3 (miss* or fail* or remind* or follow up)  #65 MeSH descriptor: [Telephone] this term only  #66 telephone*  #67 MeSH descriptor: [Cell Phones] this term only  #68 MeSH descriptor: [Mobile Applications] this term only  #69 MeSH descriptor: [Remote Consultation] this term only  #70 m-health or e-health or g-health or u-health  #71 phone* near/1 (smart or cell)  #72 smartphone* or cellphone*  #73 hand held device*  #74 mobile near/2 (health or healthcare or phone* or device* or monitor* or comput* or app or apps or application)  #75 MeSH descriptor: [Internet] this term only  #76 MeSH descriptor: [Social Networking] this term only  #77 email* or text* or message*  #78 letter or mail or mailed or print* or brochure* or newsletter*  #79 #60 or #61 or #62 or #63 or #64 or #65 or #66 or #67 or #68 or #69 or #70 or #71 or #72 or #73 or #74 or #75 or #76 or #77 or #78  #80 MeSH descriptor: [Primary Health Care] this term only  #81 MeSH descriptor: [General Practitioners] this term only  #82 MeSH descriptor: [Physicians, Family] this term only  #83 MeSH descriptor: [Physicians, Primary Care] this term only  #84 MeSH descriptor: [Primary Prevention] this term only  #85 MeSH descriptor: [Preventive Health Services] this term only  #86 MeSH descriptor: [Community Health Services] this term only  #87 MeSH descriptor: [Nurses, Community Health] this term only  #88 MeSH descriptor: [Health Services, Indigenous] this term only  #89 MeSH descriptor: [Rural Health Services] explode all trees  #90 MeSH descriptor: [Mobile Health Units] this term only  #91 Ophthalmologist* or Optometrist* or Optician* or Orthopist* or Refractionists  #92 (Ophthalmic or eye) near/3 (surgeon* or nurse* or technician* or officer* or assistant* or staff*)  #93 MeSH descriptor: [Physician's Practice Patterns] this term only  #94 MeSH descriptor: [Professional Practice] this term only  #95 MeSH descriptor: [Education, Medical, Continuing] this term only  #96 MeSH descriptor: [Nurses] explode all trees  #97 MeSH descriptor: [Specialties, Nursing] this term only  #98 MeSH descriptor: [Nurse's Role] this term only  #99 MeSH descriptor: [Education, Nursing, Continuing] this term only  #100 nurse or nurses  #101 MeSH descriptor: [Pharmacists] this term only  #102 pharmacist*  #103 (role or roles) near/3 expan*  #104 task* near/3 shift*  #105 MeSH descriptor: [Medical Records Systems, Computerized] explode all trees  #106 MeSH descriptor: [Management Information Systems] this term only  #107 MeSH descriptor: [Database Management Systems] this term only  #108 MeSH descriptor: [Computer Systems] this term only  #109 MeSH descriptor: [Point-of-Care Systems] this term only  #110 MeSH descriptor: [Hospital Information Systems] this term only  #111 (health or healthcare) near/4 (record or management system*)  #112 (decision near/5 support) .ti.  #113 #80 or #81 or #82 or #83 or #84 or #85 or #86 or #87 or #88 or #89 or #90 or #91 or #92 or #93 or #94 or #95 or #96 or #97 or #98 or #99 or #100 or #101 or #102 or #103 or #104 or #105 or #106 or #107 or #108 or #109 or #110 or #111 or #112  #114 MeSH descriptor: [Economics] this term only  #115 MeSH descriptor: [Costs and Cost Analysis] this term only  #116 MeSH descriptor: [Cost Allocation] this term only  #117 MeSH descriptor: [Cost-Benefit Analysis] this term only  #118 MeSH descriptor: [Cost Control] this term only  #119 MeSH descriptor: [Cost Savings] this term only  #120 MeSH descriptor: [Cost of Illness] explode all trees  #121 MeSH descriptor: [Cost Sharing] this term only  #122 MeSH descriptor: [Deductibles and Coinsurance] this term only  #123 MeSH descriptor: [Medical Savings Accounts] this term only  #124 MeSH descriptor: [Health Care Costs] this term only  #125 MeSH descriptor: [Direct Service Costs] this term only  #126 MeSH descriptor: [Drug Costs] this term only  #127 MeSH descriptor: [Employer Health Costs] this term only  #128 MeSH descriptor: [Hospital Costs] this term only  #129 MeSH descriptor: [Health Expenditures] this term only  #130 MeSH descriptor: [Capital Expenditures] this term only  #131 MeSH descriptor: [Economics, Hospital] explode all trees  #132 MeSH descriptor: [Economics, Medical] explode all trees  #133 MeSH descriptor: [Economics, Nursing] this term only  #134 MeSH descriptor: [Economics, Pharmaceutical] this term only  #135 MeSH descriptor: [Fees and Charges] explode all trees  #136 MeSH descriptor: [Budgets] explode all trees  #137 low* near/2 cost*  #138 high* near/2 cost*  #139 (health care or healthcare) near/2 cost*  #140 fiscal or funding or financial or finance  #141 cost near/2 estimate*  #142 cost near/2 variable*  #143 unit near/2 cost*  #144 economic* or pharmacoeconomic* or price* or pricing  #145 MeSH descriptor: [Uncompensated Care] this term only  #146 MeSH descriptor: [Reimbursement Mechanisms] this term only  #147 MeSH descriptor: [Reimbursement, Incentive] this term only  #148 insurance near/3 (health or scheme*)  #149 financial or economic or pay or payment or copayment or paid or fee or fees or monetary or money or cash or incentiv* or disincentiv*  #150 #114 or #115 or #116 or #117 or #118 or #119 or #120 or #121 or #122 or #123 or #124 or #125 or #126 or #127 or #128 or #129 or #130 or #131 or #132 or #133 or #134 or #135 or #136 or #137 or #138 or #139 or #140 or #141 or #142 or #143 or #144 or #145 or #146 or #147 or #148 or #149  #151 #59 or #79 or #113 or #150  #152 MeSH descriptor: [Patient Acceptance of Health Care] explode all trees  #153 MeSH descriptor: [Attitude to Health] explode all trees  #154 MeSH descriptor: [Health Behavior] explode all trees  #155 barrier* or obstacle* or facilitat* or enable*  #156 uptake or takeup or attend* or accept* or adhere* or attitude* or participat* or facilitat* or utilisat* or utilizat*  #157 complie* or comply or compliance* or noncompliance* or non compliance*  #158 encourag* or discourage* or reluctan* or nonrespon* or non respon* or refuse* or refusal  #159 non-attend* or non attend* or dropout or drop out or apath*  #160 MeSH descriptor: [Health Education] this term only  #161 MeSH descriptor: [Patient Education as Topic] explode all trees  #162 MeSH descriptor: [Health Promotion] explode all trees  #163 health near/2 (promotion* or knowledge or belief*)  #164 educat* near/2 (intervention* or information or material or leaflet)  #165 MeSH descriptor: [Socioeconomic Factors] this term only  #166 MeSH descriptor: [Poverty] explode all trees  #167 MeSH descriptor: [Social Class] this term only  #168 MeSH descriptor: [Educational Status] this term only  #169 (school or education*) near/3 (status or level* or attain* or achieve*)  #170 MeSH descriptor: [Employment] this term only  #171 MeSH descriptor: [Healthcare Disparities] this term only  #172 MeSH descriptor: [Health Status Disparities] this term only  #173 MeSH descriptor: [Medically Underserved Area] explode all trees  #174 MeSH descriptor: [Rural Population] this term only  #175 MeSH descriptor: [Urban Population] this term only  #176 MeSH descriptor: [Ethnic Groups] explode all trees  #177 MeSH descriptor: [Minority Groups] this term only  #178 MeSH descriptor: [Vulnerable Populations] this term only  #179 (health* or social* or racial* or ethnic*) near/5 (inequalit* or inequit* or disparit* or equit* or disadvantage* or depriv*)  #180 disadvant* or marginali* or underserved or under served or impoverish* or minorit* or racial* or ethnic*  #181 #152 or #153 or #154 or #155 or #156 or #157 or #158 or #159 or #160 or #161 or #162 or #163 or #164 or #165 or #166 or #167 or #168 or #169 or #170 or #171 or #172 or #173 or #174 or #175 or #176 or #177 or #178 or #179 or #180  #182 #151 or #181  #183 #8 and #26 and #182  #184 (ranibizumab or bevacizumab or avastin or aflibercept or photocoagulation or coronary or cardiovascular):ti  #185 blood glucose or blood pressure:ti  #186 macula* near/2 (oedema or edema):ti  #187 #184 or #185 or #186  #188 #183 not #187 |
