# Supplementary material for: Systematic review on barriers and enablers for access to diabetic retinopathy screening services in different income settings
Source: PLoS One. 2019 Apr 23;14(4):e0198979. doi: 10.1371/journal.pone.0198979 (PMC6478270; doi:10.1371/journal.pone.0198979)
Supplement: S3 Table — (DOCX) [file pone.0198979.s003.docx]

**S3 Tables. Methodological quality and applicability assessment of the included studies**

**Table 1.** Methodological Quality Assessment of Cross-Sectional Studies

|  | *1.Research question or objectives clearly stated* | *2.Study population clearly specified and defined* | *3.Participation rate of eligible persons at least 50%* | *4.All the subjects recruited from similar populations in the same time period* | *5.Inclusion and exclusion criteria prespecified and applied uniformly* | *6.Sample size justification, power description, or variance and effect estimates provided* | *7.Exposure(s) of interest measured prior to the outcome(s) being measured* | *8.Timeframe sufficient to see an association between exposure and outcome* | *9.For exposures that can vary in amount or level, the study examined different levels of the exposure related to outcome* | *10.Exposure measures clearly defined, valid, reliable, and implemented consistently across all study participants* | *11.Exposure(s) assessed more than once over time* | *12.Outcome measures clearly defined, valid, reliable, and implemented consistently across all study participants* | *13.Outcome assessors blinded to the exposure status of participants* | *14.Loss to follow-up after baseline 20% or less* | *15.Key potential confounding variables measured and adjusted statistically* |
| --- | --- | --- | --- | --- | --- | --- | --- | --- | --- | --- | --- | --- | --- | --- | --- |
| **1.Abdulsalam S et al (2018) (Nigeria)** | Yes | Yes | Yes | Yes | Yes | Yes | N/A | N/A | N/A | N/A | N/A | Yes | N/A | N/A | N/A |
| **2.Adriono G et al (2011) (Indonesia)** | Yes | Yes | Yes | Yes | Yes | No | No | No | Yes | Yes | No | No | No | N/A | Yes |
| **3.Agarwal S et al (2005) (India)** | Yes | Yes | N/A | Yes | Not reported | N/A | N/A | N/A | N/A | N/A | N/A | Yes | N/A | N/A | N/A |
| **4.Anderson S et al (2003) (UK)** | Yes | Yes | Yes | Yes | Yes | N/A | N/A | N/A | N/A | N/A | N/A | No | N/A | N/A | N/A |
| **5.Baumeister SE et al (2015) (Germany)** | No | Yes | N/A | Yes | Yes | N/A | N/A | N/A | Yes | No | N/A | No | Not reported | N/A | Yes |
| **6.Bennet GH et al (2018) (Ireland)** | No | Yes | Yes | Yes | Not reported | Not reported | N/A | N/A | N/A | N/A | N/A | Yes | N/A | No | No |
| **7.Brechner RJ et al (1993) (USA)** | Yes | Yes | Yes | Yes | Yes | No | N/A | N/A | Yes | No | N/A | No | N/A | N/A | Yes |
| **8.Cetin EN et al (2013) (Turkey)** | Yes | Yes | Yes | Yes | Yes | No | N/A | N/A | Yes | Yes | N/A | No | N/A | N/A | Yes |
| **9.Creuzot GC et al (2014)** | Yes | Yes | Yes | Yes | Yes | No | N/A | N/A | No | Yes | N/A | No | No | No | Yes |
| **10.Dervan E et al (2008) (Ireland)** | Yes | Yes | Yes | Yes | Yes | No | N/A | N/A | Yes | Yes | N/A | No | N/A | N/A | Yes |
| **11.Eiser JR et al (2001) (UK)** | Yes | Yes | Yes | Yes | Yes | No | N/A | N/A | Yes | No | N/A | No | N/A | N/A | Yes |
| **12.Foreman J et al (2017) (Australia)** | Yes | Yes | Yes | Yes | Yes | No | N/A | N/A | N/A | N/A | N/A | Yes | N/A | N/A | N/A |
| **13.Gillibrand WP et al (2000) (UK)** | Yes | Yes | Yes | Yes | Yes | No | N/A | N/A | N/A | N/A | N/A | No | N/A | N/A | No |
| **14.Gulliford MC et al (2010) UK** | Yes | Yes | N/A | Yes | Yes | No | N/A | N/A | Yes | No | N/A | Yes | N/A | N/A | Yes |
| **15.Harvey JN et al (2006) (UK)** | Yes | Yes | Yes | Yes | Yes | No | N/A | N/A | N/A | Yes | N/A | Yes | Not reported | N/A | No |
| **16.Huang OS et al (2009) (Singapore)** | Yes | Yes | Yes | Yes | Yes | No | N/A | N/A | Yes | Yes | N/A | Yes | Yes | N/A | Yes |
| **17.Hwang J et al (2015) (Canada)** | Yes | Yes | Yes | Yes | Yes | No | N/A | N/A | Yes | Yes | N/A | No | N/A | N/A | Yes |
| **18.Islam FMA et al (2018) (Bangladesh)** | Yes | Yes | Not Reported | Yes | Yes | No | Yes | Yes | Yes | Yes | No | Yes | N/A | N/A | Yes |
| **19.Katibeh M et al (2017) (Iran) 2^nd^ Article** | Yes | Yes | Yes | Yes | Yes | No | N/A | N/A | Yes | Yes | N/A | Yes | N/A | N/A | Yes |
| **20.Khandekar R et al (2008) (Oman)** | Yes | Yes | Yes | Yes | Yes | Yes | N/A | N/A | N/A | N/A | N/A | Yes | N/A | N/A | N/A |
| **21.Kurji K et al (2013)** | Yes | Yes | No | Yes | Yes | No | N/A | N/A | N/A | N/A | N/A | Yes | N/A | No | No |
| **22.Lee PP et al (1998)** | Yes | Yes | Yes | Yes | No | No | N/A | N/A | N/A | Yes | N/A | Yes | N/A | Yes | Yes |
| **23.Leese GP et al (2008) (UK)** | Yes | Yes | N/A | Yes | Yes | N/A | N/A | N/A | Yes | Yes | N/A | Yes | N/A | N/A | Yes |
| **24.Lian J et al (2018) (Hong Kong)** | Yes | Yes | Yes | Yes | Yes | No | Yes | N/A | Yes | Yes | N/A | Yes | N/A | N/A | Yes |
| **25.Moreton RBR et al (2017) (UK)** | Yes | Yes | N/A | Yes | Yes | N/A | N/A | N/A | Yes | Yes | N/A | Yes | N/A | N/A | Yes |
| **26.Moss SE et al (1995) (USA)** | Yes | Yes | No | Yes | Yes | No | N/A | N/A | Yes | Yes | N/A | Yes | N/A | N/A | Yes |
| **27.Muecke JS et al (2008) (Myanmar)** | Yes | No | No | Yes | No | No | N/A | N/A | Yes | Not Reported | N/A | Not Reported | N/A | N/A | Not Reported |
| **28.Mukamel DB et al (1999) (USA)** | Yes | Yes | N/A | Yes | Yes | No | N/A | N/A | Yes | Yes | N/A | Yes | N/A | N/A | Yes |
| **29.Mumba M et al (2007)** | Yes | Yes | Yes | Yes | No | No | N/A | N/A | N/A | N/A | N/A | Yes | N/A | Yes | Yes |
| **30.Munoz B et al (2008) (USA)** | Yes | Yes | Yes | Yes | Yes | No | N/A | N/A | Yes | Yes | N/A | Yes | N/A | N/A | Yes |
| **31.Murgatroyd H et al (2006) (UK)** | Yes | No | Not reported | Yes | Not reported | No | N/A | N/A | N/A | Yes | N/A | Not Reported | N/A | N/A | No |
| **32.Mwangi N et al (2017) (Kenya)** | Yes | Yes | Yes | Yes | Yes | Yes | N/A | N/A | Yes | Yes | N/A | Yes | N/A | N/A | Yes |
| **33.Namperumalsamy P et al (2004) (India)** | Yes | Yes | Yes | Yes | Yes | No | N/A | N/A | N/A | N/A | N/A | Yes | N/A | N/A | No |
| **34.Newcomb PA et al (1990) (USA)** | Yes | Yes | Yes | Yes | Yes | No | Yes | Yes | Yes | Yes | Yes | No | Not reported | Yes | Yes |
| **35.Newcomb PA et al (1992)** | Yes | Yes | Yes | Yes | Yes | Yes | Yes | No | No | Yes | No | Yes | No | Yes | Yes |
| **36.Onakpoya OH et al (2010) (Nigeria)** | Yes | Yes | Not reported | Yes | Not reported | No | N/A | N/A | Yes | Yes | N/A | No | N/A | N/A | No |
| **37.Orton E et al (2013) (UK) (Audit)** | Yes | Yes | N/A | Yes | Yes | N/A | Yes | N/A | Yes | Yes | N/A | Yes | N/A | N/A | Yes |
| **38.Paksin Hall A et al (2013) (USA)** | Yes | Yes | Not reported | Yes | Yes | N/A | N/A | N/A | Yes | Yes | N/A | Yes | N/A | N/A | Yes |
| **39.Pasagian MA et al (1997)** | Yes | Yes | Yes | Yes | Yes | No | N/A | N/A | N/A | N/A | N/A | Yes | N/A | N/A | No |
| **40.Paz SH et al (2006) (USA)** | Yes | Yes | Yes | Yes | Yes | No | N/A | N/A | Yes | Yes | N/A | Yes | N/A | N/A | Yes |
| **41.Puent BD et al (2004) (USA)** | Yes | Yes | No | Yes | Yes | N/A | N/A | N/A | N/A | N/A | N/A | Yes | N/A | N/A | N/A |
| **42.Rim TH et al (2013) (Korea)** | Yes | Yes | Yes | Yes | Yes | Not reported | N/A | N/A | Yes | Yes | N/A | Yes | N/A | N/A | Yes |
| **43.Saadine JB et al (2008) (USA)** | Yes | Yes | N/A | Yes | Yes | Not reported | N/A | N/A | Yes | Yeso | N/A | Yes | N/A | N/A | Yes |
| **44.Scanlon PH et al (2008) (UK)** | Yes | Yes | N/A | Yes | Yes | N/A | N/A | N/A | Yes | Yes | N/A | Yes | N/A | N/A | Yes |
| **45.Scanlon PH et al (2016) (UK)** | Yes | Yes | N/A | N/A | Yes | N/A | N/A | N/A | Yes | Yes | N/A | Yes | N/A | N/A | Yes |
| **46.Schmid KL et al (2003) (Australia)** | Yes | Yes | No | No | N/A | Not reported | N/A | N/A | Yes | Yes | N/A | Yes | Not reported | N/A | No |
| **47.Schoenfeld ER et al (2001) (USA)** | Yes | Yes | Not Reported | Yes | Yes | Not reported | N/A | N/A | Yes | Yes | N/A | Yes | N/A | N/A | Yes |
| **48.Sheppler CR et al (2014)** | Yes | Yes | Yes | Yes | Yes | No | N/A | N/A | N/A | N/A | N/A | Yes | N/A | Yes | Yes |
| **49.Shih HC et al (2007) (Taiwan)** | Yes | Yes | No | No | No | Not reported | N/A | N/A | Yes | Yes | N/A | No | N/A | N/A | Yes |
| **50.Srinivasan NK et al (2017) (India)** | Yes | Yes | Not reported | Yes | Yes | No | N/A | N/A | No | Yes | N/A | Yes | N/A | N/A | Yes |
| **51.Thapa R et al (2012) (Nepal)** | Yes | Yes | Not reported | Yes | Yes | Not reported | N/A | N/A | Yes | Yes | N/A | Yes | N/A | N/A | Yes |
| **52.Trento M et al (2002) (UK and Italy)** | Yes | Yes | Yes | No | Yes | Not reported | N/A | N/A | No | Yes | N/A | Yes | N/A | N/A | No |
| **53-a*. Van Ejik KN et al (2012) (Netherland) (Quantitative component)** | Yes | Yes | Yes | Yes | Yes | Not reported | N/A | N/A | Yes | Yes | N/A | Yes | N/A | N/A | No |
| **54.Walker EA et al (1997) (USA)** | Yes | Yes | Yes | Yes | Yes | Not reported | N/A | N/A | N/A | N/A | N/A | Yes | N/A | N/A | N/A |
| **55.Wang D et al (2010) (China)** | Yes | Yes | Yes | Yes | Yes | Not reported | N/A | N/A | Yes | Yes | N/A | Yes | N/A | N/A | Yes |
| **56.Xiong Y et al (2015) (China)** | Yes | Yes | Not reported | Yes | Yes | Not reported | N/A | N/A | Yes | Yes | N/A | Yes | N/A | N/A | Yes |
| **57.Yeo ST et al (2012) (UK) First** | Yes | Yes | Yes | Yes | Yes | Not reported | N/A | N/A | Yes | Yes | N/A | Yes | N/A | N/A | Yes |
| **58.Yeo ST et al (2012) (UK) Second** | Yes | Yes | No | Yes | Yes | Not reported | N/A | N/A | Yes | Yes | N/A | Yes | N/A | N/A | No |
| **59.Zhang X et al (2009) (USA)** | Yes | Yes | Yes | No | Yes | Not reported | N/A | N/A | Yes | Yes | N/A | Yes | N/A | N/A | Yes |

**Table 2.** Methodological Quality Assessment of Cohort Studies

|  | 1. *Validity of study results* | | | | | | | | 1. *Results* | | | 1. *General applicability of results* | | |
| --- | --- | --- | --- | --- | --- | --- | --- | --- | --- | --- | --- | --- | --- | --- |
|  | *1.Addressed a clearly focused issue (discontinue if ‘No’)* | *2.Acceptable method of cohort Recruitment (discontinue if ‘No’)* | *3.Exposure accurately measured to minimise bias* | *4.Outcome accurately measured to minimise bias* | *5.Identified all important confounding factors* | *6.Taken account of the confounding factors in the design and/or analysis* | *7.Complete enough follow up of subjects* | *8.Long enough follow up of subjects* | *9.Significance of the outcome difference between exposure groups given* | *10.Precision of the estimate mentioned* | *11.Results are believable* | *12.Results applicable to the general population* | *13.Study results fit with other available evidence* | *14.Implications of the study for practice noted* |
| **60.Bamashmus MA et al (2009) (Yemen)** | Yes | No | Yes | Yes | Yes | No | N/A (historical cohort) | N/A (historical cohort) | Yes | Yes | Yes | Not Reported | Not Reported | No |
| **61.Kreft D et al (2018) (Germany)** | Yes | No | Yes | No | No | Yes | Yes | Yes | Yes | Yes | Yes | No | Yes | Yes |
| **62.Maberley DA et al (2002) (Canada)** | Yes | No | No | Yes | Yes | Yes | Yes | Not Reported | Yes | No | Yes | Not Reported | Not Reported | Yes |
| **63.Storey PP et al (2016) (USA)** | Yes | No | Yes | Yes | Yes | Yes | Yes | Yes | Yes | No | Yes | Not Reported | Not Reported | Yes |

**Table 3.** Methodological Quality Assessment of Case Control Studies

|  | 1. *Validity of trial results* | | | | | | 1. *Results* | | | 1. *General applicability of results* | |
| --- | --- | --- | --- | --- | --- | --- | --- | --- | --- | --- | --- |
|  | *1.Addressed a clearly focused issue (discontinue if ‘No’)* | *2.Used an appropriate method to answer the question (discontinue if ‘No’)* | *3. Acceptable method of case Recruitment* | *4.Acceptable method of selecting controls* | *5. Exposure accurately measured minimising bias* | *6.Taken account of the confounding factors in the design and/or analysis* | *7.Estimate and/or significance of the difference in risk between groups given* | *8.Precision of the estimate mentioned* | *9.Results are believable* | *10.Results applicable to the general population* | *11.Study results fit with other available evidence* |
| **64.Lane M et al (2015) (UK)** | Yes | Yes | Yes | Yes | Yes | Yes | Yes | No | Yes | Not Reported | Not Reported |

**Table 4.** Methodological Quality Assessment of RCTs

|  | 1. *Validity of trial results* | | | | | | 1. *Results* | | 1. *General applicability of results* | | |
| --- | --- | --- | --- | --- | --- | --- | --- | --- | --- | --- | --- |
|  | *1.Addressed a clearly focused issue (discontinue if ‘No’)* | *2.Randomised assignment of patients to treatments (discontinue if ‘No’)* | *3. Patients, health workers and study personnel blinded* | *4.Groups similar at start of the trial* | *5. Groups treated equally other than for the intervention* | *6.All patients entered in the trial properly accounted for at conclusion* | *7.Treatment effect size mentioned* | *8.Precision of the treatment effect size mentioned* | *9. Results applicable to the general population* | *10.All clinically important outcomes considered* | *11.Benefits worth the harms and costs* |
| **65.Basch CE et al (1999) (USA)** | Yes | Yes | Yes (not patients) | Yes | Yes | Yes | Yes | Yes | No | No | No |
| **66.Lian JX et al (2013) (Hong Kong)** | Yes | Yes | Yes (not patients) | Yes | Yes | Yes | Yes | Yes | No | No | Yes |
| **67.Hazavehei SMM et al (2010) (Iran)** | Yes | No | Not Reported | No | Not Reported | Not Reported | Yes | No | Not Reported | No | Not Reported |

**Table 5.** Methodological Quality Assessment of Qualitative Research

|  | *1.Clear statement of the aims of the research (discontinue if ‘No’)* | *2.Appropriate to use a qualitative methodology (discontinue if ‘No’)* | *3.Research design appropriate for addressing aims of the research* | *4.Recruitent strategy appropriate for aims of the research* | *5.Data collected in a way that addressed the research issue* | *6.Relationship between researcher and participants adequately considered* | *7.Ethical issues have been taken into consideration* | *8.Data analysis sufficiently rigorous* | *9.Clear statement of findings* | *10.The research has practical value* |
| --- | --- | --- | --- | --- | --- | --- | --- | --- | --- | --- |
| **68.Glasson NM et al (2017)** | Yes | Yes | Yes | No | Yes | Not Reported | Yes | Yes | Yes | Yes |
| **69.Hartnett ME et al (2005) (USA)** | Yes | Yes | Yes | Yes | Yes | Yes | Not Reported | Yes | Yes | Yes |
| **70.Hipwell AE et al (2014) (UK)** | Yes | Yes | Yes | Yes | Yes | No | Yes | Yes | Yes | Yes |
| **71.Katibeh M et al (2017) (Iran) First** | Yes | Yes | Yes | Yes | Yes | No | Yes | Yes | Yes | Yes |
| **72.Lake AJ et al (2017) (Australia)** | Yes | Yes | Yes | Yes | Yes | Not Reported | Yes | Yes | Yes | Yes |
| **73.Lewis K et al (2007) (UK)** | Yes | Yes | Yes | Yes | Yes | Yes | Yes | Yes | Yes | Yes |
| **74.Lindenmeyer A et al (2014) (UK)** | Yes | Yes | Yes | Yes | Yes | No | Yes | Yes | Yes | Yes |
| **75.Liu Y et al (2018) (USA)** | Yes | Yes | Yes | Yes | Yes | Not Reported | Yes | Yes | Yes | Yes |
| **53-b*. VanEijk KN et al (2012)** | Yes | Yes | No | No | No | Not Reported | Yes | No | Yes | Yes |

**VanEijk KN et al conducted using mixed methods. Two reviews articles were not included in quality analysis table as those were not complied with assessment criteria. (1-Burgess PI el al, 2013. 2-Khandekar R et al 2012).*
