# Supplementary material for: Systematic review on barriers and enablers for access to diabetic retinopathy screening services in different income settings
Source: PLoS One. 2019 Apr 23;14(4):e0198979. doi: 10.1371/journal.pone.0198979 (PMC6478270; doi:10.1371/journal.pone.0198979)
Supplement: S4 Table — (DOCX) [file pone.0198979.s004.docx]

**S4 Table. Themes tables by country income setting**

**S4 Table 1 – LIC**

| **Study 1^st^ Author Name, Year and Setting** | **Modality of screening** | **Consumer Barriers** | **Consumer Enablers** | **Provider Barriers** | **Provider Enablers** | **System Barriers** |
| --- | --- | --- | --- | --- | --- | --- |
| **1.Burgess, P.I. et al (2013) (Africa)**  **[LIC]** | Not mentioned | Poor patient attendance | Not mentioned | Lack of skilled human resources and training,  Lack of access to imaging and DR treatment infrastructure,  Lack of a systematic monitoring system for complications of diabetes,  Non-existence of a referral system,  Non-existence of diabetes multidisciplinary healthcare teams | Increase human resources (ophthalmologists) and sub-specialisation,  Provision of imaging and treatment infrastructure,  Provision of tertiary retinal care and training,  Development of retinal research networks,  Prioritization of sub-specialty development in post-graduate training programs and facilitate knowledge and skills sharing | Competing disease priorities,  Lack of a national policy,  Non-existence of a systematic screening program,  Poor record keeping and lack of infrastructure to support services,  Provision of retinal fellowships tailored to developing world trainees in retinal centres in developed countries |
| **2.Mumba, M. et al (2007) (Tanzania)**  **[LIC]** | Dilated fundus examination by ophthalmologist | Lack of knowledge on eye complications (OR),  Lack of awareness about important of eye exam,  Lack of knowledge on availability of eye clinics | Knowledge on eye complications (OR) | Not mentioned | Counselling | Distance to the institution |
| **3.Thapa, R. (2012) (Nepal)**  **[LIC]** | Detailed fundus evaluation after dilation in a vitreo retinal clinic | Lack of awareness of diabetes ocular complications | Awareness and literacy (OR),  Having a family member with diabetes (OR),  Prior fundus evaluation elsewhere (OR) | Not mentioned | Not mentioned | Place of living (in a geographically diverse setting) (OR),  Short time duration with patients due to lower doctor-patient ratio |

**S4 Table 2 – LMIC**

| **Study 1st Author Name, Year and Setting** | **Modality of screening** | **Consumer Barriers** | **Consumer Enablers** | **Provider Barriers** | **Provider Enablers** | **System Barriers** |
| --- | --- | --- | --- | --- | --- | --- |
| **4.Abdulsalam S. et al, 2018, (North-Western Nigeria)**  **[LMIC]** | Fundoscopy | Not mentioned | Not mentioned | Lack of knowledge on DR among the physicians  Lack of functional ophthalmoscopes  Lack of dilating eye drops  Lack of skills in identifying DR signs  Poor attitude on dilating pupils, and taking responsibility of DR screening | Training physicians on DR screening  Conduct the eye examination by the physicians rather than referring  Variable duration of refresher training, essential to improve DR screening skills. | Lack of human resources for DR screening |
| **5.Adriono, G. et al (2011) (Indonesia)**  **[LMIC]** | Dilated eye examination by an eye care professional | Lack of knowledge, attitude, awareness on DR (OR),  Financial barriers,  Feeling of no need if vision is good,  Lack of understanding of the role of eye examination | Knowledge, attitude, awareness on DR (OR),  More severe diabetes and comorbidities (OR) | Financial barriers (cost of services),  Lack of diabetic health education | Information given by the service provider (OR),  Having told of the need of regular eye examination,  Educational strategies aimed both patients and physicians | Financial implications in having an insurance coverage |
| **6.Agarwal, S. et al (2005) (India)**  **[LMIC]** | Dilated fundus examination by binocular indirect ophthalmoscopy (screening camp setting) | Unawareness of diabetes eye complications,  Poor motivation,  Other priorities, Fear, Spirituality (faith and hope) | Not mentioned | Not mentioned | Awareness campaign with integrated team,  Educational programs by meetings, distribution of information leaflets and through media | Economic and logistic reasons |
| **7.Bamashmus, M.A. et al (2009) (Yemen)**  **[LMIC]** | Dilated bio microscopic examination by ophthalmologist | Lack of knowledge,  Disadvantaged health habits,  Disability, being handicapped | Regular clinic visits (OR) | Challenges faced by ophthalmologists in dealing with blindness,  Lack of resources/facilities,  Need of frequent and costly investigations | Not mentioned | Cost of investigations in private labs,  Lack of health programs |
| **8.Islam FMA et al, 2018, (Bangladesh) [LMIC]** | Digital imaging in a rural community clinic | Poor health literacy on DR and poor general education  No felt need of DR screening, when asymptomatic  Lack of time  Fear of complications.  Longer time to recover after pupil dilation | Increase health literacy of the users | Lack of skill of confidence upon skills of ophthalmic assistants  Lack of availability of DR treatment facilities | Make people aware of the detrimental effects of DM. | Shortage of number of ophthalmologists overall and maldistribution in rural areas |
| **9.Kurji, K. et al (2013) (Kenya)**  **[LMIC]** | Tele-ophthalmology by dilated digital fundus imaging | Low literacy | Patient satisfaction over the modality  Time saving and convenient method,  Ability to see the fundus images by patients | Low number and inadequate distribution of medical specialists,  Low availability of diagnostic and treatment equipments and medications | Convenience, Reduced examination time, Ability to visualize own retina, and less cost (tele-ophthalmology)  Utilization of more convenient avenues for communication (in sending reports through registered mail / secure electronic mail) | Low medical insurance coverage,  Challenges in transport and communication |
| **10.Muecke, J.S. et al (2008) (Myanmar)**  **[LMIC]** | Not mentioned | Lack of understanding of the nature and treatment of DR,  Lack of schooling (OR),  Inconvenience or a fear of visiting a specialist,  Thought of examination needed only if there are problems,  Inability to pay for private health care | Development of visual symptoms related to DR | Lack of resources (ophthalmoscopy equipment),  Time constraints (for fundus examination in busy urban practice),  Lack of awareness among GPs, (technique and signs of DR)  Poor opinion of the quality of service available in the public eye centres,  Lack of action by GP in examining the fundus | A reminder of the serious consequences  of the failure to examine fundi of diabetic patients,  Repeated reminder from GP,  Information pamphlets, posters and medical education seminars for GPs,  Public health education by media | Lack of training for GPs and increased work load,  Lack of epidemiological studies  Lack of optometrists for primary screening,  Accessibility to eye centres. |
| **11. Mwangi N. et al 2017 (Kenya) [LMIC]** | Eye examination at an eye clinic after referral or opportunistic | Lack of knowledge about DM and DR.  Misconceptions about diabetes related eye disease.  Not following up on referrals. | Written communication from the patient’s ophthalmologist to the primary care provider | Not mentioned | Having a referral letter | Not mentioned |
| **12.Namperumalsamy, P. et al (2004) (India)**  **[LMIC]** | Not mentioned | Lack of knowledge and awareness on DR,  Gaps between attitude knowledge and actual practice | Better understanding of risk factors | Lack of knowledge and awareness on DR | Health education | Not mentioned |
| **13.Onakpoya, O.H. et al (2010) (Nigeria)**  **[LMIC]** | Mydriatic direct ophthalmoscopy | Lack of knowledge on DR,  Not having eye problems or symptoms | Presence of visual impairment or blindness and non-diabetic vision threatening eye diseases | Low referral rates,  Lack of adequate knowledge on DR among PCPs | Trained non-ophthalmologist physicians screen diabetics for  DR regularly at endocrinology clinic,  Availability of fundus camera,  Periodic review with ophthalmologists,  Health education on DR and DR screening | Not mentioned |
| **14. Sirinivasan NK et al 2017 (India)**  **[LMIC]** | Dilated fundus examination using slit lamp binocular indirect ophthalmoscopy. | Lack of knowledge on DM,  Lack of awareness of DR, Lack of awareness of importance  Affordability,  Poor family support, did not find time, physically unwell. | Good Knowledge on DM | Not mentioned | Health education by the doctor | Long distance to the hospitals |

**S4 Table 3 – UMIC**

| **Study Author Name and Year** | **Modality of screening** | **Consumer Barriers** | **Consumer Enablers** | **Provider Barriers** | **Provider Enablers** | **System Barriers** |
| --- | --- | --- | --- | --- | --- | --- |
| **15.Cetin, E.N. et al (2013) Turkey [UMIC]** | Not mentioned | Lack of knowledge, attitude and awareness on DR | Knowledge, attitude, awareness on DR,  Higher education level attainment  Attending endocrinology department for medical care (tertiary level),  DM education (OR),  Knowing need of annual examination | Changing awareness among physicians  Less referrals from state hospital | Physician’s recommendation,  Awareness among physicians regarding eye complications of diabetes,  Establishment of referral guidelines,  Targeted education programs | Not mentioned |
| **16.Hazavehei, S.M.M. et al (2010) (Iran)**  **[UMIC]** | Examination by ophthalmologist | Lack of knowledge, awareness and attitude (Mean scores) | Not mentioned | Not mentioned | Educational intervention | Availability of health educational interventions |
| **17.Katibeh et al 2017 (2^nd^ Article) (Iran) [UMIC]** | Masked grading of stereoscopic fundus photographs | Low literacy level associated with poor awareness on DR | Higher secondary level education, properly controlled HbA1c | Lack of source of awareness | Having educational strategies  Physicians as a source of information | Disparity in services between rural and urban settings. |
| **18.Katibeh M. et al 2017. (Iran) [UMIC]** | Not specified | Not mentioned | Not mentioned | Provider unawareness on guidelines.  Poor usage of available DM and DR prevalence data.  Poor referral systems  Lack of retinal imaging technology at lower levels of service delivery  Lack of systematic recall system | Availability of continuous medical education | Strengthened clinical guidelines on DR.  Coverage of whole country in a national plan.  Poor transport systems.    Cost of services  Lack of health information systems  Poor auditing systems |
| **19.Khandekar, R. et al (2012) (Meditarranean)**  **[UMIC]** | Not mentioned | Inertia to change life style | Not mentioned | Scarce human resources and material,  Weak health systems,  Cost of technology and resources,  Palliative nature of DR management | Training human resources,  Health education on DR,  Involvement of community and patient groups | Limitations in public health approach,  Civil unrest and poverty related health issues |
| **20.Wang, D. et al (2010) (China)**  **[UMIC]** | Not mentioned | Lack of awareness and knowledge of DR,  Poor language fluency,  Less well educated,  Lower monthly income,  Misconceptions- as DR having early symptoms and screening unnecessary without symptoms | More concern about vision loss (OR),  Knowledge on DR (OR) | Lack of training,  Lack of programs to raise awareness,  Poor physician–patient communication (Not telling the importance of regular eye examinations),  Inability to cope with the large number of diabetes patients, | Recommendation and education of importance of eye examination (OR),  Attendance to the urban tertiary and community hospitals | Disparity in services between rural and urban settings (OR) |
| **21.Xiong, Y. et al (2015) (China)**  **[UMIC]** | Non-mydriatic digital retinal imaging | Poor health education  Lack of awareness and income,  Lack of understanding of early treatment as the key to prevent blindness | More educational level (OR),  Severe DR stage (OR),  Vision loss | Lack of communication between the patients and doctors,  Unclear explanations of disease (DR) and treatment,  Medical costs | Community health management network coverage,  Community based health education,  Effectively locate DR patients who unaware of the disease | Medical insurance (OR),  Transportation |

**S4 Table 4 – HIC**

| **Study Author Name and Year** | **Modality of screening** | **Consumer Barriers** | **Consumer Enablers** | **Provider Barriers** | **Provider Enablers** | **System Barriers** |
| --- | --- | --- | --- | --- | --- | --- |
| **22.Anderson, S. et al (2003) (UK)**  **[HIC]** | Slit lamp examination following dilatation and 6feild fundus photography (hand held) | Poor cooperation and problems in mobility,  Refusal of screening,  Medically unfit to attend screening | Not mentioned | Level of experience of the screener,  Lack of effective way for screening of confused or immobile patients | Not mentioned | Home bound diabetics |
| **23.Basch, C.E. et al (1999) (USA)**  **HIC** | Dilated retinal examination | Lack of knowledge,  Low Literacy and awareness | Not mentioned | Lack of Health education | Educational intervention (OR),  Focusing on high risk group,  Intervention on broad scale | Not mentioned |
| **24.Baumeister, S.E. et al (2015) (Germany)**  **HIC** | Not mentioned | Lower socio-economic status (OR),  Living alone (OR),  Lower educational attainment (OR),  Lower income (OR),  Poorly controlled diabetes (OR),  Diabetes related co-mobidities (OR),  Employed and unemployed individuals (OR),  Patients visits GPs or Internists for care (OR) | Poor self-reported health (OR),  Lower physical and mental health-related quality of life (OR),  Adherence to best practice guidelines, Having DR (OR) | Lack of attention by GPs,  Non-adherence to guidelines | Better screening, detection and management,  Adherence to best practice guidelines,  Screening services, preventive  services, self-management education and counselling integrated within primary care,  Focused screening (Patients with poor DM control, complications and co-morbidities) | Lack of proper referral and reminding system,  Lack of insurance coverage (OR),  Lower socio-economic position |
| **25.Bennet GH et al 2018 (Ireland) [HIC]** | Not mentioned | Difficulties in getting appointments | Having non-ocular complications | Inconveniences in referral mechanisms | Online registration and appointment systems | Not mentioned |
| **26.Brechner, R.J. et al (1993) (USA)**  **HIC** | A dilated eye examination | Lower income (OR),  Level of education (OR),  Less frequency of physician visits for DM | Higher socioeconomic status,  Having diabetes education class (OR) | Not inform the patients about having DR (OR),  Not having education class | Having told to have DR | Health insurance |
| **27.Creuzot, G.C. et al (2014) (France)**  **HIC** | 3F digital photography with or without dilatation | Lack of awareness of eye care,  Problems in access to GP,  Cost effectiveness of the treatments,  Not feeling the symptoms,  Not feeling concerned about it (asymptomatic nature) | Declared frequency of ophthalmic visit every 2 years  Awareness of eye care (OR) | No adequate patient education system,  Lack of information provided to patients  Problems in access to GP or ophthalmologist | Mobile DR screening,  Patient education and information,  Recommendation | Low GP density, |
| **28.Dervan, E. et al (2008) (Ireland)**  **HIC** | A dilated eye examination | Lack of knowledge regarding eye examination (OR),  Lack of awareness,  Effect of mydriasis prohibiting driving,  Lack of concern about the vision,  Wrong expectation of screening as a routine | Think eye examination is needed every 6 months (OR),  Expect eye examination as part of routine,  Worrying about vision,  Aware of developing an eye disease and having symptoms,  History of DR or another eye disease (OR) | Lack of physician recommendation,  Lack of appointments,  Requirement of mydriasis,  Unaware of importance of mydriatic funduscopy by examining physician | Having told by physician to have  regular eye examinations (OR),  Reinforcing the importance of eye examination by health care providers | Not mentioned |
| **29.Eiser, J.R. et al (2001) (UK)**  **HIC** | Dilated polaroid photography followed by direct ophthalmoscopy | Lack of knowledge,  Lack of flexibility in adjusting attitude and behaviour,  Reluctance to change self-management,  Reluctant to make behavioural changes | Having diabetes related eye problems | Not mentioned | Retinal screening services organized in primary care settings,  Showing image of retina to patients (irrespective of their understanding),  Information to patients which easy to understand | Not mentioned |
| **30.Foreman, J. et al. (2017) (Australia) [HIC]** | National level DR screening | Lack of awareness of the NHMRC eye examination guidelines.  Unaware of the need for regular eye examinations.  Being indigenous status.  Lack of time. | Longer duration of diabetes.  Living in an inner regional locality | Missed appointments. | Integrated DR screening services especially in remote areas.  Improved referral pathways.  Health education | Unavailability of services in remote areas. |
| **31.Gillibrand, W.P. et al (2000) (UK)**  **[HIC]** | Dilated 3 field fundus photography | Lack of knowledge of DM (can cause eye complications) | Knowledge on diabetes control,  Self-perceived importance of good control of DM | Limited knowledge in health care professional | Health education strategies (as required by individual) | Local health promotion services for all groups |
| **32.Glasson, NM. et al. (2017) (Australia) [HIC]** | Remote outreach DR Screening modality | Difficulty in getting leave from the employment to attend screening.  Financial constrains in travelling long distances  Dislike pupil dilation and camera flash light | Not mentioned | Lack of infrastructure (clinic space).  Lack of training and education (at nurse screener and GP grader level)  Lack of communication, coordination and information sharing. | Improved, efficient access through outreach screening  Acceptability of remote screening | Long travel distances and lack of transportation  Governance and operational elements (identification of people with DM at community level) in service delivery  Financial implications  Sustainability  Quality and safety  Coordination and integration of services |
| **33.Gulliford, M.C. et al (2010) (UK)**  **HIC** | 2 field digital photography | Socio economic inequalities with regard to ethnicity (OR),  Socio economic deprivation (OR) | Not mentioned | Not mentioned | Not mentioned | Socio economic inequalities |
| **34.Hartnett, M.E. et al (2005) (USA)**  **[HIC]** | Dilated fundus imaging | Lack of insight, education, knowledge on diabetes and blindness,  Multiple appointments at one time,  Lack of understanding the rationale of annual exams and about DR,  Burden of diabetes and treatment overshadowing eye disease,  Personal issues (child care concerns, transportation difficulty, work, and forgetting appointments),  Not remembering physician’s names,  Gap between patient education and their understanding,  Financial burden | Fear of blindness,  Attending DM education classes | Cost of services,  Poor physician-patient communication,  Poor access to care-long wait for appointment, long clinic waiting time, large number of patients per doctor,  Frequent change of staff,  Lack of understanding needs in-between specialities,  Medical records not always available | Electronic medical records or email | Transportation,  Unavailability of medical records on time |
| **35.Harvey, J.N. et al (2006) (UK)**  **[HIC]** | Mydriatic imaging by optometrists and direct ophthalmoscopy by ophthalmologists | Failure to attend (Move away or died),  Failure to keep appointments,  Not appreciate the importance of eye screening,  Cost incurred by screening | Not mentioned | Unavailability of (digital retinal) screening,  Failure to refer by GP | Identifying and targeting non-attendees | Diabetes register, complete records and communication system |
| **36.Hipwell, A.E. et al (2014) (UK) [HIC]** | Mydriatic fundus photography | Denial of having diabetes,  Understanding of the importance of screening,  Dislike the method of screening - proximity, Side effects and adverse effects of mydriatics (unable to drive),  Confusions about DR screening vs routine eye check,  Work commitments,  Postoperative recuperation,  Residential changes,  Problems of making appointments,  Forgetfulness especially working people, Psychological, pragmatic and social factors, | Protecting the eye as priority,  Proximity of screening clinic to patient’s homes,  Knowledge about DR and screening | Perception of making appointments,  Length of appointment and duration of food abstinence,  Absence of appointments,  Failure to deliver the right message,  Side effects and adverse effects of mydriatics (significant pain and visual disturbances),  Unable to drive after mydriasis | Integrating DR screening with diabetic care,  Efficient GP practice appointments,  Short and efficient appointments,  Convenience and transport safety | Transport,  Temporary accommodation,  Lack of media attention,  Appointment booking system issues |
| **37.Huang, O.S. et al (2009) (Singapore)**  **[HIC]** | Dilated retinal photography by digital retinal camera | Awareness of DR/DM status,  Less educational level (OR),  Apathy, Patient denial | Severity of DR | Poor patient-doctor communication | Emphasise the need for regular examination,  Patient education and their initiatives | Lack of health education |
| **38.Hwang, J. et al (2015) (Canada)**  **[HIC]** | A dilated eye examination | Low income (OR),  Lack of private health insurance (OR),  Poor self-rated health status (visual impairment) (OR) | Having visual impairment (symptomatic) (OR),  Discussion of DM complication with a health care professional (OR),  Having private health insurance,  Highest income | Not discuss about diabetes complications | Educating the importance of DR screening by PCPs | Private health insurance (OR) |
| **39.Khandekar, R. et al (2008) (Oman)**  **[HIC]** | Not mentioned | Not mentioned | Not mentioned | Limited knowledge, attitude and practice of physicians,  Limited experience of using the ophthalmoscope  during undergraduate training and  professional practice,  Lack of attention to diabetic eye care (in eye care workshops). | Training of primary staff by regional ophthalmologists | Availability of training programs |
| **40.Kreft D. et al 2018. (Germany) [HIC]** | DR screening by an ophthalmologist - National screening | Multiple comorbidities and high level of disability. | Low severity of diabetes  Participation in a educational program | Not mentioned | Educational programs | Not mentioned |
| **41.Lane, M. et al (2015) (UK)**  **[HIC]** | Annual digital mydriatic fundus photography | Poor language skills,  Knowledge gap,  Socioeconomic status | Not mentioned | Long waiting time for treatment | Not mentioned | Social deprivation and ethnicity |
| **42.Lake A.J. et al 2017 (Australia) [HIC]** | Australian national DR screening programme  (dilated bio-microscopy - community setting) | Social influences.  Anticipated regret and perceived vulnerability  Concern on impact of the family unit  Lack of financial resources  Misconceptions on DR knowledge  Absence of planning | Beliefs about the consequences of missed screening  Positive reinforcement through negative screening results  Positive intentions and emotions to check eyes  Intention to commence or maintain screening | Lack of clinician’s recommendations  Clinical inertia  Reluctance to acknowledge about DR by providers (on young patients) | Influences from professionals (GP). | Cost of the procedures |
| **43.Lee, P.P. et al (1998) (USA)**  **[HIC]** | Dilated fundus examination | Lack of knowledge on eye care,  Cost,  Healthcare related behaviour,  Age | Not mentioned | Lower use of specialists’ care,  Low rate of diabetic eye screening | Not mentioned | Better managed care plans,  Geographic and demographic factors |
| **44.Leese, G.P. et al (2008) (UK)**  **[HIC]** | Digital retinal photography by mobile retinal cameras | Social deprivation (OR) | Having visual symptoms | Invited to eye vans rather than static unit (OR) | Mobile retinal cameras,  Structural and patient-system evaluation,  Constant screening location (OR) | Social deprivation (OR),  Travel time |
| **45.Lewis, K. et al (2007) (UK)**  **[HIC]** | Regular eye examination by digital fundus camera | Lack of awareness (DR could lead to blindness and could be asymptomatic),  Fear, guilt and family attitude,  Problems in career,  Limited knowledge of DR and misunderstanding of risk factors,  Not appreciate the long-term follow-up, | Fear of losing vision | Waiting time at eye clinic,  Reluctant to ask questions and difficulties in accompanying patients,  Awareness on DR among the providers,  Reluctant to discuss the possibility of blindness,  Little education provided by eye clinic,  Underestimate the difficulties faced by patients in obtaining time off work to attend,  Reluctance of diabetic educators to dwell on negative consequence of DM,  Reluctant to mention blindness,  Fear of laser,  Eye clinics tend to run late, and patients compete for early appointments | Using screening images as educational material,  Education on asymptomatic disease and risk of blindness,  Reinforce the importance of eye care,  Avoid giving impression that DR is due to carelessness and poor control,  Evening clinics,  Early involvement of social workers,  Reorganization of clinic bookings | Transport |
| **46.Lian, J.X. et al (2013) (Hong Kong)**  **[HIC]** | Non-mydriatic fundus camera | Cost of the services (Affordability),  Socio economic factors,  Being in a pay group (OR) | Higher family income (OR),  Currently not working (OR)  Welfare of recipients (OR), | Fee for services (Cost of services),  Co-payment for screening | Not mentioned | Not mentioned |
| **47.Lian J et al, 2018, (Hong Kong)**  **[HIC]** | Not mentioned | Specific deficits in knowledge that early DR can be asymptomatic and availability of treatment  Having specific knowledge (as assumed by the patient) on how often screening should be performed | Lack of knowledge on frequency of screening  Worry about vision loss  Awareness on importance of eye examination | Not mentioned | Increasing patient awareness of DR screening.  Recommendation of screening by the health care provider | Not mentioned |
| **48.Lindenmeyer, A. et al (2014) (UK)**  **[HIC]** | Mydriatic digital retinal photographs | Poor English fluency,  Perception of non-attenders | Experience symptoms | Less space for screening,  Lack of communication between screening services and practices | Contacting and motivating patients,  Integrating screening with routine care,  Integrating/Focusing the newly  Diagnosed,  Communication between screening services and practices | Transport and access,  Social deprivation,  Diversity of ethnicities and languages |
| **49.Liu Y, et al, 2018, (United States of America)**  **[HIC]** | Not specified | Multiple health conditions  Poverty and financial trade-offs  Limited health literacy  Infrequent use of health care  Burden of DM management  Negative self-perception  Anxiety related to DM complications  Experiences with family members struggles with DM complications - led to fear of receiving bad news. | Surveillance and judgment from family, friends and providers  Trust in health care provider  Motivation due to anxiety related to diabetes complications | Not specified | Recommendations of health care provider  Teleophthalmology may complement patient education by addressing the environment barriers    Teleophthalmology present in primary care clinics in rural areas  Trust on the providers | Long travel distances to obtain health services (lengthy travel time and transportation barriers)  Limited access to health care  Policies to improve reimbursements for teleophthalmology |
| **50.Maberley, D.A. et al (2002) (Canada)**  **[HIC]** | Not mentioned | Lack of awareness and knowledge,  Reluctant to seek medical care | More advanced DM,  Likely to have DR | Lack of human resources - Physicians,  Lack of availability of educational programs,  Less advice given to younger patients,  Less opportunity and less education for younger patients,  Less aggressive in encouraging younger patients | Reserve-based diabetes education programs | Inadequacy of clinics and hospitals in the region,  Distance to the service provider (Residence) (OR),  Inadequate physician resources |
| **51.Moss, S.E. et al (1995) (USA)**  **[HIC]** | Dilated ophthalmoscopy followed by 7F stereoscopic colour fundus photos | Perception of no problem with eyes when asymptomatic,  Not liking the pupil dilatation,  Behaviours (smoking and drinking),  Years of education,  Family income,  Thought of no problems in eyes means no need to examine,  Could not afford an examination,  Being working people (busy),  Fear of prognosis or needing treatment | Higher education,  Currently not working,  Thought of at least every12 month exam is needed (OR),  More severe retinopathy (OR),  History of cataract or glaucoma (OR),  Higher income, Health insurance that covered eye examination (OR),  Visually impaired,  Having eyes examined by PCPs | Lack of provision of information,  Difficulties in getting an appointment,  Not having been told eye examination is needed | Having told annual examination in needed (OR),  Recommendation to continue seeing the eye physician,  Impress on importance of annual examination,  Flexible office hours on the part of ophthalmologists and optometrists including evenings and weekends,  Education on need for eye exam and treatable nature of DR,  PCPs familiarize with guidelines of eye exam,  Attending to internists | Geographical accessibility,  Financial barriers,  Far to the optometrists or ophthalmologists or difficult to get a ride |
| **52.Moreton R.B.R. et al 2017.**  **(United Kingdom)**  **[HIC]** | Oxfordshire DR screening program | Socio-economic deprivation  Younger age | Older age | Screening by high street optometrists | Higher uptakes for those invited for screening by mobile units | Not mentioned |
| **53.Mukamel, D.B. et al (1999) (USA)**  **[HIC]** | Dilated fundus examinations by an ophthalmologist or an optometrist | Socio demographic and economic characteristics | Contact with the PCPs | Less visiting frequency (OR),  Lack of correlation between PCPs and screening | Higher referrals by GP,  Under care of PCPs,  High patient expenditure (OR),  Increased interaction and increased chance to educate patients,  Interventions addressing both patients and physicians (All PCPs and targeted patents with certain ethnicity and limited education),  Higher visiting frequency to PCP (OR) | Lack of correlation between PCP specialty and screening services,  Living in lower educational and income areas (OR) |
| **54.Munoz, B. et al (2008) (USA)**  **[HIC]** | Dilated eye examination | The lack of correct information and awareness,  Poor educational level (OR),  Poor language fluency,  Financial constraints,  Poor level of knowledge on diabetic eye complications | Long-time residing in place (OR),  Having insurance (OR),  Higher education (OR) | Less options and access for eye care professionals,  Incomplete explanation of the diseases to patients,  Providers’ poor language fluency | Increase awareness using health educational materials (in local language and according to educational level of target population),  Personalised strategies such as phone calls and door to door visits,  Flexible schedules on screening | Having insurance (OR) |
| **55.Murgatroyd, H. et al (2006) (UK)**  **[HIC]** | Mydriatic and non-mydriatic photography | Discomfort and effects following mydriasis (driving, working outside home),  Unacceptability of mydriasis specially by patients who had previous non-mydriatic examination technique | Non-mydriasis | Not mentioned | Education on mydriatic drops,  Targeted use of mydriasis and age relate strategies | Not mentioned |
| **56.Newcomb, P.A. et al (1990) (USA)**  **[HIC]** | 7F Stereoscopic fundus photographs | Lack of awareness,  Financial constrains | Experiencing vision loss,  More educational levels,  Had been seen previously by ophthalmologist,  More frequent insulin reaction,  Previous diagnosis of eye disease,  Better DM control,  More severe DR,  Knowledge of pre-existing diabetic eye disease,  Perceptions of personal susceptibility,  Severity of  the consequences of retinopathy, | Poor physician availability | Telling patients that they have affected eyes,  Screening recommendations | Being in a rural area,  Metropolitan residence |
| **57.Newcomb, P.A. et al (1992) (USA)**  **[HIC]** | 7F Stereoscopic fundus photographs | Attitudinal issues (possess awareness but lacks compliance),  Less degree of knowledge | Not mentioned | Not mentioned | Not mentioned | Not mentioned |
| **58.Orton, E. et al (2013) (UK)**  **[HIC]** | An eye screening | Patients’ understanding about screening,  Other health issues as higher priority,  Forget to make appointments,  Lack of awareness and knowledge,  Preference of booking appointments,  Perceiving no eye problems as not necessary to screen again | Not mentioned | Comprehensiveness of the Information given by the service provider | Talking about screening by GP / Nurse,  Written information leaflets,  Primary care level changes - Simplify the screening invitation letters, Use the term diabetic eye disease rather than retinopathy, contact by post rather than waiting for the next visit,  Establishing a direct line between practices to the screening booking team,  Online patient access booking and text reminder service,  Maintaining the availability of out of hours screening provision and agreement,  Working with the  practices to minimise the exclusion of patients from screening,  Reconciling practice and screening patient listings | Increasing deprivation (city vs county) (OR),  No nationally specified screening programmes |
| **59.Paksin Hall, A. et al (2013) (USA)**  **[HIC]** | Dilated eye examination | Lack of awareness and knowledge | Being married (OR),  DM education class (OR),  Had feet checked within the last year by a health professional (OR),  Higher income (OR),  More education (OR),  Fewer days of unhealthy mental status (OR) | Not mentioned | Diabetes education | Availability of health insurance (OR) |
| **60.Pasagian, M.A. et al (1997) (USA)**  **[HIC]** | Dilated annual eye examination | Lack of knowledge and awareness,  Financial constraints,  Transportation, traveling alone after having their eyes dilated,  Difficulty getting someone to accompany,  Cost of the examination,  Difficulty getting to clinic | Being concerned about eye complication,  Higher level of education | Lack of Human resources,  Long waiting time in the clinic or doctor’s office,  Lack of individually scheduled appointments,  Length of getting an appointment | Educate patients and counselling | Lack of SPs,  Transportation problems |
| **61.Paz, S.H. et al (2006) (USA)**  **[HIC]** | Dilated 7F stereoscopic fundus photography | Less education level (OR),  Knowledge on DR,  Less annual income (OR),  Lack of routine medical care,  Lack of annual physical exam (OR) | Having eye disease (glaucoma or DR) | Lack of provision of health education | Educational programs | Lack of health insurance schemes |
| **62.Puent, B.D. et al (2004) (USA)**  **[HIC]** | Annual dilated eye examination | Limited personal mobility due to poor overall health,  Low socio-economic status,  Misunderstanding of benefits of health insurance,  Lack of understanding in periodic eye examination,  Not accept diagnosis of DM and periodic care,  Forgetfulness,  Extended vacation,  Other illness (reason cancer treatment),  Self-reported apathy | Not mentioned | Transferring of eye care to another doctor,  Pupil dilatation,  Last examination at a homeless clinic,  Discontinuity of eye care | Chief symptom of need for diabetic examination,  Maintain recall system,  Educating patient on how to use insurance for eye care | Lack of health insurance |
| **63.Rim, T.H. et al (2013) (Korea)**  **[HIC]** | Fundus photography | Financial constraints,  Lack of education on diabetes and reluctant to undergo re-examination,  Lack of time | Higher level of education (OR),  Self-reported unhealthy health status (OR),  Having other comorbidities (OR) | Lack of ophthalmologists and primary physicians in rural areas | Not mentioned | Socio-economic discrepancies (urban vs rural) (OR),  Difference in health systems |
| **64.Saadine, J.B. et al (2008) (USA)**  **[HIC]** | A dilated eye examination | Chronic diseases | Worse acuity and DR level (OR) | Not mentioned | Not mentioned | Not mentioned |
| **65.Scanlon, P.H. et al (2008) (UK)**  **[HIC]** | Mobile camera - digital photography | Lack of financial support | Not mentioned | Not mentioned | Not mentioned | Socio-economic deprivation (OR) |
| **66.Scanlon, P.H. et al (2016) (UK)**  **[HIC]** | Eye screening programme-Mydriatic digital imaging | Socio-economic deprivation | Not mentioned | Factors related to primary care practices and screening team | Not mentioned | Socio-economic deprivation |
| **67.Schmid, K.L. et al (2003) (Australia)**  **[HIC]** | Not mentioned | Lack of awareness and knowledge among the less educated,  Financial issues,  Poor English fluency,  Thought of already under care of a specialist | Awareness and Knowledge,  Being a member of Diabetes organisation (Diabetes Australia) | Optometrists - lack of adequately trained and not having correct equipment | Educating patients | Being in a rural area |
| **68.Schoenfeld, E.R. et al (2001) (USA)**  **[HIC]** | Dilated eye examination | Knowledge of DM and frequency of eye examination (OR),  Non-attendance at a diabetes education class (OR),  Not having heart disease or neuropathy,  Believe of no treatment currently available,  Lack of concern vision loss | Not mentioned | Type of the eye care provider performed last eye examination (ophthalmologist vs other) (OR),  Availability of health education programs | Physicians’ recommendation,  Focused care on high-risk group for developing DR | Availability of insurance schemes |
| **69.Sheppler, C.R. et al (2014) (USA)**  **[HIC]** | Telemedicine with non-mydriatic camera | Lack of insurance,  Believes about health insurance covering eye health care,  Cost, Time, Felt no need, Unaware of importance, Other commitments, Not care, Forgetfulness, Afraid, Lazy,  No vision problems,  Procrastination,  Health issues,  Dislike the examination | Having an insurance,  Making eye examination as top priority | Cost,  Provider availability,  Accessibility | Clinicians address potential barriers and misconceptions | Transportation, Insurance (OR) |
| **70.Shih, H.C. et al (2007) (Taiwan)**  **[HIC]** | On site indirect ophthalmoscopy after dilatation followed by single field polaroid fundus imaging | No DR,  Educational level-Illiteracy,  Income | Willingness to Pay (Mean scores),  Increased number of chronic illnesses,  Severe stage of DR,  Patients with impaired quality of life to avoid blindness,  Higher educational level,  Patient satisfaction | Cost | Not mentioned | Not mentioned |
| **71.Storey, P.P. et al (2016) (USA)**  **[HIC]** | Dilated fundus examination | Severity of DR (OR),  Ethnicity | Severe DR (OR) | Communication among the ophthalmologist and primary care physician (OR) | Showing the fundus photographs and teaching,  Identifying interventions to improve DR care,  Intervention to improve doctor-doctor communication,  Electronic medical record system,  Written communication among the ophthalmologist and primary care physician (OR) | Insurance |
| **72.Trento, M. et al (2002) (UK and Italy)**  **[HIC]** | 2F mydriatic colour photographs by digital fundus camera | Knowledge and attitude on DR,  Awareness | Spontaneous health perceptions and beliefs | Wrong assumption on patients’ level of knowledge | Group care approach,  More structured way of involving the patients,  Structured education approach than simple information during consultation | Not mentioned |
| **73.VanEjik, K.N. et al (2012) (Netherland)**  **[HIC]** | Fundoscopy and mydriatic fundus photography | Lack of awareness,  Physical disability,  No one to accompany,  Thought of not useful at old age, No interest or no time,  Lower level of education | Fear of impaired vision (OR),  Feeling obliged to attend screening-Sense of duty,  More frequent visit to health care providers and more contact,  Knowledge of detrimental effects of DR on visual acuity (OR),  Awareness of possibility of treating DR (OR) | No recommendation,  No confidence over the service provider,  Long waiting time (>30 min) | Eye screening recommendation by care provider (OR),  Trained technicians | Underserved inner city areas,  Language and financial constraints,  Active education and encouragements, |
| **74.Walker, E.A. et al (1997) (USA)**  **[HIC]** | Dilated fundus examination | Fear of denial,  Priority of the other work,  Knowledge and attitude (Waiting for symptoms),  Spirituality (faith and hope),  False believes (Early symptoms to alert them), | Internal and external motivation,  Acute situation (acute loss of vision),  Having eye problems,  Thought to keep good eye sight and find problems early | Lack of Health Education,  Cost,  Wrong interpretation of doctor not let them to go or doctor said eyes are fine,  Dilation uncomfortable,  Difficult to get an appointment | Doctors recommendation,  Recommendation by the service provider,  Health promotion materials emphasizing yearly DFE in absence of symptoms | Economic (insurance affordability)  Logistic reasons |
| **75.Yeo, S.T. et al (2012) (UK)**  **[HIC]** | Dilated fundus photography | Discrete choice over attributes | Not mentioned | Waiting time for results (OR),  Limited health care resources | Ability of the DR screening modality to detect other changes,  Explanation of results (OR),  Frequency of screening-shorter screening intervals,  Less waiting time for results,  Care as user’s preference and reassurance,  Detail information about the process (OR) | Travel time (OR) |
| **76.Yeo, S.T. et al (2012) (UK)**  **[HIC]** | Mydriatic photography | Discomfort of mydriasis,  Awareness of patients | Concerned about maintaining eye health | Time taken to give results,  Fixed appointment date,  Cost of attending screening,  Waiting time in clinic,  Discomfort from eye drops,  Inability to drive after screening,  Attitude of staffs,  Awareness of staff | Detailed information on screening process,  Explanation of results,  Reassurance of screening intervals (longer intervals),  Able to change the appointments | Travel time,  Screening location (Near home or work) |
| **77.Zhang, X. et al (2009) (USA)**  **[HIC]** | Dilated eye examination | Low income | Having DR (OR) | Not mentioned | Provision of eye care education (OR) and screening interventions | Health insurance (OR),  Rural area,  Ethnicity |
