# Supplementary material for: Systematic review on barriers and enablers for access to diabetic retinopathy screening services in different income settings
Source: PLoS One. 2019 Apr 23;14(4):e0198979. doi: 10.1371/journal.pone.0198979 (PMC6478270; doi:10.1371/journal.pone.0198979)
Supplement: S5 Table — (DOCX) [file pone.0198979.s005.docx]

**S5 Table. Quantitative Data Synthesis - Factors associated with DR screening uptake and regular follow up**

**S5 Table 1. [LIC]**

| **Study Author Name and Year** | **Participants’ characteristics** | **Variables in General** | **Results** | **Results - Further Analysis** |
| --- | --- | --- | --- | --- |
| **1.Mumba M et al 2007 (Tanzania) LIC** | Diabetics  Consecutive sample  N=316  N=114 following referral | Factors associated with ever having had a dilated fundus examination | 187 (59.1%) reported that they had undergone dilated fundus exam at some point since their diagnosis. | Factors associated with ever having had a dilated fundus examination-  -Knowledge that diabetes damages the eye  [OR 7.34 (95%CI 4.66-11.57)]  -Age  [OR 1.02 (95%CI 1.01-1.03)]  -Duration of diabetes  [OR 1.00 (95%CI 1.00-1.01)] |
| **2.Mumba M et al 2007 (Tanzania) LIC** | Diabetics  Consecutive sample  N=316  N=114 following referral | Factors associated with having had a dilated fundus examination in the past year | Only 29% had eye examination in previous year, this increased up to 47% after the ‘counselling intervention’. | Factors associated with having had a dilated fundus examination in the past year-  -Knowledge that diabetes damages the eye  [OR 19.67 (95%CI 7.01-55.20)]  -Age  [OR 1.03 (95%CI 1.02-1.04)] |
| **3.Thapa R 2012**  **(Nepal) LIC** | Diabetics  N=210 | Awareness on DR | Only 63 % of the subjects were aware that diabetes mellitus can affect the eye and result in blindness. | Awareness of diabetic retinopathy-  -Literate patients  [OR 2.74 (95%CI 1.33 - 5.63 p=0.006)]  -Living in the Kathmandu valley  [OR 2.24 (95%CI 1.08 - 4.64 P=0.030)]  -Having DM in the family  [OR 2.34 (95%CI 1.07 - 5.13 p=0.034)]  -Having a history of prior fundus evaluation elsewhere  [OR 11.94 (95%CI 5.66 - 25.18) p<0.001)] |

**S5 Table 2. [LMIC]**

| **Study Author Name and Year** | **Participants’ characteristics** | **Variables in General** | **Results** | **Results - Further Analysis** |
| --- | --- | --- | --- | --- |
| **4.Abdulsalam et al, 2018, (Nigeria) LMIC** | Service providers comprised of physicians from 4 tertiary level institutions | Knowledge among the providers | Lack of knowledge on gold standard of DR screening (knew only 4.8%)  Lack of knowledge in complications of DM  (92% were wrong) | Relation-ship of KAP of physicians on DR  Knowledge and attitude  [Correlation coefficient - r = 0.13, p=0.166]  Attitude and practice (negative relation)  [r= - 0.13, p= 0.144]  Practice and knowledge-  [r= 0.086, p= 0.385]  shows no correlation |
| **5.Adriono G et al 2011 (Indonesia)**  **LMIC** | Diabetics N=196 | Knowledge about diabetic retinopathy (score) | Mean knowledge score 4.7 (those who had eye examination) vs score 3.6 (without eye examination) [p <0.001] | Factors associated with having an eye examination –  -Higher with high knowledge score  [OR 1.52 - 95%CI 1.09 - 2.11, p = 0.01]  -Years since being diagnosed as having diabetes  [OR 1.56 (95% CI 1.09-2.78 p=0.04)] for second vs first tertile;  [OR 1.7 (95% CI 1.49-4.78 p=0.02)] for third vs first tertile |
| **6.Bamashmus MA et al 2009**  **(Yeman)**  **LMIC** | Patients with DM  Group A (Regular attendance) N=114  Group B (Irregular attendance) N=114 | Relative risk of having DR | DR was found in 47 (41.2%) and 68 (61.4%) patients, respectively.  The risk of DR, bilateral blindness and low vision disability were higher in group B.  The severity of DR was positively associated with irregularity in clinic visits (X^2^=33.56, degrees of freedom = 5, P = 0.000003). | -Relative risk of having DR  [RR - 1.51, 95% CI 1.23-2.18]  -Bilateral blindness  [RR=4.0, 95% CI 1.38-11.6]  -Low vision disability  [RR=2.53, 95% CI 1.84-3.47] |
| **7.Bamashmus MA et al 2009**  **(Yeman)**  **LMIC** | Patients with DM  Group A (Regular attendance) N=114  Group B (Irregular attendance) N=114 | Not having DR | The duration of diabetes and the regularity in clinic visits were the predictors of DR. | Not having DR in patients with DM associated with-  -Less duration of DM  <5 Years  [Adjusted OR 0.04 (95%CI 0.01–0.10 p=0.0000000001)]  5–9 Years  [Adjusted OR 0.12 (95%CI 0.05–0.30 p=0.000010)]  10–14 Years  [Adjusted OR 0.31 (95%CI 0.12–0.79 p=0.01)]  -Regular clinic visits  [Adjusted OR 0.41 (95%CI 0.2–0.77 p=0.01)] |
| **8.Islam FMA, et al 2018 (Bangladesh) LMIC** | N = 213 Patients identified with diabetes  N= 68 Patients participated in the screening program | Awareness  DR related vision loss | 68 (32%) patients participated in the DR screening program.  Diabetes related health literacy is the major factor associated with participation in DR screening. | Awareness related DM causes eye disease and uptake of screening  [OR 8.47(95% CI 3.95-18.18)]  Awareness of DR and uptake  [OR 5.15 (95% CI 1.89-14.01)]  Awareness of possibility to prevent DR related vision loss  [OR 3.15 (95% CI 1.53- 6.51)]  Having secondary or higher education associated with screening uptake  [OR 11.8 (95% CI 4.02-34.7)] |
| **9.Mwangi N. et al 2017.**  **(Kenya) LMIC** | 9 Diabetes clinics in 3 counties.  N=270  90 participants per county. | Factors that affect uptake of eye examination | Only 25.6% of participants had ever had an eye examination in their lifetime.  24.4% had been referred from the diabetes clinic for a retinal examination.  13.3% had taken a funduscopic examination in the last 12 months. | The main predictors for having ever had funduscopic examination  Referral for eye examination  [OR 20.5, 95% CI 10.2–40.9, p < 0.001).  Knowledge of diabetes eye complications [OR 2.7, 95% CI 1.5–4.8, p < 0.001]  Comorbid hypertension  [OR 1.8 95% CI 1.0–3.1 p = 0.02] |
| **10.Muecke JS et al 2008**  **(Myanmar) LMIC** | Diabetics  N=100 (50%) (Total N=200) | Likely to visit an ophthalmologist | Although 99% of GPs were aware that diabetes could result in loss of vision, 49% never examined the fundi. Although 92% realized they should visit an ophthalmologist regularly, only 57% had seen an ophthalmologist. | Less likely to visit an ophthalmologist  -Never attended school  [OR 0.24 (95%CI 0.09-0.66)]  -Diabetes for less than 2 years  [OR 0.21 (95%CI 0.9-0.44)] |
| **11.Sirinivasan NK et al 2017 (India) [LMIC]** | Diabetic patients N=288 | Association of knowledge of diabetes with the practice regarding DR  Association of awareness of DR with the practice of DR | Out of the 288, 42% had good knowledge about diabetes, but only 4.5% had good knowledge about DR.  A total of 61.1% of patients did not have periodic eye examination; most common barrier identified was lack of awareness about the necessity for this (38.5%). | Good knowledge of DM was associated with good practice of DR  [OR 3.95, 95% CI 1.97-7.94 p<0.01]  Awareness of DR with practice of DR  [OR 3.58, 95% CI 1.67-7.69, p<0.01] |

**S5 Table 3. [UMIC]**

| **Study Author Name and Year** | **Participants’ characteristics** | **Variables in General** | **Results** | **Results - Further Analysis** |
| --- | --- | --- | --- | --- |
| **12.Cetin EN et al 2013 (Turkey) UMIC** | Diabetic patients, N=514 | Awareness of diabetic retinopathy | 88.1 % aware that DM could affect eyes, 94.2% aware that DM could affect vision, only 38.8% on regular ophthalmologists follow up, awareness on laser treatment 43.8%. | Independent factors affecting visiting an ophthalmologist on regular basis –  - No DM education –  [OR 0.39 (95% CI 0.24 - 0.65)],  - duration of diabetes < 5years  [OR 0.45 (95%CI 0.26 - 0.77)] |
| **13.Hazavehei SMM et al 2010 (Iran) UMIC** | Diabetics at risk of ocular complications  N = 250 | Mean scores of patient knowledge | The knowledge and all BASNEF model components were significantly increased in experimental group after intervention. | Factors affecting the means scores of patient knowledge-  -Knowledge  [Mean 73.45 (SD 17.79) P<0.001]  -Evaluation of behavioural outcomes  [Mean 77.42 (SD 10.56) P<0.001]  -Attitude towards the behaviour  [Mean 82.00 (SD 8.32) P<0.001]  -Enabling factors  [Mean 77.66 (SD 12.19) P<0.001]  -Normative believes  [Mean 72.08 (SD 11.70) P<0.001]  -Subjective norms  [Mean 60.90 (SD 18.80) P<0.001]  -Intention towards the behaviour  [Mean 85.40 (SD 13.36) P<0.001]  -Patients’ behaviour  [Mean 78.00 (SD 17.31) P<0.001] |
| **14.Katibeh et al 2017 (2^nd^ Article) (Iran) [UMIC]** | Diabetics | patient awareness | 364 (73.4%, 95% CI: 68.6, 78.2) were unaware of the necessity of regular eye examinations. | Awareness associated with level of education  [p=0.004]  Secondary or higher education  [OR 1.88, 95% Ci 1.23-2.88]  Controlled HbA1c  [OR 2.66, 95% CI 0.80-8.82, p=0.1]  Those who had DR more likely be aware of necessity of eye examination  [OR 2.61, 95% CI 1.75-3.88, p<0.001] |
| **15.Wang D et al 2010**  **(China) UMIC** | Patients with diabetes  N=824 (92.7%)  (Total N=889) | Predictors for ever had an eye examination | 356 (43.2%) had never been examined the eye. | Potential Predictors for ever had an eye examination-  -Attendance at urban hospitals  Tertiary hospital-  [OR 6.92 (95%CI 4.16–11.53 P<0.001)]  Community hospital-  [OR 2.23 (95%CI 1.40–3.56 P=0.001)]  -Recommendation of regular eye examinations by caregivers  [OR 2.22 (95% CI 1.49 –3.31 P<0.001)]  -Having a higher DR knowledge score  [OR 1.31 (95%CI 1.14–1.51 P<0.001)]  -More concern about vision loss from diabetes  [OR 1.26 (95%CI 1.08–1.48)] P=0.004)]  -Wearing glasses regularly  [OR 2.06 (95%CI 1.19–3.57 P=0.010)]  Positive history of hypercholesterolemia  [OR 1.58 (95%CI 1.07–2.33 P=0.021)] |
| **16.Wang D et al 2010**  **(China) UMIC** | Patients with diabetes  N=824 (92.7%)  (Total N=889) | Predictors for having an eye examination in the last 12 months | 550 (66.7%) had not been examined the eye in the last year as recommended | Potential Predictors for having an eye examination in the last 12 months-  -Attendance at urban hospitals  Tertiary hospital-  [OR 3.46 (95%CI 2.13–5.64 p<0.001)]  Community hospital-  [OR 1.76 (95%CI 1.09–2.86 p= 0.021)]  -Recommendation of regular eye examinations by caregivers  [OR 2.36 (95%CI 1.29–3.59 P=0.011)]  -Having a higher DR knowledge score  [OR 1.24 (95%CI 1.09–1.42 p=0.001)]  -More concern about vision loss from diabetes  [OR 1.22 (95%CI 1.06–1.41p= 0.007)]  -Wearing glasses regularly  [OR 1.64 (95%CI 1.06–2.53 P=0.025)]  -Positive history of hypercholesterolemia  [OR 1.70 (95%CI 1.20–2.41 p=0.003)] |
| **17.Xiong Y et al 2015 (China)**  **UMIC** | Diabetics in a community  N=1120 | Awareness of diabetic retinopathy | The average score of the awareness questionnaires was 61.1. | Higher Awareness regarding DR-  -Younger patients  [OR 0.9292 (95% CI 0. 6554 - 1. 3173 p=0.0000)]  -More education  [OR 1.8396 (95% CI 0. 9825 - 3. 4442 p=0.0000)]  -Lower medical insurance reimbursement rates  [OR 1.5964 (95%CI 0.9244-2.7570 p=0.0056)]  -Longer diabetes durations  [OR 1.7500 (95%CI 1.2321-2.4856 p=0.0000)]  -On diet control  [OR 2.1485 (95%CI 1.4756-3.1284 p=0.0000)]  -More frequent exercise  [OR 1.0894 (95%CI 0.4372-2.7149 p=0.0058)]  -More severe DR stages  [OR 1.7966 (95% CI 1. 2302 - 2. 6237 p=0. 0255)] |

**S5 Table 4. [HIC]**

| **Study Author Name and Year** | **Participants’ characteristics** | **Variables in General** | **Results** | **Results - Further Analysis** |
| --- | --- | --- | --- | --- |
| **18.Basch CE et al 1999 (USA)**  **HIC** | Diabetics N=280 | Effectiveness of a health educational intervention | DF with 6 months after randomisation, rate of examination - 54.7% in intervention vs 27.3% in the control | Higher odds for eye examination status associated with-  receiving intervention –  [OR 4.3 (95% CI 2.4 - 7.8)]  Odds ratio associated with being male –  [OR 0.3 (95% CI 0. 1 - 0.9)] |
| **19.Baumeister SE et al 2015 (Germany) HIC** | Self-reported diabetics N=4308 and N=4402 | Patient characteristics related to eye care utilisation |  | Past-year eye care use decrease-  -Male  [OR 0.50 (95%CI 0.29-0.84 p<0.01)]  -Age  20-39 years  [OR 0.09 (95% CI 0.01-0.70 p<0.05)]  40-64 years  [OR 0.45 (95% CI 0.28-0.75 p<0.01)]  -Marital status  Never married  [OR 0.14 (95% CI 0.03-0.76 p<0.05)]  Married/current partnership  [OR 0.53 (95% CI 0.35-0.82 p<0.01)]  -Educational attainment  <10 years  [OR 0.37 (95%CI 0.16-0.88 p<0.05)]  10 to 13 years  [OR 0.49 (95%CI 0.31-0.77 p<0.01)]  -Employment situation  Currently employed  [OR 0.28 (95% CI 0.13-0.63 p<0.01)]  Unemployed  [OR 0.26 (95% CI 0.09-0.79 p<0.05)]  -Income 1st tertile  [OR 0.34 (95%CI 0.18-0.63 p<0.01)]  -Statutory health plan  [OR 0.52 (95%CI 0.36-0.75 p<0.01)] |
| **20.Baumeister SE et al 2015 (Germany) HIC** | Self-reported diabetics N=4308 and N=4402 | Past year eye care use by disease characteristics |  | More likely to visit an ophthalmologist-  -Time since diagnosis of diabetes >5years  [OR 0.49 (95%CI 0.31-0.80 p<0.01)]  -Treatment of diabetes with oral antidiabetic drugs and insulin  [OR 0.17 (95%CI 0.08-0.36 p<0.01)]  -High HbA1c >7%  [OR 0.44 (95%CI 0.25-0.78 p<0.01)]  -High blood pressure  [OR 0.52 (95%CI 0.35-0.78 p<0.01)]  -Dyslipidaemia  [OR 0.48 (95%CI 0.27-0.85 p<0.05)]  -Poor self-reported health  [OR 0.48 (95%CI 0.28-0.85 p<0.05)]  -Lower physical health-related quality of life  0.48 (0.29e0.78 p<0.01)]  -Lower mental health-related quality of life  [OR 0.36 (95%CI 0.20-0.68 p<0.01)]  -Number of comorbid conditions >3  [OR 0.30 (95%CI 0.17-0.53)]  -Obese >30  [OR 0.41 (95%CI 0.24-0.68 p<0.01)]  -Physical inactivity  [OR 0.43 (95%CI 0.27-0.68 p<0.01)]  -Moderate alcohol consumption  [OR 0.48 (95%CI 0.33-0.71 p<0.01)]  -Last visit to general practitioners longer than 12 months  [OR 0.55 (95%CI 0.36-0.83 p<0.01)]  -Last visit to internists longer than 12 months  [OR 0.69 (95%CI 0.39-1.24)] |
| **21.Baumeister SE et al 2015 (Germany) HIC** | Self-reported diabetics N=4308 and N=4402 | Predicting past year  eye-care utilization |  | (during 1997-2001)  Factors positively correlates with eye-care use-  -Time since diagnosis of diabetes 20 years  [OR 2.66 (95%CI 1.18-5.98 p=0.041)]  -Treatment with oral anti diabetics insulin  [OR 2.83 (95%CI 1.09-7.35 p=0.957)]  -Past-year visits to general practitioners and internists  [OR 1.55 (95%CI 0.84-2.87 p=0.161)]  Factors inversely associated with eye-care use-  -Heavy alcohol consumption  [OR 0.33 (95%CI 0.16-0.69 p=0.003)  -Mental health-related quality of life-25th percentile  [OR 1.81 (95%CI 0.98-3.34 p=0.041)]  (2008-2012)  Predictors of eye-care services use-  -Time since diagnosis of diabetes >20 years  [OR 1.44 (95%CI 0.63-3.32 p<0.001)]  -Diabetic retinopathy  [OR 3.09 (95%CI 1.26-7.56 p=0.009)]  -Diabetic nephropathy  [OR 2.71 (95%CI 0.57-12.99 p=0.197)]  -Past year visits to general practitioners or internists  [OR 2.73 (95%CI 1.47-5.05 p=0.001)]  Predictors of less likely of eye-care services use-  -Unemployment  [OR 0.47 (95%CI 0.19-1.13 p=0.091)]  -Diabetic foot  [OR 0.44 (95%CI 0.21-0.94 p=0.035)]  -HbA1c levels  [OR 0.34 (95%CI 0.15-0.77 p=0.022)] |
| **22.Bennet GH, et al 2018 (Ireland) [HIC]** | GP from Cork N=72, Patients N=147 | Referral systems | The most popular referral method was online registration (53%, 38/72), followed by a phone call (18%, 13/72), e-mail (17%, 12/72), and a letter (14%, 10/72).  11% (8/72) of general practitioners proposed that patients refer themselves to the service. | Factors that increase attendance  Older age  [OR 1.023, 95% CI 1.001 to 1.046]  Non-ocular complications of diabetes  [OR 2.741, 95% CI 1.158 to 6.489] |
| **23.Brechner RJ et al 1993 (USA) HIC** | Diabetics N=2405 | Factors associated with who received a dilated eye examination in the past year | The proportion with a dilated eye examination was 61% among diabetics at high risk of vision loss and 57% those who had diabetes for longer duration | The odds of having had a dilated eye examination in the last one year-  -Women (vs men)  [OR 1.20 (95% CI 0.95 – 1.21]  -Diabetics >70 yrs (vs 40 yrs)  [OR 1.95 (95% CI 1.47 – 2.58)]  -Family income > $50,000  [OR 1.94 (95% CI 1.29 – 2.92)]  -More than high school education  [OR 1.47 (95% CI 1.03-2.09)]  -Having attended a diabetes education class  [OR 1.54 (95% CI 1.22 – 1.94)]  -Not treated with insulin, told to have retinopathy  [OR 1.63 (95% CI 1.13-2.37)]  -Treated with insulin, never told to have retinopathy  [OR 2.57 (95% CI 1.83 - 3.61)] |
| **24.Creuzot GC et al 2014**  **(France) HIC** | Diabetic patients,  N=4699 | Attendance at subsequent ophthalmic follow up | 1,241 (79%) of recommended ophthalmic examinations were conducted. | Factors influencing good compliance with the recommended ophthalmic visit-  -Duration of diabetes <5 years  [OR 1.70 (95%CI 1.24–2.34 p<0.01)]  Factors influencing poor compliance with the recommended ophthalmic visit=  -Frequency of ophthalmic visit Less than every 2 years  [OR 0.61 (95%CI 0.42–0.87 p<0.01)] |
| **25.Dervan E et al 2008**  **Ireland**  **HIC** | Diabetic patients N=209 (77%) (Total N=271) | DF with in last one year | 12 (30%) of unscreened had not been examined within the last 12 months.  6 (15%) of unscreened had never had their eyes examined.  21 (55%) of unscreened had screened inappropriately. | Predictors of patient uptake of diabetic retinopathy screening  -Physician recommendation to have regular eye examination  [OR 1.32 (95%CI 1.11–1.58)]  -History of diabetic retinopathy or other eye disease  [OR 1.2 (95%CI 1.07–1.35)]  -Think eye examinations are needed every 6 months  [OR 1.25 (95%CI 1.12–1.40)] |
| **26.Foreman J et al 2017. (Australia) HIC** | Thirty randomly selected geographic sites  N=4836  Indigenous=1738  Non-indigenous=3098 | Adherence to screening recommendations | Adherence to screening  Non-indigenous - biennial - 77.5%  Indigenous - annual - 52.7% (p<0.001) | Greater adherence by non-indigenous Australians was associated with longer duration of diabetes  [adjusted odds ratio aOR - 1.19 per 5 years; p= 0.018]  Increasing age was associated with poorer adherence in non-Indigenous Australians  [aOR, 0.70 per decade; P=0.011]  Indigenous Australians - factors positively associated with adherence  Residing in inner regional areas  [aOR,1.66; p=0.007] and  Being male  [aOR, 1.46; p=0.018] |
| **27.Gulliford MC et al 2010**  **(UK) HIC** | Diabetics  N=31,484 | Non-attendance at screening | 7026 (22%) subjects were not screened in the period. | Factors associated with non-attendance at screening after invitation-  -Male gender  [OR 1.16 (95%CI 1.05–1.27 p=0.002)]  -Age  18–34 Years of age  [Adjusted OR 1.40 (95%CI 1.14–1.73 p=0.002)]  35–44 Years of age  [Adjusted OR 1.44 (95%CI 1.24–1.68 p<0.001)]  >85 Years of age  [Adjusted OR 0.97 (95%CI 0.77–1.24 p=0.830)]  -Deprivation quintile  Most deprived  [Adjusted OR 1.37 (95%CI 1.15–1.62 p<0.001)]  -Ethnicity  Black other  [Adjusted OR 1.72 (95%CI 1.38–2.15 p<0.001)]  Mixed  [Adjusted OR 3.97 (95%CI 3.33–4.75 p<0.001)]  Not known  [Adjusted OR 15.8 (95%CI 14.0–17.9 p<0.001)]  -Diabetes type Other and not known  [Adjusted OR 3.53 (95%CI 3.01–4.14 p<0.001)]  -Longer diabetes duration (years)  5–9Years  [Adjusted OR 1.90 (95%CI 1.65–2.19 p<0.001)]  10–14Years  [Adjusted OR 2.13 (95%CI 1.78–2.54 p<0.001)]  15–19Years  [Adjusted OR 3.11 (95%CI 2.55–3.80 p<0.001)]  >20Years  [Adjusted OR 3.40 (95%CI 2.73–4.24 p<0.001)]  Not known  [Adjusted OR 8.01 (95%CI 6.70–9.58 p<0.001)] |
| **28.Gulliford MC et al 2010**  **(UK) HIC** | Diabetics  N=31,484 | Good attendance at screening | 24458 (78%) having one or more screening episodes. | Factors associated with good-attendance at screening after invitation-  -Ethnicity  African  [Adjusted OR 0.26 (95%CI 0.18–0.37 p<0.001)]  Caribbean  [Adjusted OR 0.22 (95%CI 0.14–0.34 p<0.001)]  Other ethnicity  [Adjusted OR 0.32 (95%CI 0.20–0.50 p<0.001)]  -Age  <18 Years  [Adjusted OR 0.27 (95%CI 0.11–0.68 p=0.006)]  75–84 Years  [Adjusted OR 0.74 (95%CI 0.64–0.87 p<0.001)] |
| **29.Huang OS et al 2009 (Singapore) HIC** | General population N=3280 and n=768 diabetics | Awareness of diabetes + diabetic retinopathy and associated factors | 13.2% unaware of diabetes,  84.4% were unaware of having DR,  59.2% were unaware of vision threatening retinopathy. | Lack of awareness (regarding diabetes) associated with-  -Older age  [(60-69) years OR (Multivariable-adjusted) 10.45 (95% CI 0.22 - 0.91 p=0.03)  -Poorly controlled HbA1c  [OR (Multivariable-adjusted) 4.91 (95% CI 2.51 - 9.62) p=<0.001]  -Male gender  [OR (Multivariable-adjusted) 1.18 (95% CI 0.75 - 1.85 p=0.47)]  Lack of awareness (regarding diabetic retinopathy) associated with-  -Older age  [(70-80) years  [OR (Multivariable-adjusted) 4.63 (95% CI 1.08-19.93 p=0.04)] |
| **30.Huang OS et al 2013**  **(Singapore) HIC** | Participants with at least one of five eye conditions  N=2112 | Awareness of eye condition | 1757 (83.2%) were unaware of at least one of their eye conditions. | Factors related to unawareness of eye condition –  -older age  [Multivariable adjusted OR 1.03 (95%CI 1.02–1.04 p<0.0001)]  -Lower education (Primary or less)  [Multivariable adjusted OR 1.89 (95%CI 1.40–2.55 p<0.0001)]  -lower income (Singapore$ <2000)  [Multivariable adjusted OR 1.73 (95%CI 1.20–2.50 p=0.003)]  -Poorer literacy (Unable to write)  [Multivariable adjusted OR 1.44 (95%CI 1.02–2.05 p=0.03)]  -Higher serum glucose  [Multivariable adjusted OR 1.08 (95%CI 1.04–1.12 p<0.0001)]  Serum LDL  [Multivariable adjusted OR 1.20 (95%CI 1.06–1.36 p=0.003)]  Wears glasses of any kind no  [Multivariable adjusted OR 2.90 (95%CI 2.10–3.98 p<0.0001)]  -Had better visual acuity  [Multivariable adjusted OR 1.32 (95%CI 1.01–1.73 p=0.04)]  -Lower annual eye examination attendance  [Multivariable adjusted OR 2.08 (95%CI 1.48–2.92 p<0.0001)] |
| **31.Hwang J et al 2015**  **(Canada) HIC** | Self-reported diabetics  N=2323 | Factors associated with increased eye screening | 72% reported receiving a dilated eye examination within 2 years. | Increased eye screening associated with-  -Discussion of diabetic complications with health professionals  [OR 2.02 (95% CI 1.28–3.19 p=0.00)]  -Having private insurance  [OR 3.23 (95% CI 2.21–4.73 p=0.00)]  -Duration of diabetes longer than 10 years [OR 1.53 (95% CI 1.04–2.25 p=0.03)]  -Having visual impairment  [OR 2.60 (95% CI 1.73–3.91 p=0.00)] |
| **32.Jones HL et al 2010**  **(USA) HIC** | Adults with diabetes  N=305 | Factors associated with increased screening after a telephone intervention | Nearly all participants who obtained a DFE did so after 4 or fewer phone calls, all did so by the fifth phone call. | Factors associated with having a dilated eye exam within the intervention 6 months  -Higher baseline level of worry regarding complications  [OR 3.47 (95%CI 1.78–6.77)] |
| **33.Kreft D. et al 2018. (Germany) HIC** | Germany’s largest public insurance provider records of patients with type 2 DM.  N= 26,560 type 2 DM | To assess factors associated with DR screening uptake. | More than half of the incident cases had not seen an ophthalmologist - > 2 years (2.25 yrs). | Factors associated with a lower likelihood of DR screening  Older age (compared to 50-69 yrs)  [Hazard ratio - HR (70 - 74 yrs) = 0.93, 95% CI 0.89 - 0.97]  [HR (90+ yrs) = 0.50, 95% CI 0.42 - 0.60]  Higher disability level  [HR (disability level 3) = 0.30, 95% CI 0.25-0.36]  Factors associated with a higher likelihood of DR screening  Female sex  [HR = 1.12, 95% CI 1.08-1.15]  Six or more comorbidities  [HR = 1.26, 95% CI 1.15-1.37]  Type of DM  Moderate [HR = 1.51, 95% CI 1.46-1.56]  Severe [HR = 1.53, 95% CI 1.45-1.61]  Being enrolled in a type 2 diabetes disease management program  [HR = 1.78, 95% CI 1.69-1.87] |
| **34.Leese GP et al 2008**  **(UK) HIC** | All patients with diabetes  Diabetes patients N=15150 | Risk factors for non-attendance | 12% of the invitations to attend eye screening were missed. | *Model 1 – (All patients invited to both the mobile units and the static, hospital-based unit)*  Factors associated with failure to attend eye screening –  -Deprived areas  Most deprived areas  [OR 2.32 (95%CI 1.92–2.81)]  Second most deprived areas were  [OR 1.5 (95%CI 1.24–1.82)]  -Patients who were invited to eye vans  [OR 2.92 (95%CI 2.48–3.44)]  -Longer Duration of Diabetes  [OR 1.019 (95%CI 1.012–1.027)]  -Poor AIC control  [OR 1.253 (95%CI 1.079–1.455)]  -Poor blood pressure control  [OR 1.012 (95%CI 1.007–1.018)]  -Smoker  [OR 2.516 (95%CI 2.186–2.895)]  Factors associated with attending to eye screening-  -Constant Screening location  [OR 0.016 (95%CI 0.013–0.021)]  -Older age  [OR 0.968 (95%CI 0.965–0.972)]  *Model 2 - Only those patients invited to the mobile units)*  Factors associated with failure to attend eye screening-  -Deprived areas  Most deprived areas  [OR 1.981 (95%CI 1.573–2.495)]  Second most deprived areas were  [OR 1.414 (95%CI 1.14–1.753)]  -Longer Duration of Diabetes  [OR 1.024 (95%CI 1.015–1.033)]  -Poor AIC control  [OR 1.426 (95%CI 1.190–1.709)]  Poor blood pressure control  [OR 1.007 (95%CI 1.001–1.014)]  -Smoker  [OR 2.265 (95%CI 1.904–2.694)]  Factors associated with attending to eye screening-  -Constant Screening location  [OR 0.061 (95%CI 0.047–0.078)]  -Older age  [OR 0.964 (95%CI 0.959–0.969)] |
| **35.Legorreta AP et al 1997**  **(USA) HIC** | Patients with diabetes  N=19,397 | Increase in screening | 25% and 27% increases in patients who received DR examinations in 1995compared with 1993 and 1994, respectively. | The increase in diabetic retinal examinations-  -After the health educational intervention  [OR 1.4 (McNemars x2 = 102.7; P < 0.0001)] |
| **36.Lian JX et al 2013**  **Hong Kong) HIC** | Self-reported diabetics  N=1165 Free group  N=1052 Pay group | Factors associated with uptake of screening | Being in the pay group was negatively associated with uptake of screening (OR 0.59, 95% CI 0.47 to 0.74) | Higher uptake of screening associated with-  -Occupation  Retired  [Adjusted OR 1.53 (95% CI 1.08–2.16 p=0.016)]  Homemaker  [Adjusted OR 1.76 (95% CI 1.18–2.60 p=0.005)]  -Family income  $10,000–19,999  [Adjusted OR 1.46 (95% CI 1.02–2.09 p=0.040)]  >$20,000  [Adjusted OR 1.66 (95%CI 1.04–2.67 p=0.034)]  Low uptake of screening associated with-  -Being in the pay group  [Adjusted OR 0.59 (95% CI 0.47–0.74 p<0.001)] |
| **37.Lian J, et al, 2018, (Hong Kong)**  **[HIC]** | Diagnosed PwDM who participated in a previous RCT  Sample size= 2593 | Knowledge, awareness and perceptions of vision loss due to DR, importance of screening and frequency. | Perception of vision loss  (42.9% - 1113/2593 - worry about vision loss)  Knowledge on DM causes vision loss  (79.6% - 2063/2593)  Availability of DR treatment  (17.5%, 453/2593)  Asymptomatic nature of early DR  (11.5%, 297/2593) | Adjusted awareness and screening attendance  Worry about vision loss  [OR=1.72, 95% CI 1.31-22.26, p<0.001]  Awareness of the importance of regular eye examination  [OR=1.83, 95% CI 1.24-2.70, p=0.002]  Awareness of the frequency of eye examinations  Every year - [OR=2.64, 95% CI 1.65-4.22, P<0.001]  Every 6/12 [OR=3.27, 95% CI 1.92-5.56, P<0.001]  Did not know [OR 2.11, 95% CI 1.38-3.25, p=0.001] |
| **38.Maberley DA et al 2002**  **Canada HIC** | Diabetics N=248 | Status of a retinal examination | 85% (241) - attended a DR examination within the preceding 2 years, 42 had not. | Factors associated with good attendance of a retinal examination  (Univariate)  -Older age (>60 years)  [OR 0.09 (95%CI 0.02-0.41)]  -Longer duration of DM  5-10 years  [OR 0.25 (95%CI 0.08-0.74)]  >10 years  [OR 0.22 (95%CI 0.07-0.70)]  (Multivariate)  -Older age (>60 years)  [OR 0.10 (95%CI 0.02-0.50)]  -Longer duration of DM  5-10 years  [OR 0.24 (95%CI 0.08-0.76)]  -Community of residence-moose factory (not from Moosonee)  [OR 0.48 (95%CI 0.18-1.27)] |
| **39.Moss SE et al 1995**  **(USA) HIC** | Diabetics at primary care clinics  N=2990 | Factors associated with previous eye examination | 64% percent of the younger-onset group and 62% of the older-onset group had had a dilated eye examination in the previous year. | Factors associated with having a previous eye examination  Younger onset  -Cataract history  [OR 3.57 (95%CI 1.90 – 6.71 p<0.0001)]  -Proliferative Retinopathy  [OR 2.61 (95%CI 1.77 – 3.86 p<0.0001)]  -having told that an eye examination is needed  [OR 1.92 (95%CI 1.29 – 2.87 p<0.005)]  -Health insurance with eye examination covered  [OR 3.17 (95%CI 2.22 – 4.54 p<0.0001)]  -Thought of person with DM should have eye examination every 12 months  [OR 2.28 (95%CI 1.47 – 3.54 p<0.0005)]  Older onset  -Cataract history  [OR 2.91 (95%CI 1.91 – 4.45 p<0.0001)]  -Moderate to proliferative Retinopathy  [OR 1.91 (95%CI 1.21 – 3.01 p<0.01)]  -Health insurance with eye examination covered  [OR 3.35 (95%CI 2.19 – 5.13 p<0.0001)]  -Thought of person with DM should have eye examination every 12 months  [OR 2.62 (95%CI 1.68 – 4.08 p<0.0001)] |
| **40.Moreton R.B.R. et al 2017. (UK) [HIC]** | 79 general practices  N=21,789 invited,  of which  82.4% attended.  Oxfordshire DR screening programme | Factors that affect DR screening uptake | Uptake was 82.4% during the study period and was higher for men (83.2%) than for women (81.5%) (P = 0.001)  Uptake varied by age group (P < 0.001), being lowest in those aged 12–39 years (67%).    Uptake was higher for people invited to a general practice for screening by a mobile unit (83.5%) than for those invited for screening by a high-street optometrist (82%) (P = 0.006). | Those with GP based screening and most deprived areas are least likely to attend  Deprivation Group 1  [OR - 0.75, 95% CI 0.58-0.96]  Deprivation Group 2  [OR 0.66, 95% CI 0.53-0.96] |
| **41.Mukamel DB et al 1999**  **(USA) HIC** | Patients with diabetes  N=4410  Primary care physicians N=408 | Probability of screening in a 12-month period | 34% of patients were screened in 1993. | Factors affecting the probability of screening  in a 12-month period-  Increase in screening odds-  -Older patients  [OR 1.02 (p<0.001)]  -Patients who visit their PCPs more often  [OR 1.28 (0.001<p<0.01)]  -Living in areas of higher average education and lower percentage of blacks.  Decrease in screening odds-  -Male  [OR 0.87 (0.01<p<0.05)]  -Living in areas of higher percentage of blacks.  [OR 0.94 (0.01<p<0.05)] |
| **42.Mukamel DB et al 1999**  **(USA) HIC** | Patients with diabetes  N=4410  Primary care physicians N=408 | Probability of an annual screen in two successive years | Only 16% of diabetic patients received a true annual screening. | Factors affecting the probability of an annual  screen in two successive years-  Increase in screening odds-  -High patient expenditures per month.  [OR 1.04 (0.001<p<0.01)]  Reduce screening odds-  -Male gender  [OR 0.74 (0.01<p<0.05)] |
| **43.Munoz B et al 2008**  **(USA) HIC** | Persons Without Diabetes (N=329)  Persons with Diabetes (N=222) | Knowledge about diabetic eye disease | The level of knowledge of the adverse consequences of uncontrolled diabetes was low.  . | Predictors of knowledge that uncontrolled diabetes could cause eye disease-  -Educated up to high school or more  [OR 2.48 (95%CI 1.50-4.01 P<0.05)]  -With diabetes >1 years  [OR 4.03 (95%CI 2.41-6.76 P<0.05)]  -No diabetes, having family history  [OR 3.66 (95%CI 1.94-6.89 P<0.05)] |
| **44.Munoz B et al 2008**  **(USA) HIC** | Diabetics  Consecutive sample  N=316  N=114 following referral | Having a dilated eye examination | A total of 30% of diabetic participants had had an eye examination in the previous year | Predictors of having a dilated eye examination in the past 2 years in persons with diabetes-  -Older age  [OR 1.06 (95%CI 1.02-1.09 P<0.05)]  -Length of stay in the United States >5 years  [OR 4.14 (95%CI 1.48-11.57 P<0.05)]  -Having health insurance  [OR 3.11 (95%CI 1.41-6.89 P<0.05)]  Predictors of less likely to have a dilated eye examination in the past 2 years in persons with diabetes-  -Newly diagnosed with diabetes  [OR 0.21 (95%CI 0.06-0.85 P<0.05)] |
| **45.Orton E et al 2013 (UK) HIC** | Persons Without Diabetes (N=329)  Persons with Diabetes (N=222) | Reasons for not up taking DR screening | Of those invited, 26.1% did not make an appointment. (54.9% men). | More likely to be non-responders –  -People lived in most deprived areas  [OR 1.23 (95% CI 1.18 - 1.35, univariate)]  -Younger age (<40, male - compared to 80+ years, multivariate)  [OR 3.13 (95% CI 2.70 - 3.64)] |
| **46.Paksin Hall A et al 2013**  **(USA) HIC** | Diabetics  N= 52,386 | Receipt of annual DF | 24,198 (69.8%) reported that they had had a diabetic eye examination within the last year. | Unadjusted OR-  Increased odds of undergoing a dilated eye examination within the past year  -65 and older  [adjusted OR 3.11 (95%CI 1.46–6.62)]  -Higher income  $35,000–$49,999  [adjusted OR 1.48 (95%CI 1.27–1.72)]  >$75,000  [adjusted OR 1.55 (95%CI 1.29–1.86)]  -College graduate or higher  [adjusted OR 1.79 (95%CI 1.53–2.09)]  -Have health insurance  [adjusted OR 2.97 (95% CI 2.47–3.57)]  -Had fewer than 14 mentally unhealthy days within the past month  [adjusted OR 1.49 (95% CI 1.32–1.69)]  -Taking insulin  [adjusted OR 1.55 (95% CI 1.39–1.73)]  -Participating in diabetes management classes [adjusted OR 1.67 (95% CI 1.51–1.84)]  -Had their feet checked within the last year by a health professional  [adjusted OR 2.34 (95% CI 2.07–2.64)]  Adjusted OR-  Increased odds of undergoing a dilated eye examination within the past year  -65 and older  [adjusted OR 2.51 (95%CI 1.15–5.47)]  -Higher income  $35,000–$49,999  [adjusted OR 1.30 (95%CI 1.09–1.55)]  >$75,000  [adjusted OR 1.30 (95%CI 1.07–1.57)]  -College graduate or higher  [adjusted OR 1.55 (95%CI 1.26–1.91)]  -Have health insurance  [adjusted OR 1.75 (95% CI 1.42–2.16)]  -Had fewer than 14 mentally unhealthy days within the past month  [adjusted OR 1.22 (95% CI 1.04–1.41)]  -Taking insulin  [adjusted OR 1.44 (95% CI 1.27–1.63)]  -Participating in diabetes management classes [adjusted OR 1.40 (95% CI 1.24–1.57)]  -Had their feet checked within the last year by a health professional  [adjusted OR 1.89 (95% CI 1.67–2.13)] |
| **47.Paz SH et al 2006**  **(USA) HIC** | Self-reported diabetics  N=821 | Factors associated with compliance with ADA guidelines for vision care. | 55% not complied with the ADA vision guidelines.  DF more than 12 months ago - 64%, never had DF - 36%. | Noncompliance associated with-  -Less educated  [OR 1.5 (95%CI 1.1–2.2 p=0.0185)]  -Lack of health insurance  [OR 2.5 (95% CI 1.7–3.7 p<0.0001)]  -Have had no routine physical examination in the last 12 months  [OR 1.8 (95% CI 1.3–2.5 p=0.0003)]  -Have a glycosylated hemoglobin level >9.0%  [OR 1.7 (95% CI 1.1–2.6 p=0.0088)] |
| **48.Rim TH et al 2013**  **(Korea) HIC** | Diabetic  N=2660 | Factors associated with screening | 998 (37%) had received a diabetic retinopathy screening within one year. | Factors associated with screening for diabetes complications-  Multivariate analysis  -65 Years or older  [aOR 1.6 (95% CI 1.1-2.4 p=0.01)]  -Living in urban areas  [aOR 1.7 (95% CI 1.3-2.1 p<0.01)]  -Graduated from  Middle school  [aOR 1.5 (95% CI 1.1-2.1 p=0.01)]  High school  [aOR 1.5 (95% CI 1.1-2.1 p<0.01)]  Higher education institute  [aOR 2.8 (95% CI 1.9-4.2 p<0.01)]  -Self- reported “unhealthy” health status  [aOR 1.7 (95% CI 1.3-2.3 p<0.01)]  Univariate analysis  -Living in urban areas  [OR 1.5 (95%CI 1.2-1.8 p< 0.01)]  -Monthly house income in highest quintile  [OR 1.4 (95%CI 1.1-1.8 p< 0.01)]  -University or higher Education  [OR 1.7 (95%CI 1.3-2.2 p< 0.01)]  -Self- reported “unhealthy” health status  [OR 1.6 (95% CI 1.3-2.0 p<0.01)]  -Having co-morbidities  1-2 co-morbidities  [OR 1.3 (95%CI 1.1-1.6 p< 0.01)]  3 or more co-morbidities  [OR 1.5 (95%CI 1.2-2.0 p< 0.01) |
| **49.Saadine JB et al 2008**  **(USA) HIC** | Diabetic patients N= 2412 | Systemic factors associated with follow up | Only 2412 of 5000 (48%) had an eye examination during the baseline study enrolment period. | Systemic factors independently associated with follow-up examination within 1 year-  -Older age  [OR 1.023 (95%CI 1.012-1.034 p<0.0001)]  -Longer duration of diabetes (>15Years)  [OR 1.894 (95%CI 1.379-2.603 p<0.0001)]  -Used insulin  [OR 1.322 (95%CI 1.004-1.740 p=0.0466)] |
| **50.Saadine JB et al 2008**  **(USA) HIC** | Diabetic patients N= 2412 | Ocular and systemic factors associated with follow up |  | Ocular and systemic factors independently  associated with follow-up examination within 1 year-  -Slightly worse visual acuity (<20/40)  [OR 1.402 (95%CI 1.125-1.748 p=0.0026)]  -Slightly worse retinopathy level - Moderate  retinopathy or worse  [OR 2.172 (95%CI 1.594-2.960 p<0.0001)] |
| **51.Scanlon PH et al 2008**  **(UK) HIC** | Diabetics  N = 13304 from data set 1  N = 10,312 from data set 2 | Uptake of screening | The least deprived quintile showed a screening uptake of 76.7%, decreasing down to 67.4% in the most deprived quintile. | Probability of having been screened for diabetic retinopathy-  -Socioeconomic deprivation (Each increasing quintile of socioeconomic deprivation probability decreased)  [OR 1.11 (95%CI 1.08–1.15 P<0.001)] |
| **52.Schoenfeld ER et al 2001 (USA) HIC** | Diabetics N=2308 | Adherence to vision care guideline - factors associated with adherence | 69% had no DF eye examination in the year preceding | Factors related to non-adherence –  -last eye examination by optometrist  [OR 5.32 (95% CI 4.21 - 6.72)]  - last eye examination non-ophthalmologist  [OR 4.29 (95% CI 2.30 - 6.16)]  - less practical knowledge about diabetes  [OR 1.57 (95% CI 1.18 - 2.08)]  - No prior formal diabetes education  [OR 1.30 (95% CI 1.06 - 1.61)] |
| **53.Sheppler CR et al 2014 (USA) HIC** | Diabetic adults N=316 | Associations with self-reported adherence to annual eye examination | Reasons for adherence –  -associated with longer duration of diabetes,  -having insurance coverage and  -better glucose control | Compliance with annual eye examination-  -Insurance coverage  [OR 2.23 (95% CI 1.15 - 4.33 p =0.02)]  - Years diagnosed with diabetes  [OR 1.06 (95% CI 1.01 - 1.12 p=0.01)]  -HbA1c  [OR 0.81 (95% CI 0.68 - 0.96 p=0.01)] |
| **54.Shih HC et al 2007 (Taiwan) HIC** | Type 2 diabetics in the community - N=406 | Mean Willingness to pay for DR screening | Mean amount willingness to pay (Mean + SD) - No DR (Taiwan dollars - NTD 468.9 ± 327.7) vs Blindness (NTD 822.2 ± 192.2),  [p = 0.0005] | Highest proportion of not willing to pay in the No DR group - 40.8%.  100% willing to pay in legal blindness |
| **55.Storey PP et al 2016 (USA) HIC** | Diabetics  N=1968 | Factors associated with examination adherence | Increased adherence associated with - written communication, severity of DR, >65 years of age, smoking status, insulin use, HbA1c / blood glucose listed in chart, insurance status. Multivariate analysis - communication, severity of DR, >65 years, insulin use and HbA1c/blood glucose. | Factors associated with examination adherence-  -Written communication from ophthalmologist to PCP  [OR 1.47 (95%CI 1.11-1.94 P=0.0071)]  -Written communication from PCP to ophthalmologist  [OR 1.53 (95%CI 1.03-2.29 P=0.036)]  -Severe DR  [OR 3.56 (95%CI 2.70-4.69 P<0.0001)]  -Age older than 65  [OR 1.33 (95%CI 1.03-1.72 P=0.027)]  -Insulin use  [OR 1.40(95%CI 1.10-1.78 P=0.0061)]  -Haemoglobin AIC listed in chart  [OR 1.57 (95%CI 1.23-1.99 P=0.0002)]  -Blood glucose listed in chart  [OR 1.72 (95%CI 1.37-2.16 p<0.0001)]  Factors associated with examination non-adherence-  -Smoking  [OR 0.54 (95%CI 0.41-0.70 P<0.0001)] |
| **56.VanEjik KN et al 2012 (Netherland) HIC** | N=3236, (respondents n=1891) | Individual barriers to DR screening | 81% of the diabetics attended DR screening. | Individual incentives to DR screening –  Knowledge and instructions –  -Recommendation by the care provider  [OR 341 (95% CI 164 - 715)],  -Knowledge of effects of DR on vision  [OR 3.3 (95% CI 2.0 - 5.5)],  -Awareness of possibility of treat DR  [OR 1.6 (95% CI 0.9 - 3.0)],  -Fear of impaired visual acuity-  [OR 1.9 (95% CI 1.5 - 2.5)] |
| **57.Yeo ST et al 2012 (UK) HIC** | Diabetics N=198 | Perspectives of diabetics on DR screening |  | Proportions that responded the factors as extremely important –  -Travel time to screening venue  [23.5% (chi 2.930 (p=0.402)],  - Length of time to receive results - [31.3% (Chi 4.785 p=0.188)],  - Detail information about the processes – [40% (Chi - 0.619 p=-.892)],  - Explanation of the results –  [53.5% (Chi 0.898 p=0.826)] |
| **58.Zhang X et al 2009**  **(USA) HIC** | Self-reported diabetes  N=617 and  N=672 | Receipt of dilated eye examination | Receipt of eye care education was independently associated with receipt of dilated eye examination | Factors associated with receipt of dilated eye examination-  -Eye care education  [OR 1.59 (95% CI 1.19-2.13)]  -Aged 65 years and older  [OR 2.60 (95% CI 1.65-4.09)]  -Females  [OR 1.62 (95% CI 1.18-2.22)]  -Individuals with DR  [OR 1.89 (95% CI 1.41-2.54)].  Factors associated with no receipt of dilated eye examination-  -Without health insurance coverage  [OR 0.51 (95% CI 0.35-0.76)] |
