# Supplementary material for: Systematic review on barriers and enablers for access to diabetic retinopathy screening services in different income settings
Source: PLoS One. 2019 Apr 23;14(4):e0198979. doi: 10.1371/journal.pone.0198979 (PMC6478270; doi:10.1371/journal.pone.0198979)
Supplement: S6 Table — (DOCX) [file pone.0198979.s006.docx]

**S6 Table. Participants’ characteristics of included articles**

| **Study ID** | **Country** | **Type of Study** | **Objective** | **Study Setting** | **Sampling Strategy / Participants’ characteristics** | **Sample size (Response rate %)** | **Sample Numbers (Male: Female Ratio - %)** | **Mean age (SD) (95% CI) [Range] years** | **Mean duration of diabetes (Years) (SD) [Range]** | **Level of DR-Prevalence [or Number DR+ve]** |
| --- | --- | --- | --- | --- | --- | --- | --- | --- | --- | --- |
| **1. Abdulsalam, S. et al. (2018)** | Nigeria | Cross sectional study | To assess knowledge, attitude and practice of DR screening among physicians | Tertiary health hospitals | Participants were GPs, residents and consultants in family medicine. | Physicians  N= 110  (Response rate 95%) | Female= 26 (24.8%) | 21-30 yrs - 40%  31-40 yrs - 45%  41 yrs and above - 14% | N/A  (experience - 76% less than 5 years) | N/A |
| **2.Adriono, G. et al (2011)** | Indonesia | Cross sectional | To assess the use of eye care and its predictors among diabetic patients | Tertiary clinic and 2 community clinics | Physician diagnosed diabetics | n=198 (99%) | Female 61.5% | 58.4 (9.4) | 5.58 (6.01) | [14/198] |
| **3.Agrawal, S. et al (2005)** | India | Cross sectional | To assess the rate of non-response who referred for eye examination | Rural screening camps | Physician diagnosed diabetics | N= 23,472  Known DM n=4111 (55%)  New DM n=1076 (11.6%) | 1.27:1 | 46.5 (13) [Range 20-58] | Not mentioned | Not mentioned |
| **4.Anderson S. et al (2003)** | United Kingdom | Cross sectional | To evaluate the Feasibility and cost of screening home bound diabetics | Private sector | GP diagnosed diabetes | 80 (80%) | 1:1.46 | 80 [Range 51-97 years] | Not mentioned | [46/80] |
| **5.Bamashmus, M.A. et al (2009)** | Yeman | Cohort study | Association of regularity of visit to diabetic clinic with presence of DR and visual disability | Eye clinic | Physician diagnosed diabetics | Group A- n=114 (Type 1-28.9%)  Group B- n=114 (Type 1-28.1%) | Group A - (M-52.6%, F-47.4%) Group B - (M-43.9%, F-56.1%) | 50.01 (11.995) [Range 17-85 years] | Not mentioned | 51.1% (44.6-57.6%) [115] |
| **6.Basch, C.E. et al (1999)** | United States of America | RCT | Educational Interventions to increase ophthalmic examination rates | General medical clinics | Diagnosed diabetics from audit records | n=280  (Interventionn = 137, Control n=143) | Male 34.3% in intervention, 34.3% in control | Intervention-55.6 (12.9)  Control-53.9 (12.8) | Intervention-8.1 (7.4)  Control-7.8 (7.3) | Not mentioned |
| **7.Baumeister, S.E. et al (2015)** | Germany | Retrospective data analysis | To Study trends of barriers to receiving recommend eye care among subjects with diabetes | Population based samples | Physician diagnosed diabetics | 1^st^ group  n= 4308  2^nd^ group n=4402 | Females in 1^st^ group 2192 and 2^nd^ group 2275 | [Range 20 - 81 years] | Not mentioned | Not mentioned |
| **8. Bennet GH, et al. (2018)** | Ireland | Cross sectional | To determine the barriers to the uptake of diabetic retina screen | Tertiary hospital | Random sampling | Patients N=147  Practitioners N=72 (response rate 72/94, 76%) | Not mentioned | Not mentioned | Not mentioned | Not mentioned |
| **9.Brechner, et al (1993)** | United States of America | National survey | Factors associated with receiving recommended eye examinations for detection of DR | National level survey | Physician diagnosed diabetics | 2829 (85%)  (Type 1 n=124,  Type 2 n=2268) | Male 41% - 53% | IDDM-34.1,  NIDDM with insulin-60.6,  NIDDM without insulin-62.6 | IDDM-17.9,  NIDDM with insulin-13.4,  NIDDM without insulin-8.6 | Ever told had DR  26.2% |
| **10.Cetin, E.N. et al (2013)** | Turkey | Cross sectional | To assess the awareness of DR and the utilisation of eye care services | University clinic and primary care clinic | Diagnosed diabetics on followed up | n=514 (85%)  (Type 1-14.6%, Type not known-37.3%) | Male 211 (48.2%)  Female 226 (51.8%) | 55.2 (11.9) | 9.4 (7.7) | Not mentioned |
| **11.Creuzot, G.C. et al (2014)** | France | Cross sectional | To evaluate the effectiveness of a mobile DR screening campaign with a non-mydriatic camera to encourage diabetics to undergo a subsequent ophthalmic follow-up. | Annual campaigns | Diabetics without having ophthalmic examination during last year | n=4699 | Male 2757 (58.7%) | 67.9 (10.7) | 10.0 (9.0) | [805/4699] |
| **12.Dervan, E. et al (2008)** | Ireland | Cross sectional | To assess whether patients were receiving regular DR screening and to examine factors influencing screening uptake | Diabetes clinics | Diabetic patients were invited to two diabetic clinics | n=271 (77%) (Type1-13%) | Male 58% | 61.6 (15) | 7.7 | 28%  (with history of DR and/or other eye disease) |
| **13.Eiser, J.R. et al (2001)** | United Kingdom | Cross sectional | To investigate patients' views of screening for DR and the effects of the screening process on health beliefs and behavioural intentions | GP clinic | Diabetic patients attending the retinal screening service | n=100  (Type 1- n=12) | 53:47 | 67.0 (11.2) [Range 24-88 years] | 9 (9.4) [Range 6 weeks-44 years] | [23/100] |
| **14. Foreman, J et al. (2017)** | Australia | Cross sectional | To determine the adherence of indigenous and non-indigenous Australians with diabetes, to NHMRC eye examination guidelines. | Thirty randomly selected geographic sites  (National Eye Health Survey - National DR screening) | Self-reported diabetes | N=4836  Indigenous=1738  Non-indigenous=3098 | Indigenous 41.1% male.  Non-Indigenous 46.4% male. | Indigenous aged (40-92), mean - 55 - (SD = 10)  Non indigenous  aged (50-98),  mean - 66.6, (SD = 9.7) | Indigenous median duration= 11y  Non-indigenous median duration=10y | Not mentioned |
| **15.Gillibrand, W.P. et al (2000)** | United Kingdom | Cross sectional | To assess knowledge on eye complications | GP clinic | Diabetes not already attending an ophthalmologist | n=2386  (Insulin dependent-17.1%) | Males 53.6%  Females 46.4% | 61 (14.9) | 1-5 years (44.9%) | Not mentioned |
| **16.Glasson, NM. et al. (2017)** | Australia | Qualitative study | To assess the acceptability of a remote DR screening model | Remote Outreach DR Screening Modality | PwDM living in remote areas  Stakeholders involved in the programme | N=14 PwDM  N=9 Stake holders | PwDM 64% Female | Range (20-81) yrs | Range [<1 - 25] yrs | 1/14 Moderate NPDR  1/14 Severe NPDR  2/14 Mild NPDR |
| **17.Gulliford, M.C. et al (2010)** | United Kingdom | Retrospective data analysis | To quantify socio-economic and ethnic inequalities in diabetes retinal screening | Teaching hospitals and one district hospital | GP-registered diagnosed diabetes | n=31484  (Type 1-n=1718,  Other and not known-n=6728) | Male 16145,  Female 15339 | Not mentioned | Not mentioned | STDR 11.5% [2819] |
| **18.Hartnett, M.E. et al (2005)** | United States of America | Qualitative study | To address inadequate retinopathy screening at a largely indigent clinic and to explore perceived barriers using qualitative techniques | University health centre | Patients were recruited through a word of mouth and flyers in clinics | Diabetics n=17,  n=12 staff members, n=10 residents, n=9 ophthalmologists, n=13 PDPs) | Male 4  Female 13 | [Range 30 - 60 years] | >5 in 1/3 | Not mentioned |
| **19.Harvey, J.N. et al (2006)** | United Kingdom | Retrospective data analysis | To examine population-based retinopathy screening, barriers to achieving  comprehensive population coverage | Local health board areas | Diagnosed Diabetics | Under GP care n=3565  Optometrists Care n=8176 | Not mentioned | Not mentioned | Not mentioned | STDR [78/305] |
| **20.Hazavehei, S.M.M. et al (2010)** | Iran | Comparison of groups | Determine the effect of educational program on eye care | Diabetes care centre | Patients with NIDDM at risk of ocular complications | 100 (divided in to groups 50 each, from 250 invited) | Experimental group – male - 11, female – 39.  Control group – male -13, female - 37 | Experimental group-54.4 (7.52)  Control group-54.24 (6.52)  [Range 40-60 years] | >5yrs | Not mentioned |
| **21.Hipwell, A.E. et al (2014)** | United Kingdom | Qualitative study | Examine the experiences of patients, professionals and screeners ; their interactions  with and understandings of DRS; and how these influence uptake | GP clinic | Diabetics from GP records | n=62  (DM patients n=38)  Professionals=24) | Females 33 (53%) | Type 1 DM-49  Type 2 DM-60  Professionals-50 | Not mentioned | Not mentioned |
| **22.Huang, O.S. et al (2009)** | Singapore | Cross sectional | To assess the awareness of diabetes and diabetic retinopathy in a Singaporean Malay population | Population based survey | Physician diagnosed diabetics | General population-n= 3280  (Including n=769 diabetics) | Females (50.5% to 64.4%) | [Range 40 - 80 years] | Not mentioned | 35.7% [272/769] |
| **23.Hwang, J. et al (2015)** | Canada | National surveys | To examine the association between socioeconomic factors and ophthalmic care services/visual impairment | Community health survey | Self-reported diabetes | n=2323 (81.7% of 2933 of SLCDC responders) | Male 58.5%,  Female 41.5% | Not mentioned | Not mentioned | Not mentioned |
| **24.Islam FMA, et al. (2018)** | Bangladesh | Cross sectional study | Factors associated with participation in a DR screening program in a rural district. | Rural community clinic | Participants selected from a cross-sectional study | N=213 | Male 32% | >40 years | Not mentioned | Not mentioned |
| **25.Katibeh M. et al (2017)** | Iran | Qualitative study | To assess the national health system for management of DM, with particular focus on DR. | Work place of each stakeholder. | Identified at Ministry of Health and other professional bodies | N=15 stakeholders,  14 interviewed.  93.3% response rate. | Not mentioned | N/A | N/A | N/A |
| **26.Katibeh et al (2017) (2^nd^Article)** | Iran | Cross sectional | To determine diabetic individuals’ level of awareness about the importance of regular eye examinations | Population based (Yazd Eye Study) | Random cluster sampling | Main sample N=2098,  N=497/539 diabetics (92%) | Male: Female = 46:54 | Not mentioned | 6.71  [5.82-7.60] | [156/488] |
| **27. Kreft D. et al. (2018)** | Germany | Cohort study | To assess factors associated with DR screening uptake. | Ophthalmologist screening clinics. | Diagnosed PwDM identified in a public health insurance scheme | N= 26,560 type 2 DM | Men 12,861 48.4%  Women 13,699 51.6% | (50-69) 55.3%  (70-74) 17.2%  (75-79) 12.6%  (80-84) 8.4%  (85-89) 4.1%  (90+) 2.4% | Mean 5.2yrs  Median 5.5yrs | Not mentioned |
| **28.Khandekar, R. et al (2008)** | Oman | Cross sectional | To determine the knowledge, attitudes practices regarding eye examination for DR among non-ophthalmologist physicians | Primary health care  centres and policlinics | Physicians involved in care of diabetics | n=40  (14 family physicians, 9 hospital physicians, 1 diabetologis, 12 other doctors) | Not mentioned | Not applicable | Not applicable | Not applicable |
| **29. Lake A.J. et al (2017)** | Australia | Qualitative study | To explore screening barriers and facilitators compared to a comparator group. | Eye clinic in a community setting | Self-reported diabetes | N= 49 people with Type2 DM  (n=14 young, 35 older) | Young adults 50% female.  Older adults 50% female. | Young adults 32.6y (32.0–34.8)  Older adults  62.5y (55.9–72.8) | Young adults  1.45y  (0.3–4.5)  Older adults  13y  (2.8–15.3) | Young adults  0%  Older adults  25% |
| **30.Leese, G.P. et al (2008)** | United Kingdom | Retrospective data analysis | To identify criteria that affect uptake of diabetes retinal screening in a community screening program using mobile retinal digital photography units | GP clinic | All diabetics | n=15150 | Male 54% | 63 (15) [Range 12-102 years] | 7.34 | Not mentioned |
| **31.Lewis, K. et al (2007)** | United Kingdom | Qualitative study | To determine what factors may influence diabetic patients’ attendance at eye clinics | 2ry eye clinic in rural district  hospital and specialised  eye clinic in a 3ry hospital | Diagnosed DR patients | Patients n=48  Service providers  n= 25 | Females N=14 (29.1%) | [Range 20-72 years] | Not mentioned | Not mentioned |
| **32.Lian, J.X. et al (2013)** | China | RCT | To examine whether inverse care law operates in DR screening based on fee for service | Primary care clinic | Self-reported diabetics | Free group -n=1316 (88.5%)  Pay group-n=1277 (82.4%) | Females -  Free group 721 (54.8%)  Pay group 701 (54.9%) | Free group-64.1 (10.8)  Pay group 64 (11.2) | Free group-7.7 (7.07)  Pay group-7.6 (6.99) | Free group 25.9% [302]  Pay group 20.3% [214] |
| **33. Lian, JX. Et al, (2018)** | Hong-Kong | Cross sectional study | To assess the association between awareness of DR and actual attendance for DR screening | Two public general outpatient clinics | Diagnosed DM who participated in RCT in 2008 | N= 2593  (screening attendance - 85% - 2217/2593) | Female = 1422 (54.8%) | Mean age  64 years | Mean  7.6 years | Not mentioned |
| **34.Lindenmeyer, A. et al (2014)** | United Kingdom | Qualitative case-based study | To identify factors contributing to high or low patient uptake of retinopathy screening | Hospital, high-street opticians and GP practice | Professionals and diagnosed diabetics | n=62 patients and professionals  (38 Patients- and 9 GP practices) | Not mentioned | Not mentioned | Not mentioned | Not mentioned |
| **35. Liu Y, et al.**  **(2018)** | United States of America | Qualitative study | To characterize contextual factors affecting rural patient adherence with diabetic eye screening guidelines. | Mile Bluff- rural- multi-payer health system | Physician diagnosed type 2 diabetics  Providers | N= 20  N=9 | Male 55% | 67 years  Range  [46-86 years] | 40% -<5years  30% -  5- 19 years  30%-  20+ years | Not mentioned |
| **36.Maberley, D.A. et al (2002)** | Canada | Cohort | To explore the factors that are associated with attendance in screening examinations for DR | General hospital | All diabetics | n=248 | 34.7:65.3 | 52.9 (15.1) | Not mentioned | Not mentioned |
| **37.Moss, S.E. et al (1995)** | United States of America | Cross sectional | To estimate the compliance with guidelines on ocular examination for diabetic persons, to examine factors that affect compliance, and to determine reasons for noncompliance. | Primary care clinic | Diagnosed diabetics | n=1298  (Younger onset <30 years n=765,  Older onset >30 years n=533) | Younger onset-  Males 375, Females 380  Older onset-  Males 212,  Females 296 | Younger onset-37 [Range 14-76 years]  Older onset-71 [Range 42-98 years] | Younger onset-23  Older onset-20  [Range - 10-40 years] | [718/1298] |
| **38. Moreton R.B.R. et al (2017)** | United Kingdom | Cross sectional study | To investigate variables at the demographic and primary care practice levels that influence the uptake of diabetic retinopathy screening. | 79 general practices | GP diagnosed DM | N=21,789 invited,  of which  82.4% attended. | Male  10,303  Female 7,633  [attended] | (12-39 yrs)  n=940  (40-59 yrs) n=4727  (60-69 yrs) n=4763  (70-79 yrs) n=4727  >80 yrs  N=2805 | Not mentioned | 82.4% of invited screened, but DR+ not mentioned |
| **39.Muecke, J.S. et al (2008)** | Myanmar | Cross sectional | Evaluate the awareness of diabetes-related eye disease among GPs and diabetic patients | GP clinics | Registered diabetics | GPs n=200 (50%)  Patients n=480 Juvenile DM 1 (0.2%),  Maturity 454 (94.6%),  Missing 25 (5.2%) | GP - 49:49  Patients – Female 304 (63.5%) | GP - 47 (7.7)  Patients-57.5 (11.0) | Not mentioned | Not mentioned |
| **40.Mukamel, D.B. et al (1999)** | United States of America | Cross sectional | To identify barriers to compliance with guidelines for DR screening | Database analysis | Diagnosed with diabetes | Patients-n=4410  Primary care physicians-n=408 | Male - 55.8% | Patients 50.5 (8.7)  [Range 31 - 64] | Not mentioned | Not mentioned |
| **41.Mumba, M. et al (2007)** | Tanzania | Prospective | To measure the current use of eye department by diabetics and improvement in usage after HE intervention | Urban specialised eye centre | Diabetics at KCMC | n=316  n=114 following referral | Male 147 (46.5%) | 56.5 (13.3) | 6.8 (5.5) | Of 225 -  No DR 68 (64.1%)  NPDR 17 (16%)  PDR 1 (0.9%) |
| **42.Munoz, B. et al (2008)** | United States of America | Cross sectional | To determine gaps in knowledge and barriers to care for diabetic eye disease in Hispanic individuals | Not mentioned | Year 2000 census with and without diagnosed DM | Without Diabetes n=329  With Diabetes n=222 | Female Without DM-43%  With DM-46% | Without DM-35 (11)  With DM-48 (12) | 6.2 (SD 7) | Not mentioned |
| **43.Murgatroyd, H. et al (2006)** | United Kingdom | Cross sectional | To explore attitudes of patients towards mydriasis for DR screening | Diabetic clinics | Diabetics at clinics with and without experience on mydriasis | n=395  Group 1 (with mydriasis experience)-n=292  Group 2 (without mydriasis experience)-n=103 | not mentioned | Group 1 - 63 [Range 20-94 years]  Group 2 - 68 [Range 29-96 years] | Not mentioned | Not mentioned |
| **44.Mwangi N. et al (2017)** | Kenya | Clinic based Cross-sectional study | Identify the demand-side factors that influence uptake of eye examination | 9 Diabetes clinics in 3 counties. | Diagnosed at county clinics | N=270  90 participants per county. | Men 127 (47%)  Women 144 (53%) | 52.3 years  (SD 14.1, range 25–88 years). | 7.3 Years  (SD 5.5) | Not mentioned |
| **45. Nam perumalsamy, P. et al 2004** | India | Cross sectional | To determine current levels of knowledge, attitudes and practices regarding retinopathy in the community to aid development of appropriate health education materials | Community project | Paramedical personnel and general public (including self-reported diabetics) | Paramedics n=99 (99.5%)  Members of the community n=204 including 69 (33.8%) diabetics | Paramedics 153 (77.3%) females  Community 138 (67.6%) males | Paramedics 42.4 [Range 24-58 years]  Community 44.5 [Range 20-75 years] | Not mentioned | Not mentioned |
| **46.Newcomb, P.A. et al 1990** | United States of America | Cross section | To evaluate the factors associated with compliance following Diabetic eye screening | Mobile examination van | Diabetics | n=1878  Further care recommended n=819  Younger-onset n=445  Older onset insulin taking n=260  Older onset non-insulin taking n=114 | 49:51  Males -  Younger-onset 227 (74.9%)  Older onset insulin taking 128 (75.8%)  Older onset non-insulin taking 47 (66%) | Not mentioned | Not mentioned | Not mentioned |
| **47.Newcomb, P.A. et al 1992** | United States of America | Population based survey | To evaluate the incidence and associated risk factors for DR (provided the opportunity to evaluate an intervention to increase ophthalmologic care) | Mobile examination van | Diabetics | WESDR Intervention n=619  Control n=241 | WESDR Male – 289 (47%)  Control Male – 105 (44%) | Not mentioned | Not mentioned | WESDR 26% [161/616]  Control 24% [55/233] |
| **48.Kurji, K. et al 2013** | Kenya | Cross sectional | To assess the patient preference for DR screening with teleophthalmology or face to face  ophthalmologist evaluation | Diabetic clinic | Diagnosed diabetics in a diabetic clinic - data base | n=57 (26 responded) | Male 15(58%)  Female 11(42%) | Male-52.4  Female-46.5 | Not mentioned | Not mentioned |
| **49.Lane, M. et al 2015** | United Kingdom | Case-control | To determine whether social deprivation is a risk factor for late presentation of PDR and whether it affects their access to urgent laser treatment. | DR screening program | Known diabetics at UK national DR screening program | n=102 (n=34 cases and n=68 controls) | M:F  Case 23:11,  Control 35:31 | Case-57 (12.3)  Control-64 (18.1) | Not mentioned | R3 level in cases,  R1-2 levels in controls |
| **50.Lee, P.P. et al 1998** | United States of America | Cross sectional | To access the association between structural factors in the health care delivery system and self -reported utilization of ophthalmic services | Non-profit, staff model HMOs | Patients in the MOS had medical conditions that were clarified by physicians and reports | Diabetics N=522 | Male 44% | 59 | Not mentioned | Not mentioned |
| **51.Onakpoya, O.H. et al 2010** | Nigeria | Cross sectional | To assess the prevalence and factors influencing previous dilated eye examination in screening for retinopathy among type II diabetics. | Teaching hospital clinic | Diabetic patients receiving treatment at a University Teaching Hospital | n=83 | Female 51 (61.4%) | 57.5 (10.8) | 6.6 | DR 21.6%  No DR 62 (74.8%)  NPDR 17 (20.4%)  PDR 1 (1.2%) |
| **52.Orton, E. et al 2013** | United Kingdom | Mixed methods health equity audit | To assess equity of access to DR screening in a geographically and ethnically diverse population and determine predictors for poor uptake | Postal survey | Diagnosed diabetics invited for DR screening | n=1000  (n=809 type 2 and n=148 type 1, n=43 not known)  (43%, 435 postal response) | Returned questionnaire  Female 202  Male 232 | Men - 64 (14.1)  Women - 66.6 (15.2) | Not mentioned | Not mentioned |
| **53.Paksin Hall, A. et al 2013** | United States of America | Retrospective data analysis | To examine the variables that contribute to diabetes patients not receiving annual dilated eye examinations | System data analysis | Diabetics registered in the National survey  Diagnosis of diabetes | System data  n= 52,386 | Male - 21,348 (weighted 50.8%)  Female - 31,038 (weighted 49.2%) | Not mentioned | Not mentioned | Not mentioned |
| **54.Pasagian, M.A. et al 1997** | United States of America | Cross sectional | To study the relationship between patients’ socio demographic characteristics and their knowledge and beliefs of diabetes | Telephone interviews | Diagnosed diabetics in a clinic | n=150 | 100% Female | 59.1 (11.3) | Mean age at diagnosis 47.1 (14) | Not mentioned |
| **55.Paz, S.H. et al 2006** | United States of America | Cross sectional | To determine the prevalence of and personal factors associated with noncompliance with  American Diabetes Association (ADA) guidelines for vision care | In home and local eye centre | Self-reported diabetics from the Los Angeles Latino Eye Study | Type 2 diabetes n=821 | Females 56% | 59.8 (10.1) | Not mentioned | Not mentioned |
| **56.Puent, B.D. et al 2004** | United States of America | Telephone interview | To determine the reasons some diabetics not receiving a dilated eye exam at least every year | Optometry practice | Chart review at optometrists - diabetes diagnosis by ICD 9 codes (Diabetes - 250) | Total n=100  n=43 completed the interview | Females 52% | 56.2 (13.6-85.2) | 13.7 (1.20-37.9) | No DR 48%  NPDR 45%  PDR 4%  Unknown 3.0% |
| **57.Rim, T.H. et al 2013** | Korea | Cross sectional | Identify and determine the socio-demographic and  health related factors associated with DR screening | Survey data | Diabetic patients | n=2660 | Male 1285 (48.3%) | 62.6 (10.4) | Not mentioned | Not mentioned |
| **58.Saadine, J.B. et al 2008** | United States of America | Retrospective (medical records) | To study the process of diabetes eye care by assessing follow-up eye examinations in patients with diagnosed diabetes in a managed care organization. | Tri-Central medical service area | Diabetic patients from a database | n= 2412 (Divided into who had a follow-up examination within 1 year and > 1 year) | Female  <1 Year sample - 327 (48.6%)  >1 Year sample – 849 (51.2%) | <1 Year sample - 63.7 (11.5)  >1 Year sample - 60.4 (12.5) | <1 Year sample -10.3 (9.6)  >1 Year sample - 6.9 (8.0) | 11 – 50% |
| **59.Scanlon, P.H. et al 2008** | United Kingdom | Cross sectional | To investigate socioeconomic variations in diabetes prevalence, uptake of screening for DR, and prevalence of DR. | GP clinic | Diabetics from GDESS database | n = 13304 from data set 1  n = 10,312 from data set 2 | Not mentioned | Data set 1-64.7 (15.3)  Data set 2-66.5 | Not mentioned | Data set 2  STDR 1386 (13.4%)  Background DR 2150 (20.8%)  No DR 6776 (65.7%) |
| **60.Scanlon, P.H. et al 2016** | United Kingdom | Cross sectional | To report relationship between age at diagnosis of DM, time from registration with the screening program to first DR screening and severity of DR | National screening program | Retrospective anonymised data of diabetics | n=689025  (9.4% of 620281 - Type 1) | 54.9% :  43.1% | Not mentioned | Type 1 - 22 (IQR 12-34)  Type 2 - 59 (IQR 50-68) | Average 33% |
| **61.Schmid, K.L. et al 2003** | Australia | Cross sectional | To determine the level of awareness of diabetes and its ocular complications within the community and among the members of Diabetes Australia | Regional survey | Two samples –  One with diabetics from members of Diabetes Australia and other without diabetics from the current electoral roll | n= 500 diabetics (response rate-33.5%)  N=1000 random people (response rate-58.6%)  23.2 % type I diabetes | From 1000 sample 46% males and 54% females.  From 500 sample 53% males and 47% females | Not mentioned | 9.4 (9.7) | Not mentioned  Patient reported DR From 1000 sample 1%,  From 500 sample 16.1%. |
| **62.Schoenfeld, E.R. et al 2001** | United States of America | Cross sectional prospective  + RCT | To describe the baseline patterns of adherence to vision care guidelines and to evaluate the factors associated with non-adherence,  RCT - to evaluate the effect of an educational intervention | Community wide campaign | Physician diagnosed diabetics | n=2308 (both type 1 and 2) | Total Male 45% | 54.1 (14.3)  Median 55 [Range 18-91] | 9.6 (9.5)  Median 6  [Range 0.5-70] | 17% had DR |
| **63.Sheppler, C.R. et al 2014** | United States of America | Cross sectional | To identify variables that predict adherence with annual eye examination | GP clinic | Adult diabetics already diagnosed for an ongoing trial (criteria not specified) | n=316 | Male 45.9%  Female 54.1% | 55.7 (11.6) | 12.8 (8.1) | Not mentioned |
| **64.Shih, H.C. et al 2007** | Taiwan | Community based study | To explore the willingness to pay values for screening for DR | Community based screening | Community based screening program - diabetes diagnosis based on WHO - 1985 criteria | Adult, Type 2 diabetics in a community  n=406 (56% of 725) participated | Male 156  Female 250 | Not mentioned | Not mentioned | No DR 289 (71.2%),  NPDR 87 (21.4%),  PDR 21 (3%),  Blind 9 (2.2%) |
| **65.Sirinivasan NK, et al (2017)** | India | Cross sectional | To document Knowledge, Attitude and Practice (KAP) patterns of diabetic patients regarding diabetes and diabetic retinopathy, and to identify barriers. | Tertiary hospital | Random sampling | N=288 | Male: Female= 160:128 | Not mentioned | Not mentioned | [108/288] |
| **66. Storey, P.P. et al 2016** | United States of America | Retrospective cohort study | To evaluate the effect of written communication between an ophthalmologist and a PCP on patient adherence to diabetic eye examination recommendations | Urban eye centre | Diabetics followed up – (using a data base) | n=1968 | Female 1077 (54.75%)  Male 890 (45.25%) | >40 years  (40-64 years) - 1369 (69.56%)  ≥65 years- 599 (30.44%) | Not mentioned | Mild DR 1468 (74.59%)  Moderate DR 107 (5.44%)  Severe DR 393 (16.65%) |
| **67.Thapa, R 2012** | Nepal | Cross sectional | To investigate the demographic characteristics and awareness of DR among new cases of DM | Tertiary eye clinic | New diabetics attending to VR clinic | New cases of DM at a VR clinic n=210 | 1.38:1 | 57 (10.2) [Range 30-81 years] | Not mentioned | DR 77.6%, PDR 16.67%  CSME (36.7% - 40.5%),  Non-CSME 3.3% |
| **68.Trento, M. et al 2002** | United Kingdom and Italy | Cross sectional | To assess how diabetic patients perceive retinopathy, screening for sight-threatening lesions and their own role in preventing blindness | GP clinics and diabetic clinics | Diabetics on follow up  (screening for STDR) | Diabetic patients  n=258  (Turin 130, Wales 128) | Males in 3 groups –  W- 64 (50%)  T1- 39 (56%)  T2- 33 (55%) | Median age (25^th^-75^th^ percentile)  W- 66 (59-74)  T1 - 63 (58-69)  T2-63 (57-70) | Median (25^th^-75^th^ percentile)  W- 7 (3-14)  T1- 12 (9-16)  T2-11 (7-16) | Not mentioned |
| **69.VanEjik, K.N. et al 2012** | Netherland | Cross sectional | To examine incentives and barriers to attend DR screening | Primary care clinic | Diagnosed diabetics on follow up | Quantitative-n=2363 (73.1% of 3236)  Qualitative –  G 1 n=5  G 2 n=8  G 3 n=9  G 4 n=8 | Total n=1891  DRS attendees-  M-49.3%  F-50.7%  DRS non attendees-  M-49.4%  F-50.6% | Not mentioned | Not mentioned | Not mentioned |
| **70.Walker, E.A. et al 1997** | United States of America | Cross sectional | To assess the knowledge  and health beliefs related to preventing diabetic eye  complications | County medical centre | Diagnosed diabetics receiving care in a county medical centre | African American Diabetics n=67 (64% of 104) | Female 54 (80.5%) | 58 (12) | 12 (10) | Not mentioned |
| **71.Wang, D. et al 2010** | China | Cross sectional | To assess the use of eye care and its predictors among diabetic patients | Endocrinology clinic (urban 3ry), Medical (urban community) and a rural hospital clinic | Patients with physician-diagnosed diabete | n=824 (92.7% of 889)  Type 1 - 6% | Female 58.8% | 62.6 (12.9) | 77.9  (89.6) months | Not mentioned |
| **72.Xiong, Y. et al 2015** | China | Cross sectional | To investigate prevalence and awareness of DR and its influential factors | Community | Diabetics in a community | n=1120 | M : F - 508:612 | 58.2 [Range 36-72) years] | Not mentioned | DR 23.6%  Mild DR 17.1%  Moderate DR 5.1%  Severe DR 1.4% |
| **73.Yeo, S.T. et al 2012** | United Kingdom | Cross sectional | To determine the preferences for diabetic retinopathy screening and examine the trade-offs between frequency of screening and other service attributes | DR screening clinics | Diagnosed diabetics registered in Wales DR screening program | n=160 (86.4% of 198) | Female n=65 (40.6%) | Not mentioned | Not mentioned | Not mentioned |
| **74.Yeo, S.T. et al 2012** | United Kingdom | Cross sectional | To obtain the views about the provision of DR screening services and interval of screening | DR screening clinics | Diabetics already on a National Level Screening program | n=621 (40% of 1550) | Female 244 (40.7%) | Not mentioned | 8.5 (7.8) | Not mentioned |
| **75.Zhang, X. et al 2009 *** | United States of America | Data from Project DIRECT  (Population based) | To examine diabetic retinopathy, dilated eye examination, and eye care education among African Americans before and after a community-level public health intervention | Community based health promotion program | Self-reported a health care provider’s diagnosis of diabetes who participated in  Project DIRECT | n=1289  (617 in 1996-1997  672 in 2003-2004) | Female 63.6 % to 66.5% in different groups | Not mentioned | Not mentioned | Crude DR 41.1% - 48.4% in different groups |

*Two articles (76 and 77) are not stated here because those are review articles – not applicable to participants’ characteristics table.
